# Supplementary material for: The Mycobacterium tuberculosis methyltransferase Rv2067c manipulates host epigenetic programming to promote its own survival
Source: Nat Commun. 2023 Dec 21;14:8497. doi: 10.1038/s41467-023-43940-6 (PMC10739865; doi:10.1038/s41467-023-43940-6)
Supplement: Supplementary file 1 — Supplementary Information [file 41467_2023_43940_MOESM1_ESM.pdf]

Supplementary Information

The *Mycobacterium tuberculosis* methyltransferase Rv2067c manipulates host epigenetic programming to promote its own survival

Prakruti R Singh[1,2], Venkatareddy Dadireddy[3], Shubha Udupa[1], Shashwath Malli Kalladi[1], Somnath Shee[1,4], Sanjeev Khosla[5], Raju S Rajmani[4], Amit Singh[1,4], Suryanarayanarao Ramakumar[3], and Valakunja Nagaraja*[1,2]

[1]Department of Microbiology and Cell Biology, Indian Institute of Science (IISc), Bengaluru, India.

[2]Jawaharlal Nehru Centre for Advanced Scientific Research (JNCASR), Bengaluru, India.

[3]Department of Physics, Indian Institute of Science (IISc), Bengaluru, India.

[4]Centre for Infectious Disease Research (CIDR), Department of Microbiology and Cell Biology, Indian Institute of Science (IISc), Bengaluru, India.

[5]Council of Scientific and Industrial Research-Institute of Microbial Technology, Chandigarh (CSIR -IMTech), India.

*Corresponding author(s): vraj@iisc.ac.in

Contents

Supplementary Notes **1**

Note 1: Rv2067c structure determination 1

Note 2: Structural homologs of Rv2067c 1

Note 3: Residue conservation at the active site of Rv2067c and the proposed reaction mechanism 2

Note 4: Challenge in modeling Rv2067c - free H3 complex 3

Supplementary Methods 3
Purification of proteins 3
Rv2067c 3
DOT1L$^{2\text{-}416}$ 4
Histone H3 5
Human core histones (H3, H4, H2A and H2B) 5
Prerparation of H3-H4 dimers/tetramers 6
Preparation of Widom 601 DNA 6
Preparation of nucleosome core particles (NCPs) 7
Nuclear localization signal 8
Analytical gel filtration chromatography 8
Enzyme-substrate reaction complex model, rotation scan, and rationale 9
Construction of nucleosome-Rv2067c and nucleosome-DOT1L reaction complex models for rotation scan 10
Sequence analysis 10
Molecular dynamics simulations 11
Analysis of substrate-binding trough 11
Modeling of Rv2067c - H3 peptide complex 12
RNA and genomic DNA isolation from Mycobacteria 12
Annexin V and propidium iodide (PI) staining 12
RNA sequencing analysis 13
Software 13

Supplementary References 39

Supplementary Tables

Supplementary Table 1: Data collection and refinement statistics 14
Supplementary Table 2: Sequences of genomic co-ordinates identified by H3K79me3 ChIP 15

Supplementary Figures

Supplementary Figure 1: Rv2067c methylates histone H3 at lysine 79 16
Supplementary Figure 2: Rv2067c methylates histone H3 at lysine 79 18

Supplementary Figure 3: Secretion and localization of Rv2067c 20

Supplementary Figure 4: Structural comparison between Rv2067c and DOT1L 21

Supplementary Figure 5: Interactions between DOT1L and ubiquitinated nucleosome 22

Supplementary Figure 6: Essential structural elements of DOT1L and their comparison to Rv2067c . 23

Supplementary Figure 7: Active site of DOT1L 24

Supplementary Figure 8: Residue conservation in the substrate-binding trough of Rv2067c 25

Supplementary Figure 9: Mode of substrate binding in protein MTases 26

Supplementary Figure 10: Side chain temperature factors of Rv2067c active site residues 27

Supplementary Figure 11: The backbone RMSF of Rv2067c monomer A 28

Supplementary Figure 12: Histone fold 29

Supplementary Figure 13: Rv2067c modulates DOT1L expression. 30

Supplementary Figure 14: Downstream events consequent to H3K79 methylation by Rv2067c . . . 31

Supplementary Figure 15: Interactions between Rv2067c and SAH 33

Supplementary Figure 16: A representative electron density map of Rv2067c crystal structure . . . 34

Supplementary Figure 17: Structural homologs of Rv2067c 35

Supplementary Figure 18: Proposed catalytic mechanism of Rv2067c 36

Supplementary Figure 19: Enzyme-substrate complex reaction center model used for rotaton scan . 37

Supplementary Figure 20: Benchmarking the rotation scan on nucleosome-DOT1L complex 38

Supplementary Notes

Note 1: Rv2067c structure determination

The initial structure of Rv2067c was determined by experimental phasing using iodine single wavelength anomalous dispersion (SAD) to 3.25 Å resolution. A better resolution structure of 2.40 Å was determined from a native dataset using the structure model obtained from experimental phasing (Methods). The asymmetric unit (ASU) contains two molecules of Rv2067c related by a 2-fold non-crystallographic symmetry (Fig. 3a) and share an interface area of 1565.1 Å^2 (p-value = 0.00159, calculated by PISA[1]) indicating that the crystallographic dimer is biologically relevant. The dimeric state was also confirmed by analytical gel filtration chromatography (Supplementary Fig. 4a). The two molecules in the ASU were modeled as chain A (residues 16-208, 215-371 and 378-405) and chain B (residues 18-371 and 376-405). The remaining residues could not be modeled due to the missing electron density, an indicative of flexibility in those regions. These regions include the N-terminal segment (residues 1-15 of chain A and residues 1-17 of chain B), residues 209-214 of chain A (the corresponding residues in chain B are structured due to crystal contacts), and residues 372-377 (chain A) and residues 372-375 (chain B). The Cα root mean square distance (RMSD) between two chains is 0.265 Å (over 376 pairs of Cα atoms). An unmodeled electron density in the Fourier difference map (F_o - F_c, at 3.0 σ level) was observed at the SAM-binding site (Supplementary Fig. 15a). This density was modeled as S-adenosyl-L-homocysteine (SAH) due to the resulting negative density at the donor methyl carbon when modeled as SAM. However, no co-substrate (SAM or SAH) was used during purification or crystallization. Co-substrates are known to get co-purified with methyltransferases[2–4]. SAH binds in a pocket formed by a conserved motif GxG (63-GCG-65) and its interactions with protein are shown in Supplementary Fig. 15b). The protein backbone dihedrals are within the Ramachandran favored (98.14%) and allowed (1.86%) regions. A representative electron density map is shown for Rv2067c crystal structure (Supplementary Fig. 16).

Note 2: Structural homologs of Rv2067c

To find structures similar to Rv2067c, a structural similarity search was carried out using DALI server[5]. DALI search yielded two proteins with structures similar to Rv2067c: protein lysine methyltransferase 1 from *Rickettsia prowazekii* (PKMT1, PDB: 5DPD [https://doi.org/10.2210/pdb5DPD/pdb]) and protein lysine methyltransferase 2 from *Rickettsia typhi* (PKMT2, PDB: 5DPL [https://doi.org/10.2210/pdb5DPL/pdb]) with Z-scores 27.3 and 23.2 and coverage of 342 and 345 residues, respectively[6]. PKMT1 and PKMT2 methylate multiple lysines on the Outer membrane protein B (OmpB) of rickettsial species. PKMT1 mainly performs monomethylation whereas PKMT2 predominantly trimethylates OmpB[7]. The overall topology and domain organization between Rv2067c and rickettsial PKMTs are similar except

that rickettsial PKMTs contain an extra domain, the middle domain, which is equivalent to the CTD of Rv2067c in terms of fold and its position in the tertiary structure (Supplementary Fig. 17). The substrate-binding cleft (termed trough, in the case of Rv2067c) of PKMT1/2 is wider compared to Rv2067c and was characterized to be involved in substrate binding[6].

Note 3: Residue conservation at the active site of Rv2067c and the proposed reaction mechanism

To identify the residues important for substrate binding and catalysis, we mapped the residue conservation onto the structure of Rv2067c with two different sets of homologous sequences, set-A and set-B. Sequences from set-A are from mycobacterial species and are less diverse. Whereas, set-B contains sequences from all genera and are more diverse (Supplementary Methods). A highly conserved patch encompassing the SAM-binding site was observed for both the sets of sequences.This conserved patch can be divided into two halves (Supplementary Fig. 8a, b). The SAM-binding pocket half that contains GxG motif (63-GCG-65) and D85 which interact with SAM (Supplementary Fig. 15b) and the SAM-facing half that contains residues Y20, Y160, R165, D221, T222, and Q228 (Supplementary Fig. 8b; middle panel). The SAM-facing residues occlude the region opposite to the SAM methyl group and are likely to be a part of the active site region that interacts with the substrate lysine, H3K79 (Fig. 4b). A multiple sequence alignment of a representative sequences from set-B is shown in Supplementary Fig. 8c. The conservation is either moderate or poor in the rest of the substrate-binding trough (Supplementary Fig. 8a, b).

The conserved patch also contains putative catalytic residues H133, H134 and D221. The H133 and H134 are present at the base of SAM-binding pocket , and D221 is part of SAM-facing half of the conserved patch (Supplementary Fig. 8b; middle panel). The positions of these histidines are also conserved in a non-homologous rebeccamycin sugar 4'-O-MTase (RebM)[8]. The corresponding residues in RebM are H140 and H141. Both sequences, Rv2067c and RebM, contain a conserved aspartate (Rv2067c-D221 and RebM-D166), but the residue positions are not conserved on the primary structure (Supplementary Fig. 18a). Despite that, in the tertiary structures of Rv2067c and RebM, the aspartates are positioned in close proximity to interact with histidines (Supplementary Fig. 18b). In RebM, the role of these residues in catalysis has been established[8]. Based on this, we propose a catalytic mechanism for H3K79 methylation by Rv2067c. Rv2067c follows an acid/base mediated nucleophilic substitution (S_N2) mechanism for the transfer of methyl group. The substrate lysine (H3K79) is deprotonated by a general base, histidine (H133/H134), to form a nucleophile that attacks the methyl group of SAM, leading to the transfer of the methyl group from SAM to H3K79 (Supplementary Fig. 18c).

Note 4: Challenge in modeling Rv2067c - free H3 complex

All the core histones (H3, H4, H2A, and H2B) assume a histone fold (Supplementary Fig. 12a) in the presence of their partner histones (H2A:H2B and H3:H4). In the cell, free histones are accompanied by chaperones[9]. The tertiary structures of free histones in the absence of partner histones or in complex with histone-binding chaperones are not available (www.rcsb.org). Izumi, Y. et al[10,11] have mapped the residue-wise secondary structure elements (helix, strand, or coil) onto the primary structure of histone H3. The size and position of the secondary structure elements in the free H3 are different from that of the histone fold (Supplementary Fig. 12b) indicating that free histones might not assume a tertiary structure as it is seen in the histone fold (Supplementary Fig. 12a). Thus, it is inappropriate to model protein complexes containing free H3 based on the globular histone fold structure of H3.

Supplementary Methods

Purification of proteins

Rv2067c

Rv2067c was cloned in EcoRV digested pMyNT vector and expressed in *Mycobacterium smegmatis* MC^2 155 (*M.smeg*). *M.smeg* cells expressing Rv2067c were grown in 7H9 media (Difco) supplemented with 0.4% glucose (Sigma) and 0.05% Tween 80 (Sigma). The culture was induced at OD_{600} of 0.6 with 2% acetamide (Sigma) for 6 hr at 37°C. Cells were resuspended in lysis buffer (10 mM Tris-HCl, pH 7.4, 5% v/v glycerol, 100 mM NaCl, 1 mM β-mercaptoethanol (BME)) containing 1 mM phenylmethylsulfonyl fluoride (PMSF) and 10 mM imidazole and lysed using French Press (SIM AMINCO) followed by short pulses of sonication (LSL SECFROID). The cell lysate was clarified by centrifugation at 15,000 $\times$ g for 30 min and the supernatant was incubated with Ni-NTA resin (G-Biosciences). The resin was washed with lysis buffer containing 40 mM of imidazole and the protein was eluted with lysis buffer containing 250 mM of imidazole. Eluted fractions were checked on 12% SDS-PAGE. Pure proteins fractions were pooled, dialysed, and concentrated. Rv2067c RxR mutant was cloned in pMyNT vector and purified from *M.smeg* using the above protocol.

For crystallization, the Rv2067c gene was subcloned into the pMyNT vector to remove the cloning artifacts that arose from the vector backbone of the above Rv2067c construct. Rv2067c gene was amplified from the above construct using primers (Rv2067c_FP_struc and Rv2067c_RF_struc, Supplementary Data 2) and assembled into a linearized (with NcoI and BamHI) pMyNT vector using NEBuilder® HiFi DNA Assembly mix (NEB). The new construct, pMyNT-Rv2067c-2, contains an N-terminal His-tag followed by a tobacco etch virus (TEV) protease cleavage site and

Rv2067c open reading frame. Protein was expressed from pMyNT-Rv2067c-2 in *M.smeg* and purified as described above. The His-tag was removed using TEV protease in a cleavage buffer (50 mM Tris-HCl, pH 8.0, 50 mM NaCl, 1 mM BME) during overnight dialysis at room temperature. The cleaved tag was removed by passing the cleavage reaction through the Ni-NTA column. Protein was further purified by anion exchange chromatography. Protein was loaded onto Mono-Q HiTrap (GE Healthcare) 5 mL column equilibrate with low salt buffer (20 mM Tris-HCl, pH 8.0, 50 mM NaCl, and 2.5 mM BME) and washed with 5 column volumes of low salt buffer. Protein was eluted with a linear gradient of 50 mM - 1000 mM NaCl. The peak, containing Rv2067c, fractions were pooled, concentrated, and the buffer was exchanged to a storage buffer (10 mM Tris-HCl, pH 8.0, 100 mM NaCl, 2 mM BME) using a 10 kDa molecular weight cutoff (MWCO) centrifugal filter (Amicon Ultra 15 mL, Millipore, USA). Protein was immediately used for crystallization trials or snap frozen in liquid nitrogen and stored at -80°C for future use.

DOT1L$^{2\text{-}416}$

Human DOT1L (Disruptor of Telomeric Silencing 1-Like histone lysine methyltransferase) construct (pEW3: pET32a Dot1L(2-416)) was purchased from Addgene, USA (Plasmid #124098, https://www.addgene.org/124098). DOT1L$^{2\text{-}416}$ was expressed and purified by following the protocol described by Worden, E. J., et al[12]. In brief, Rosetta2(DE3) cells were transformed with pEW3: pET32a Dot1L(2-416) plasmid DNA and grown in 1 litre of 2×YT broth containing 100 μg/mL ampicillin at 37°C while shaking. At OD_{600} 0.8-1.0, culture flasks were transferred to 18°C and induced with 0.5 mM β-D-1-thiogalactopyranoside (IPTG) for overnight. Cells were pelleted at 5,400 × g and resuspended in buffer A (50 mM Tris-HCl pH 8.0, 750 mM NaCl, 5% v/v glycerol, and 2.5 mM BME) containing 1 mM PMSF and 15 mM imidazole. Resuspension was lysed by sonication and clarified by centrifugation at 20,000 × g for 30 min at 4°C. The supernatent was loaded onto NiNTA resin (G-Bioscience) and washed with buffer A containing 30 mM imidazole and eluted with buffer A containing 250 mM imidazole. Trx-His tag was removed by TEV protease in a cleavage buffer (50 mM Tris-HCl, pH 8.0, 100 mM NaCl, and 2.5 mM BME) at 4°C, overnight. Digestion was checked on SDS-PAGE gel, and cleaved tag and TEV protease were removed by passing the digested sample through HisTrap 1 mL (GE Healthcare, USA) column. The flow-through was concentrated to 2 mL using Amicon Ultra 10,000 Da cutoff filter and diluted to 6 mL with no-salt buffer (50 mM sodium phosphate, pH 7.4, 10% v/v glycerol, 2.5 mM BME). 5 mL of diluted sample was loaded onto 1 mL HiTrap SP HP column (GE Healthcare, USA) and washed with 50 mM NaCl buffer (5% high-salt buffer (no-salt buffer containing 1 M NaCl) + 95% no-salt buffer). Protein was eluted with a salt gradient of 50 - 1000 mM NaCl. The peak fractions containing pure DOT1L$^{2\text{-}416}$ were pooled, buffer exchanged to storage buffer (20 mM Tris-HCl, pH 8.0, 100 mM NaCl, 1 mM Tris(2-carboxyethyl)phosphine hydrochloride (TCEP)), concentrated to 1.65 mg/mL and aliquots were stored at -80°C after snap freezing in liquid nitrogen.

Histone H3

Histone H3 cloned in NdeI - HindIII restriction sites of pET28b(+) vector was purified from *E. coli* BL21 (DE3) using 0.3 mM isopropyl IPTG induction for 4 hr at 37°C. Cells were resuspended in lysis buffer (50 mM Tris pH 7.4, 250 mM NaCl, and 0.1% sarcosine). Lysed cells were centrifuged at 15,000 × g for 30 min, the supernatant was incubated with Ni-NTA beads. The protein was eluted in an elution buffer containing 350 mM of imidazole. Pure proteins fractions were pooled, dialysed, and concentrated.

Human core histones (H3, H4, H2A and H2B)

Human core histone proteins H3, H4, H2A, and H2B were expressed from the constructs pET21a-H3, pET3a-H4, pET21a-H2A, and pET21a-H2B, respectively. *E. coli* BL21(DE3) Rosetta2 cells harboring the above plasmids were grown in 2×YT broth (1.0% yeast extract, 1.6% w/v tryptone and 0.5% w/v NaCl) containing 0.1% w/v glucose, ampicillin (100 μg/mL), and chloramphenicol (25 μg/mL) at 37℃. Cells were induced at OD_{600} between 0.6 and 0.8 for 2 hr (H3 and H4) or 3 hr (H2A and H2B) with 0.3 mM IPTG. Cells were harvested by centrifugation at 6,000 × g and resuspended in a resuspension buffer (50 mM Tris-HCl, pH 7.4, 100 mM NaCl, 1 mM PMSF, and 2.5 mM BME). Cell lysis was carried out by alternate freeze-thaw cycles followed by sonication. Lysate was centrifuged at 23,000 × g at 4° C for 20 min. The pellet containing inclusion bodies was washed twice with wash buffer (50 mM Tris-HCl, pH 7.4, 100 mM NaCl, 1 mM Na-EDTA, 1 mM PMSF, and 2.5 mM BME) containing 1% v/v Triton X-100 followed by two washes without TritonX-100. Histones were purified from these washed inclusion bodies by acid-extraction[13]. In brief, inclusion bodies were homogenized in 10 mL of 0.25 N HCl and incubated at -20℃ for 30 min. The insoluble portion was removed by centrifugation at 27,000 × g, 4℃ for 10 min and the supernatant containing histones was neutralized with 0.125 volumes of 2 M Tris. All the histones were dialysed overnight against water containing 2 mM BME at 4°C using a 6,000 Da MWCO dialysis membrane. The dialysed histones were lyophilized and stored at -20℃ for future use. For MTase assays, either acid-extracted histones were further purified or histone from washed inclusion bodies were purified by gel filtration followed by cation-exchange chromatography as described[14], with modifications. Each acid-extracted and lyophilized histone was dissoved in 5 mL unfolding buffer (20 mM Tris-HCl, pH 7.5, 7 M guanidinium chloride, 10 mM BME), centrifuged at 15,000 × g and the supernatent was loaded onto Superdex 200 pg 120 mL gel filtration column pre-equilibrated with SAU-1000 buffer (20 mM sodium acetate, pH 5.2, 7 M urea, 1 M NaCl, 1 mM EDTA and 5 mM BME) at 1 mL/min flow rate. The peak fractions containing histone were pooled and dialysed against low-salt buffer (50 mM Tris-HCl, pH 7.4, 50 mM NaCl and 2.5 mM BME) using 3,500 Da dialysis membrane at 4°C, overnight. Histone aggregates were removed by centrifugation and loaded onto HiTrap SP HP 5

mL cation exchange column pre-equilibrated with low-salt buffer. The column was washed with wash buffer (50 mM Tris-HCl, pH 7.4, 150 mM NaCl and 2.5 mM BME) and the protein was eluted with a gradient of 150 - 1000 mM NaCl. Fractions with pure histones were pooled and dialysed against water containing 2 mM BME and stored at a final concentration of 2.0 mg/mL (4°C or -20°C). Alternatively, to purify histones from inclusion bodies, washed inclusion bodies were dissolved in unfolding buffer (20 mM Tris-HCl, pH 7.5, 7 M guanidinium chloride, 10 mM BME) and the debris was removed by centrifugation at 27,000 × g, at 20°C. The histones from the supernantent were purified by gel filtration and cation exchange chromatography as described above.

Prerparation of H3-H4 dimers/tetramers

To prepare H3-H4 dimers/tetramers, equimolar quantities of lyophilized H3 and H4 were dissolved in 4 mL of unfolding buffer (20 mM Tris-HCl, pH 7.5, 7 M guanidinium chloride, 10 mM BME) and dialysed against refolding buffer (10 mM Tris-HCl, pH 7.5, 2 M NaCl, 1 mM Na-EDTA, 5 mM BME) in a 3,500 Da cutoff dialysis membrane at 4°C, overnight. Histone aggregates were removed by centrifugation at 15,000 × g and loaded onto Superdex 200 pg 120 mL gel filtration column pre-equilibrated with refolding buffer at 1 mL/min. Peak fractions containing H3-H4 dimers/tetramers were pooled, concentrated and stored at -20°C in the presence of 50% glycerol.

Preparation of Widom 601 DNA

For preparing the nucleosome core particles (NCPs), a 145 bp strong nucleosomal positioning DNA sequence (Widom 601)[15] was used. pUC57-Widom 601 plasmid containing eight repeats of Widom 601 sequence flanked by EcoRV sites was produced in *E. coli* Top10 cells. Plasmid isolation, EcoRV digestion, and purification of the Widom 601 sequence were carried out as per the protocol described by Dyer, P. N. et al[16]. Single colony of *E. coli* Top10 cells harboring pUC57-Widom 601 plasmid DNA was inoculated into 5 mL of 2×YT broth containing 0.1% glucose and 100 μg/mL ampicillin (hence forth referred as 2×YT broth) and grown overnight at 37°C. Preculture was prepared by inoculating 5 mL of overnight culture into 100 mL of 2×YT broth and incubated at 37°C while shaking. At OD_{600} of ∼0.6, 10 mL × 8 of preculuture was added to 500 mL × 8 of 2× YT broth in 2 litres flasks and incubated at 37°C, overnight while shaking. Cells were pelleted by centrifugation at 5,400 × g, room temperature, into 4 bottles. Cell pellet in each bottle was resuspended in 30 mL of alkaline lysis solution-I (50 mM glucose, 25 mM Tris-HCl, pH 8.0 and 10 mM EDTA). 60 mL of alkaline lysis solution-II (0.2 N NaOH and 1% w/v sodium dodecylsulfate (SDS)) was added to resuspended cells, mixed thoroughly and kept on ice for 10 min with intermittent mixing for complete lysis of cells. 105 mL of ice-cold alkaline lysis solution-III (4 M potassium acetate and 2 N acetic acid) added to lysis, mixed and incubated on ice for 10 - 15 min. The neutralized lysate was clarified by centrifugation at 10,000 × g,

4°C for 20 min. The supernatent was filtered through a polypropylene gravity flow column connected to vaccum manifold. Plasmid was precipitated by addition of 0.5 volumes of isopropanol to the filtered supernatent following incubation at room temperature for 30 min. Precipitated plasmid was pelleted by centrifugation at 10,000 $\times$ g at 20°C. The pellet was dissolved in 50 mL of TE10/50 (10 mM Tris-HCl, pH 8.0 and 50 mM Na-EDTA) while shaking at 37°C with the addition of 140 μL of 10 mg/mL RNase A, until RNA is completely degraded. RNA degradation was checked on agarose gel by electrophoresis. The undissolved pellet was separated by centrifugation at 10,000 $\times$ g for 20 min, at room temperature. Every 20 mL of plasmid was extracted into aqueous phase with 10 mL of phenol by centrifugation at 27,000 $\times$ g for 20 min at 20°C, until white middle layer disappeared. The aqueous phase (45 mL) was extracted with 25 mL of CIA (24:1 v/v of chloroform and isoamylalcohol) by centrifugation at 12,000 $\times$ g for 5 min at 20°C. Plasmid in the aqueous phase was precipitated by addition of 1/5 volume of 4 M NaCl and 2/5 volumes of 40% polyethylene glycol 6,000 (PEG 6,000) by mixing at 37°C for 5 min followed by incubation on ice for 3 h. Plasmid was pelleted by centrifugation at 3,000 $\times$ g for 20 min at 4°C. The pellet was dissolved in 5 mL of TE10/0.1 (10 mM Tris-HCl, pH 8.0 and 0.1 mM Na-EDTA) and checked on agarose gel. The plasmid was extracted twice with CIA. The plasmid DNA was ethanol precipitated by addition of 1/10 volume of sodium acetate (pH 5.2) and 2.5 volumes of ice cold absolute ethanol following incubation at -20°C for 1 h. The precipitate was seperated by centrifugation at 15,000 $\times$ g and the pellet was washed with 70% v/v ice cold ethanol and dried at 37°C, overnight. The dried pellet was dissolved in 5 mL of autoclaved water by incubating at 37°C while shaking. Plasmid DNA was checked on agarose gel.

For excising the 145 bp Widom 601 fragments, six digestion reactions were setup, each containing 1 mg of plasmid in 1 mL 1$\times$ CutSmart® Buffer using 60 U of *Eco*RV-HF (NEB, USA), at 37°C for over 24 h. Completion of digestion was checked on agarose gel. The plasmid backbone was precipitated by addition of 0.192 volumes of 4 M NaCl and 0.346 volumes of 40% PEG 6,000 followed by incubation on ice for 1 h and spin at 27,000 $\times$ g, 4°C, for 20 min. The supernatent containing the 145 bp Widom 601 DNA was precipitated with 2.5 volumes of ice cold ethanol. The pellet was collected by centrifugation at 15,000 $\times$ g, 4°C and dissolved in 1 mL of TE10/0.1. The quality of the 145 bp DNA was checked on agarose gel and the concentration was estimated using NanoDrop spectrophotometer (Thermo Fisher SCIENTIFIC). Processing of $\sim$6 mg of plasmid DNA yielded 0.7 mg of 145 bp DNA. 100,000×g

Preparation of nucleosome core particles (NCPs)

Histone octamers were refolded from core histones using the salt dialysis method as described[14]. Since all the four histone prepared by acid-extraction method contained impurities, the estimation of concentrations was not accurate. Thus, the apparent quantities of H2A and H2B were kept 1.5 molar excess to H3 and H4 during reconstitution to

ease the purification of octamers from histone tetramers and hexamers by size exclusion chromatography[17]. All four histones were dissolved in unfolding buffer (20 mM Tris-HCl pH, 7.5, 6 M guanidinium chloride, 5 mM DTT) and dialysed three times against refolding buffer (10 mM Tris-HCl, pH 7.5, 2 M NaCl, 1 mM Na-EDTA, 5 mM BME) at 4℃. The precipitated protein was removed by centrifugation at 22,000 $\times$ g, 4℃ for 30 min. The supernatant was concentrated and loaded onto Superdex 200 pg 120 mL gel filtration column pre-equilibrated with refolding buffer, at 0.8 mL/min flow rate. Fractions were checked on SDS-PAGE (Supplementary Fig. 1g). Fractions with equimolar concentration of all four histones were pooled, concentration was determined and stored at -20℃ with 50% glycerol.

NCPs were reconstituted from octamers and Widom 601 DNA using microscale reconstitution protocol[16]. For reconstitution, 1.66 µg of DNA was mixed with 2.4 µg of octamers (molar ratio of DNA to octamers 1:1.2) to a final 2 M NaCl concentration in 10 µL volume and incubated on ice for 30 min. The salt concentration was slowly reduced to 100 mM in the reconstitution mix by sequential addition of 10 µL, 5 µL, 5 µL, 70 µL and 100 µL of 10 mM Tris-HCl, pH 7.6 with 1 h incubation on the ice at every step. Multiple microscale preparations were pooled and concentrated using a centrifugal filter. The quality of the preparation was assessed on 6% native PAGE (Supplementary Fig. 1h).

Nuclear localization signal

The presence of Rv2067c both in the nucleus and the cytoplasm suggested nucleo-cytoplasmic shuttling of the protein. To examine the Nuclear Localization Signal (NLS) in Rv2067c, a prediction tool, DeepLoc2.0[18] was used. The C-terminal deletion mutants were generated for the SFB-tagged Rv2067c (Rv2067c-Δ30 and Rv2067c-Δ55) and transfected in HEK cells. 24 h post-transfection, cells were washed with phosphate-buffered saline (PBS), fixed using 4% paraformaldehyde, permeabilized with 0.1% Triton X-100 and blocked with 2% BSA. FLAG was used as the primary antibody and Alexa Fluor conjugated antibody was used as a secondary antibody. Nuclei were stained with diamidino-2-phenylindole (DAPI) dye and the cells were examined by confocal microscopy.

Analytical gel filtration chromatography

For analytical gel filtration, Rv2067c was loaded onto a 24 mL Superdex 200 increase 10/300 GL column (GE Healthcare). The standard curve was prepared from the retention volumes of ribonuclease A (13.7 kDa), ovalbumin (43 kDa), conalbumin (75 kDa), aldolase (158 kDa), ferritin (440 kDa) and used for determining the molecular weight (M_r) of Rv2067c from its retention volume by linear interpolation.

Enzyme-substrate reaction complex model, rotation scan, and rationale

A model of an enzyme-substrate reaction complex for an enzymatic reaction was constructed with respect to protein lysine methylation. This model depicts the complex between the enzyme and its substrate in an imminent enzymatic reaction i.e., when the reaction is about to take place. The following assumptions were made for constructing the enzyme-substrate reaction complex model. During any enzymatic reaction, an enzyme-substrate complex is formed without steric hindrance between the enzyme and its substrate and is pre-deterministic for a given enzyme-substrate complex. In an imminent reaction, the reacting atoms from both the enzyme and its substrate must come in contact for a reaction to take place. Protein lysine methylation (a SAM-dependent methylation) follows a bimolecular nucleophilic substitution (S_N2) reaction mechanism where the methyl acceptor atom of the substrate, the ζ-nitrogen (NZ^{SUB}), attacks methyl carbon of SAM (CE^{SAM}), nucleophilically. In an imminent reaction, NZ^{SUB} lies on an axis that passes through the scissile bond, the bond between sulfur (SD^{SAM}) and CE^{SAM} atoms, and is in contact with CE^{SAM} at a van der Waals (*vdW*) contact distance of 3.30 Å[19–21] (Supplementary Fig. 19). Here, we termed the position of the NZ^{SUB} atom as the reaction center of a reaction complex. All the possible orientations of the substrate with respect to its enzyme, in a reaction complex, were determined with the help of Euler's rotation theorem[22]. The substrate was rotated about the reaction center, while the enzyme was fixed, using a rotation matrix (R), given by equation 1.

$$R = \begin{bmatrix} cos(\beta)cos(\gamma) & -sin(\gamma)cos(\beta) & sin(\beta) \\ sin(\alpha)sin(\beta)cos(\gamma) + sin(\gamma)cos(\alpha) & -sin(\alpha)sin(\beta)sin(\gamma) + cos(\alpha)cos(\gamma) & -sin(\alpha)cos(\beta) \\ sin(\alpha)sin(\gamma) - sin(\beta)cos(\alpha)cos(\gamma) & sin(\alpha)cos(\gamma) + sin(\beta)sin(\gamma)cos(\alpha) & cos(\alpha)cos(\beta) \end{bmatrix} \quad (1)$$

The α, β and γ are the Tait-Bryan angles of elemental rotations about x, y, and z axes, respectively. A range of angles $\alpha = -180 - +180$, $\beta = -90 - +90$ and $\gamma = -180 - +180$, that constitutes a rotation scan, is sufficient to obtain all the possible orientations of the substrate with respect to the enzyme. Prior to the rotation scan, the reaction center was shifted to the origin to make it rotation invariant, about which either substrate or enzyme is rotated. All the transformations were carried out in a Cartesian coordinate system. For each rotation (a combination of α, β and γ), the total number of clashing atoms (TCA) between the enzyme and its substrate were calculated using the criterion given by equation 2[21].

$$d_{ab} \leq r_{(vdW,a)} + r_{(vdW,b)} - 0.42 \Rightarrow Clash(a,b) \quad (2)$$

The d_{ab} is the distance between the atom a of the substrate and the atom b of the enzyme, and $r_{(vdW,a)}$ and $r_{(vdW,b)}$ are the *vdW* radii of atoms a and b, respectively. Atom pairs (ab) that follow the clash criterion were added up to TCA. The TCA was used as a metric to choose the orientation with minimal clashes. For simplicity, all hydrogen atoms were

excluded from the calculations. The *vdW* radii for atoms were chosen from Word, J. M. et al[21]. Carbonyl carbon *vdW* radius was set same as *vdW* radius of non-carbonyl carbon. Rotation scan was implemented using Python script[23] and is available at https://doi.org/10.5281/zenodo.8352903 and https://github.com/Venkat-Dadi/Rotation_Scan.

Construction of nucleosome-Rv2067c and nucleosome-DOT1L reaction complex models for rotation scan

The nucleosome-Rv2067c reaction complex model was constructed by imposing the assumptions described for the enzyme-substrate reaction complex model. Coordinates for Rv2067c and nucleosome were taken from the crystal structure of Rv2067c and cryo-electron microscopy structure of UbNuc-DOT1L active-state complex (PDB: 6NJ9 [https://doi.org/10.2210/pdb6NJ9/pdb]), respectively. In the Rv2067c structure, SAM was modeled in place of SAH using Coot[24]. The lysine analog, norleucine (Nle), at the H3K79 position (H3K79Nle) in 6NJ9 was mutated, in silico, to an extended rotamer of lysine. The reaction center was defined within Rv2067c. Coordinates of the Rv2067c and nucleosome were transformed such that the reaction center and the NZ atom of the H3K79 (NZ^{H3K79}) of nucleosome were at the origin. The initial orientations of Rv2067c and nucleosome, in the nucleosome-Rv2067c complex model, were set by transforming the centroids of Rv2067c and nucleosome onto the positive and negative y-axes, respectively. The active-state complex of UbNuc-DOT1L (PDB: 6NJ9) was used for benchmarking the rotation scan method. To construct the nucleosome-DOT1L reaction complex, all ubiquitin and one DOT1L molecule (chain M) were removed from 6NJ9. H3K79Nle was mutated to lysine, in silico. The position of NZH3K79 (methyl acceptor) was treated as reaction center and was moved to the origin. Three different starting models of Nuc-DOT1L with randomly oriented DOT1L, that are different from the experimental complex (6NJ9), were generated. All the models were subjected to rotation scan. The TCA were plotted as a function of any two elemental rotation angles (α, β or γ) while the third angle denotes the minimum TCA value. In a benchmarking test, all the three randomly oriented DOT1L molecules could generate DOT1L binding conformation that is seen in nucleososme-DOT1L complex, with the least number of clashing atoms, in a rotation scan (Supplementary Fig. 20).

Sequence analysis

Homologous protein sequences of Rv2067c were obtained by BLAST[25] search against NCBI Reference Protein (RefSeq_Protein) database. BLAST hits with more than 80% sequence coverage resulted in a minimum sequence identity of ∼23% were used for analysis. Two sequence sets (A and B) were generated from these hits. Set A contains sequences only from mycobacterial species clustered at 80% identity and set B contains all hits clustered at 80% identity. CD-HIT[26] was used for clustering. Multiple sequence alignment (MSA) for both the sets was generated

using Clustal Omega[27]. Residue-wise conservation scores were calculated from MSA using ConSurf[28] with default parameters and the conservation scores were mapped onto Rv2067c structure. A few selected sequences from set B were used to generate representative MSA and the MSA was rendered using Jalview[29].

Molecular dynamics simulations

The crystal structure of the Rv2067c dimer with cofactor, SAM, was used for simulations. Prior to simulations, missing loops were built and SAH was replaced with SAM using Coot[24]. The missing N-terminus residues (chain A: residues 1-15 and chain B: residues 1-17) were modeled by grafting these residues from the Rv2067c model generated using AlphaFold[30]. Thus modeled Rv2067c-SAM dimer was solvated with TIP3P water model[31] in a dodecahedron box with 1.2 nm padding from the protein atoms. The charge of the system was neutralized while keeping the concentration of Na^+ and Cl^- ions at 0.15 M. Simulation system was parameterized using CHARMM36m force field[32] which also contains force field parameters for SAM. Energy minimization (50,000 steps) was carried out with the steepest-descent method followed by equilibration in NVT and NPT ensembles, 100 ps each, with positional restraints (1000 $kJ \cdot mol^{-1} \cdot nm^{-2}$). Unrestrained production simulations of 100 ns were carried out in an NPT ensemble at 300 K temperature and 1 bar pressure. The temperature was maintained using velocity rescaling scheme[33] and pressure with Parrinello-Rahman barostat[34]. The neighbor search was carried out using Verlet cutoff-scheme with a neighbor list updated every 40 steps and van der Waals interactions were calculated up to a 1.2 nm radius. The long-range electrostatics (1.2 nm cut-off distance) were computed using the Particle Mesh Ewald method with cubic interpolation and 1.6 nm Fourier spacing. Bonds to hydrogen atoms were constrained using the LINCS algorithm. Two femtosecond time steps were used for integration. The simulation trajectory was saved at 2 ps time intervals. Simulations were carried out using GPU-accelerated Gromacs 2021.2[35]. The trajectory was processed using tools in Gromacs package and CPPTRAJ[36] from the Amber package (http://ambermd.org).

Analysis of substrate-binding trough

MD trajectory was corrected for periodic boundary conditions. Each monomer of the dimer was written to a separate trajectory, at 10 ps time intervals, without water molecules and ions. Frames of the trajectory were aligned to the crystal structure using three different sets of backbone atoms (CA, C, N, O, and H) viz. all residues (407-aa; residues 1-407), seven-β-strand core (7BS-aa; residues 58-62, 80-85, 106-110, 125-129, 154-160, 246-251, and 293-299) and 7BB-aa and part of LSD (7BS + LSD part; residues 58-62, 80-85, 106-110, 125-129, 154-160, 246-251, 293-299, 161-175, 201-207, and 215-244). The aligned trajectory with minimum RMSF (root mean square fluctuations) around the putative active site region i.e., 7BS-aa + LSD part was chosen (Supplementary Fig. 11) for calculation of volumetric

density map of the substrate-binding trough using POVME 3.0[37]. A custom inclusion volume of the grid with 1.0 Å spacing encompassing the substrate-binding trough was defined for volume measurement in POVME 3.0. The grid was set at 0.5 Å spacing in the case of crystal structure. Volumetric density analysis was carried out for monomer A (chain A) of Rv2067c structure. The simulation movie was prepared using ChimeraX[38].

Modeling of Rv2067c - H3 peptide complex

H3 peptide (73-EIAQDFKTDLR-83) was manually modeled, using Coot[24], into the substrate-binding trough of Rv2067c in two binding modes, based on the criteria followed for the reaction center and the methyl acceptor residue binding to the protein methyltransferases (Supplementary Fig. 9). The substrate lysine (H3K79) was placed nearly perpendicular to the long axis of SAM with a spatial constraint while the NZ^{H3K79} atom lies at the reaction center. The complete peptide was built by the addition of amino acid residues to the N- and C-termini of thus placed lysine. In one mode peptide orients in the direction of N- to C-termini whereas in the other it is C- to N-termini, along the trough. Two peptides with one mode per monomer of Rv2067c dimer were built. Thus built Rv2067c-H3 peptide complex was parameterized using amber FF14SBforce field[39] and energy minimized, in a vacuum, using *pmemd.cuda* module of Amber package[40]. Force- filed parameters for SAM were obtained from Saez, D. A. et al[41].

RNA and genomic DNA isolation from Mycobacteria

For RNA and genomic DNA isolation, *Mtb* strains were grown to an OD_{600} of 0.6-0.8. Cells were harvested and washed once with PBS. RNA extraction was conducted using the FastRNA® Pro Blue Kit (MP Biomedicals, USA) in accordance with the manufacturer's instructions. For genomic DNA isolation, bacteria were heat killed and DNA was extracted using CTAB, followed by phenol extraction and ethanol precipitation[42].

Annexin V and propidium iodide (PI) staining

THP1 macrophages seeded at a density of 0.5×10^6 were infected with Wt*Mtb*, ΔRv2067c, and ΔRv2067c:comp. Cell death by apoptosis or necrosis in macrophages infected with different strains was quantified using Dead Cell Apoptosis Kits with Annexin V for Flow Cytometry (Invitrogen). Apoptosis was measured as Annexin V-FITC single positive (Q4) and necrosis as PI single positive (Q1). The flow cytometry assays were performed using FACSAria Fusion (BD Biosciences) and the data were analysed using FACSDiva.

RNA sequencing analysis

Data quality was checked using FastQC[43] and MultiCQ[44] software. The data were checked for base call quality distribution. Raw sequence reads were processed to remove adapter sequences and low-quality bases using fastp[43]. The QC passed reads were mapped onto indexed Human reference genome (GRCh38.p7) using STAR2[45] aligner. Gene level expression values were obtained as read counts using featureCounts software[46].

Multistep analysis was performed to identify differentially regulated genes by Rv2067c. First, a differentially expressed gene-set was identified for macrophages infected with *M.smeg* expressing Rv2067c at 24 h.p.i in comparison to 4 h.p.i. Similarly, a gene-set was identified for macrophages infected with *M.smeg*. Next, the two gene sets were compared to identify deregulated genes by Rv2067c post 24 h.p.i. Differential expression analysis was carried out using edgeR[47] package after normalizing the data based on trimmed mean of M (TMM) values.

Software

Structures were visualized using ChimeraX[38]. Matplotlib was used for data plotting. Figures and schematics were made using Inkscape.

Supplementary Tables

Supplementary Table 1: Data collection and refinement statistics

	**Rv2067c (Iodine-SAD)**	**Rv2067c-SAH (Native)** PDB: 8HKR
**Data collection**		
Space group	$P4_12_12$	$P4_12_12$
Cell dimensions		
a, b, c (Å)	110.91, 110.91, 217.55	109.15, 109.15, 216.61
α, β, γ (°)	90, 90, 90	90, 90, 90
Wavelength (Å)	1.700	1.700
Resolution (Å)	49.41 - 3.25 (3.366 - 3.25)*	48.74 - 2.40 (2.486 - 2.40)
R_{sym} or R_{merge}	0.1502 (0.9732)	0.08841 (2.074)
$I / \sigma(I)$	12.90 (1.80)	25.92 (1.73)
Completeness (%)	99.88 (99.80)	99.96 (100.00)
Redundency	13.6 (9.8)	25.4 (25.6)
**Refinement**		
Resolution (Å)		48.74 - 2.40 (2.486 - 2.40)
No. reflections		51978 (5129)
R_{work}/R_{work}		0.1914/0.2129
No. atoms		
Protein		6120
Ligand/ion		10
Water		49
B-factors (Å^2)		
Protein		69.84
Ligand/ion		82.48
Water		58.72
RMS deviations		
Bond lengths (Å)		0.017
Bond angles (°)		2.22

* Values in parentheses are for highest-resolution shell.

Supplementary Table 2: Sequences of genomic co-ordinates identified by H3K79me3 ChIP

Genomic co-ordinate	Sequence	Gene
chr11:<br>95,249,951 - 95,250,066	GTGGTGGTGCATGCCTTTAATCCCAGCTACTTGGGAGGCAGAGGCAGGAG<br>AATCGCTTGAACCCAGAAGGCAGAGGTTGCAGTGAGCCAAGATTGTGCCA<br>CTGCACTCCAGCCTGG	*SESTRIN3*
chr12:<br>29,668,687 - 29,668,938	CTGACCTCAGGTGATCCACCCACCTTGGCCTCCCAAAGTGCTGGGATTAC<br>AGGCATGAGCCACTGCTCCTGGCCCCAGCTTCTAATTAATTAAAAGTGAT<br>AAAGATCATTTCAATATCTTCTTCAGTTCATACAGTTCTACAATGAAGGC<br>TTTTCATACAAATGAATGAAGTTAACAGTGCACAATTTTTGAAAGTCCTT<br>TTAATAAGCACGCTTTTAAAAGTTAACGCATGTAGTGATTGCTGCAGGTT<br>GA	*TMTC1*
chr15:<br>98,626,422 - 98,626,672	CCTTTGGCAATCCCCAGGCTCTCCTCAAAGCCTATTTTAGAAGGAGCTCA<br>CCACTCCTGCCCATTTGTGGGTTGACTTTGCACCTATGATGTTTCTTCTC<br>CCTCATGGCCCCAGGCTGCTCACCTCTGGCCCACAGCGTTTAATTAGATG<br>GATATTGAGGATTGAATGGGTCTGTACTTGAGGAAGGGCAGAAGTATTTC<br>ATGGCAAAGAGAATGACAGTAGCAAAAACTAAGAGGCTGGAAAGAGCTGC<br>G	*IGF1R*
chr16:<br>3,577,049 - 3,577,328	CCCTCCCCAGAGTAGCTCAGGCCTTAAGGCCACTGTCCTTCCTGCCAGCA<br>TCCCTTCTCCCTCCCCTGCCCTGCACTGAGGTCACCTGGACCCAACTTAC<br>CTCCCGGGCCTCGATGCTGCTCCAGGGACAGCAAGACTGGGGGGCCTGGG<br>GGCGTCCATCTCCATGCTCCTGGGCTCAGCCCTGCTCCAGCTGCGTGGTG<br>GTAGATGCCCTGGGAATCCCTGTGCCAGCCTGAGTTCTCAGGACCAGGGA<br>TCAGGGCACTTACCACGCCAACCAACCAAC	*NLRC3*
chr18:<br>27,008,662 - 27,008,899	TGCGAGGCTAGGAGGCAGTCGTTCATTCAACTGCTCCCCCTGTCCTCTTG<br>TATCTATCTCTTTTCCCTACCTATCTCGGTTGCTTTCTTATTCCTTTAGC<br>ACCTGCCACAGTGCTGGGCTCACAAGAAGTATTCTGGAATACTGGCATTC<br>CATTCTTCTGCCATCATCAGGAGAGTTGAATCTTCAGTGGCCCTTCAATC<br>TTGAGACAGGATTGCAGCAGGTTCTTGTTTTTTTGGTT	*CHST9*

Supplementary Figures

a

LC-MS/MS

Sample name	Protein found in database	Entry name	Calculated MW	Score	Seq. cov.	Note
CH	Uncharacterized protein Rv2067c OS=Mycobacterium tuberculosis (strain ATCC 25618 / H37Rv) GN=Rv2067c PE=1 SV=1	Y2067_MYCTU	46243	151	10%	

```
1    MTDDHPRADI VSRQYHRWLY PHPIADLEAW TTANWEWFDP VHSHRILWPD
51   REYRPDLDIL IAGCGTNQAA IFAFTNRAAK VVAIDISRPA LDHQQYLKDK
101  HGLANLELHL LPIEELATLG RDFDLVVSTG VLHHLADPRA GMKELAHCLR
151  RDGVVAAMLY GKYGRIGVEL LGSVFRDLGL GQDDASIKLA KEAISLLPTY
201  HPLRNYLTKA RDLLSDSALV DTFLHGRQRS YTVEECVDLV TSAGLVFQGW
251  FHKAPYYPHD FFVPNSEFYA AVNTLPEVKA WSVMERLETL NATHLFMACR
301  RDRPKEQYTI DFSTVAALDY VPLMRTRCGV SGTDMFWPGW RMAPSPAQLA
351  FLQQVDGRRT IREIAGCVAR TGEPSGGSLA DLEEFGRKLF QSLWRLDFVA
401  VALPASG
```

b

VTDDHPRADIVSRQYHRWLYPHPIADLEAWTTANWEWFDPVHSHRILWPDREYRPDLDILIA**GCG**TNQAAIFAFTNRAAKV
VAIDISRPALDHQQYLKDKHGLANLELHLLPIEELATLGRDFDLVVSTGVLHHLADPRAGMKELAHCLRRDGVVAAMLYGK
YGRIGVELLGSVFRDLGLGQDDASIKLAKEAISLLPTYHPLRNYLTKARDLLSDSALVDTFLHGRQRSYTVEECVDLVTAGLV
FQGWFHKAPYYPHDFFVPNSEFYAAVNTLPEVKAWSVMERLETLNATHLFMACRRDRPKEQYTIDFSTVAALDYVPLMRTR
CGVSGTDMFWPGWRMAPSPAQLAFLQQVDGRRTIREIAGCVARTGEPSGGSLADLEEFGRKLFQSLWRLDFVAVALPASG

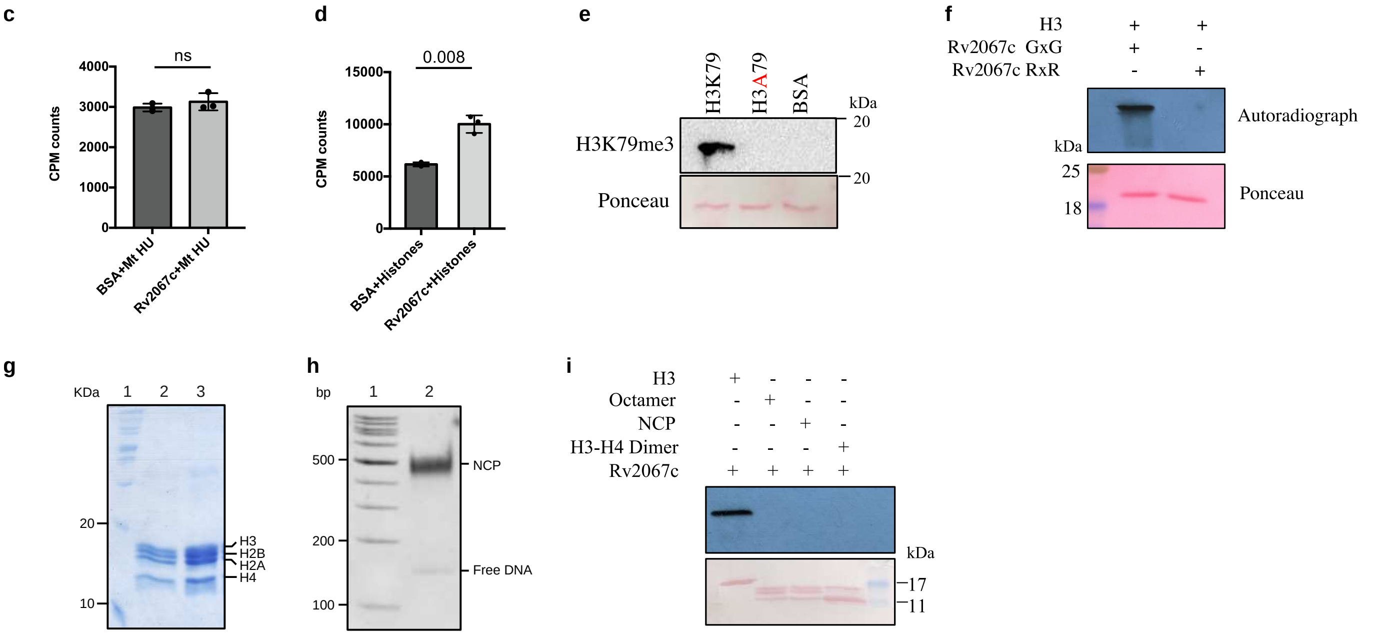

Supplementary Figure 1: Rv2067c methylates histone H3 at lysine 79. a, Rv2067c was immunoprecipitated from *Mycobacterium tuberculosis* (*Mtb*) H37Ra cell lysates using affinity purified MtHU antibody. The unique tryptic digests specific to Rv2067c identified by LC-MS/MS are shown in bold and underlined (n=1). **b,** Protein sequence of Rv2067c. Putative methyltransferase (MTase) domain identified using NCBI conserved domain search is underlined, and S-Adenosyl-Lmethionine (SAM) binding motif is marked in bold. **c** and **d,** Graph depicts scintillation counts (counts per minute - CPM) for in vitro MTase assays with MtHU and salt extracted histones from THP1 monocyte, respectively, as substrates, Rv2067c as MTase and tritiated SAM (^{3}H-SAM) as a methyl group donor. BSA was kept as negative control. n = 3 independent experiments. Data is plotted as mean and error bars represent SD. P-value depicted on the graph was calculated using unpaired two tailed Student's t-test; ns - not significant. **e,** Western blot for in vitro MTase assay with recombinant H3 and H3A79 mutant protein as substrate and Rv2067c as MTase. Blot was probed with H3K79me3 antibody. Ponceau staining of blot was used as loading control. **f,** Autoradiograph showing methylation activity of recombinant Rv2067c and Rv2067c RxR with H3 as substrate and tritiated SAM as a methyl group donor. Ponceau staining was used as loading control. **g,** Gel picture depicts reconstituted human histone

octamers. Lane 1: protein marker, Lane 2,3: fractions of size exclusion chromatography. **h,** Nucleosome preparation from octamers and Widom 601 DNA by micro batch reconstitution method. Lane1-100 bp ladder; Lane2 -reconstituted nucleosomes. **i,** Autoradiograph shows methylation activity of Rv2067c with recombinant H3, octamers, nucleosomal core particles (NCPs) and H3-H4 dimer as substrate and tritiated SAM as a methyl group donor. Ponceau staining was used as loading control. Source data are provided as a Source Data file.

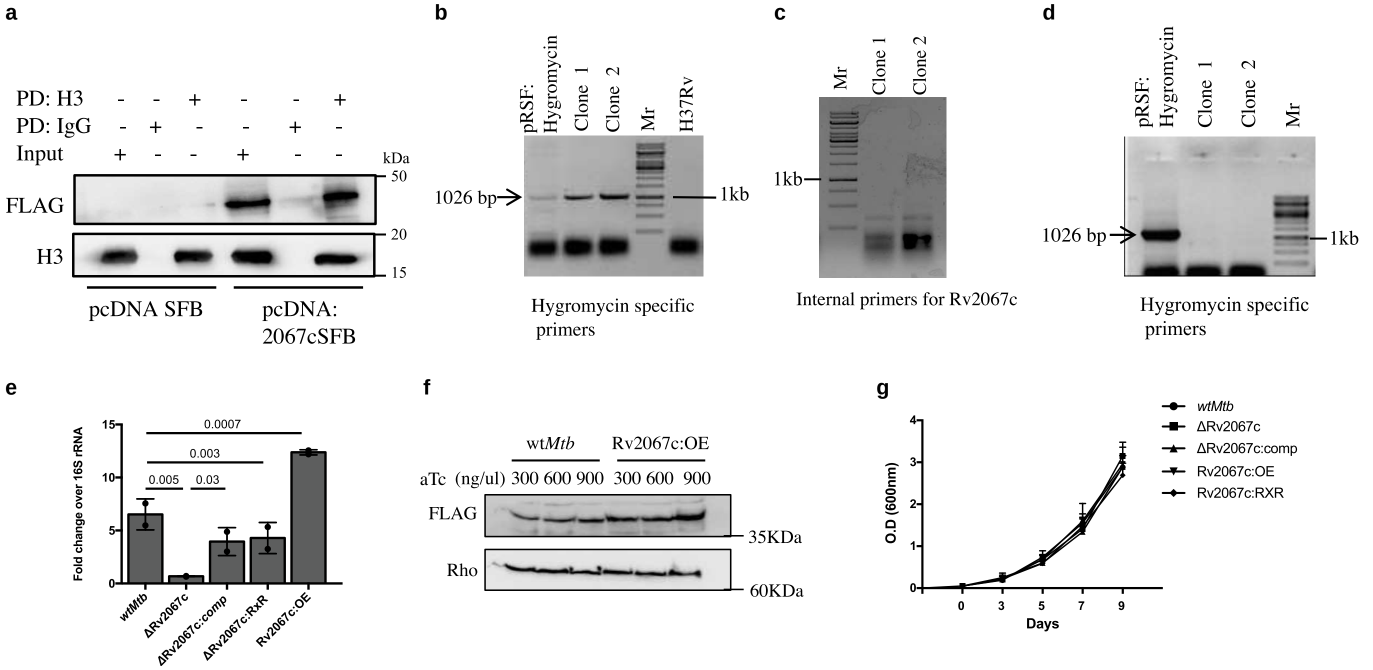

Supplementary Figure 2: Rv2067c methylates histone H3 at Lysine 79. a, Western blot depicts interaction between Rv2067c and H3 by immunoprecipitation performed with H3 antibody on HEK293T transfected with pcDNA: Rv2067cSFB or pcDNA SFB (control) constructs. 5% of whole cell lysate was kept as Input. IP was also performed with IgG as an antibody control and blots were probed with antibodies as indicated. **b** and **c,** PCR amplification using specific primers (as indicated below the agarose gel) for screening of ΔRv2067c mutant. Gel picture depicts results for two clones. Clones positive for homologous recombination gives a pcr product of 1026 bp with hygromycin specific primers and do not show amplification with Rv2067c specific primers. pRSF:Hygromycin plasmid was kept as positive control for PCR (Lane 1), Mr: 1 kb ladder. **d,** Agarose gel depicts unmarking of hygromycin gene. Unmarked ΔRv2067c positive clones do not give amplification with hygromycin specific primers. pRSF:Hygromycin was kept as positive control for PCR (Lane 1), Mr: 1 kb ladder. **e,** Bar graph shows expression of Rv2067c in Wt*Mtb*, ΔRv2067c, ΔRv2067c:comp, ΔRv2067c:RxR and Rv2067c:OE strains. Levels were normalized against *Mtb* 16S rRNA. n = 2 independent experiments. Data is plotted as mean and error bars represent SD. P-values depicted on the graph were calculated using unpaired two tailed Student's t-test. **f,** Immunoblot depicts over expression of Rv2067c in Rv2067c:OE strain in an anhydrotetracycline (aTc) dependent manner. Blot was probed with FLAG antibody for Rv2067c. Rho was used as loading control. **g,** Growth curve of strains generated for the study. n = 2 independent experiments. Data is plotted as mean and error bars represent SD. SFB: S-protein, FLAG, streptavidin-binding peptide; comp: complemented. Source data are provided as a Source Data file.

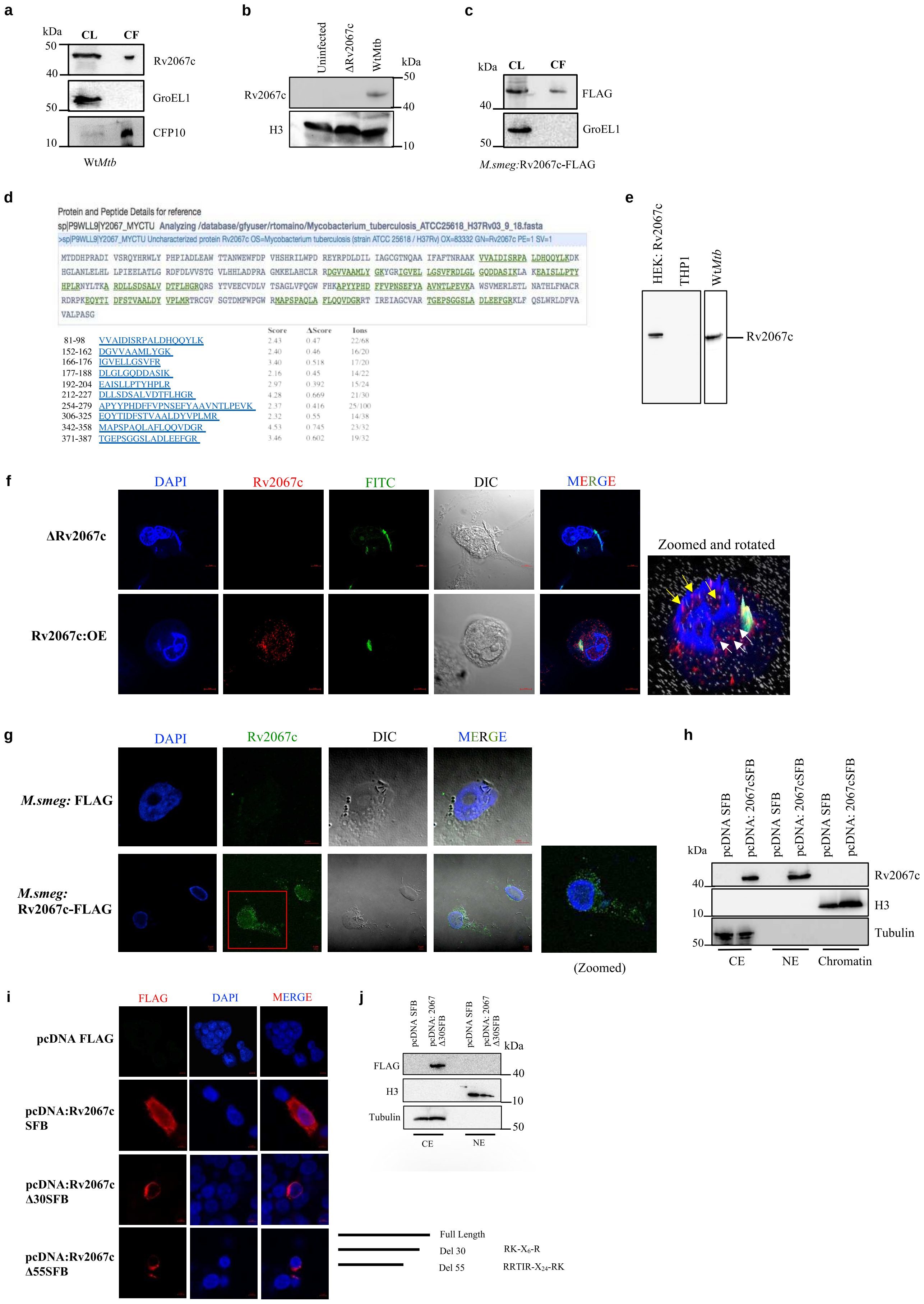

a
kDa
CL
CF
50
40
Rv2067c
GroEL1
50
CFP10
10
WtMtb
b
Uninfected
ΔRv2067c
WtMtb
kDa
50
Rv2067c
40
H3
10
c
kDa
CL
CF
FLAG
40
GroEL1
50
M.smeg:Rv2067c-FLAG
d
Protein and Peptide Details for reference
sp|P9WLL9|Y2067_MYCTU Analyzing /database/gfyuser/rtomaino/Mycobacterium_tuberculosis_ATCC25618_H37Rv03_9_18.fasta
>sp|P9WLL9|Y2067_MYCTU Uncharacterized protein Rv2067c OS=Mycobacterium tuberculosis (strain ATCC 25618 / H37Rv) OX=83332 GN=Rv2067c PE=1 SV=1
MTDDHPRADI VSRQYHRWLY PHPIADLEAW TTANWEWFDP VHSHRILWPD REYRPDLDIL IAGCGTNQAA IFAFTNRAAK VVAIDISRPA LDHQQYLKDK
HGLANLELHL LPIEELATLG RDFDLVVSTG VLHHLADPRA GMKELAHCLR RDGVVAAMLY GKYGRIGVEL LGSVFRDLGL GQDDASIKLA KEAISLLPTY
HPLRNYLTKA RDLLSDSALV DTFLHGRQRS YTVEECVDLV TSAGLVFQGW FHKAPYYPHD FFVPNSEFYA AVNTLPEVKA WSVMERLETL NATHLFMACR
RDRPKEQYTI DFSTVAALDY VPLMRTRCGV SGTDMFWPGW RMAPSPAQLA FLQQVDGRRT IREIAGCVAR TGEPSGGSLA DLEEFGRKLF QSLWRLDFVA
VALPASG
Score ΔScore Ions
81-98 VVAIDISRPALDHQQYLK 2.43 0.47 22/68
152-162 DGVVAAMLYGK 2.40 0.46 16/20
166-176 IGVELLGSVFR 3.40 0.518 17/20
177-188 DLGLGQDDASIK 2.16 0.45 14/22
192-204 EAISLLPTYHPLR 2.97 0.392 15/24
212-227 DLLSDSALVDTFLHGR 4.28 0.669 21/30
254-279 APYYPHDFFVPNSEFYAAVNTLPEVK 2.37 0.416 25/100
306-325 EQYTIDFSTVAALDYVPLMR 2.32 0.55 14/38
342-358 MAPSPAQLAFLQQVDGR 4.53 0.745 23/32
371-387 TGEPSGGSLADLEEFGR 3.46 0.602 19/32
e
HEK: Rv2067c
THP1
WtMtb
Rv2067c
f
DAPI
Rv2067c
FITC
DIC
MERGE
ΔRv2067c
Rv2067c:OE
Zoomed and rotated
g
DAPI
Rv2067c
DIC
MERGE
M.smeg: FLAG
M.smeg: Rv2067c-FLAG
(Zoomed)
h
kDa
pcDNA SFB
pcDNA: 2067cSFB
pcDNA SFB
pcDNA: 2067cSFB
pcDNA SFB
pcDNA: 2067cSFB
40
Rv2067c
10
H3
50
Tubulin
CE
NE
Chromatin
i
FLAG
DAPI
MERGE
pcDNA FLAG
pcDNA:Rv2067c SFB
pcDNA:Rv2067c Δ30SFB
pcDNA:Rv2067c Δ55SFB
j
pcDNA SFB
pcDNA: 2067 Δ30SFB
pcDNA SFB
pcDNA: 2067 Δ30SFB
kDa
FLAG
40
H3
10
Tubulin
50
CE
NE
Full Length
Del 30
RK-X6-R
Del 55
RRTIR-X24-RK

Supplementary Figure 3: Secretion and localization of Rv2067c. a, Western blot for detection of Rv2067c in the culture filtrate (CF) of Wt*Mtb*. Cell lysate (CL) and culture filtrate (CF) were probed with Rv2067c antibody. GroEL1 (a non-secretory protein) antibody was used to examine cell lysis and CFP10 (a secretory protein) was kept as positive control. **b,** Western blot depicts secretion of Rv2067c in THP1 macrophages infected with Wt*Mtb*. H3 was kept as loading control. **c,** Western blot for secretion of Rv2067c from *M.smeg*. Cell lysate (CL) and culture filtrate (CF) of *M.smeg*:Rv2067c-FLAG was probed with FLAG and GroEL1 antibody. **d,** Detection of Rv2067c by mass spectrometry in *M.smeg*:Rv2067c-FLAG culture filtrate. Fragments detected in mass spectrometry analysis are underlined and highlighted in green. **e,** Blot depicts specificity of Rv2067c antibody for cell lysates of HEK293T transfected with pcDNA: Rv2067cSFB, uninfected THP1 macrophages and Wt*Mtb*. **f,** Immunofluorescence analysis of THP1 macrophages infected with FITC labelled ΔRv2067c and Rv2067c:OE (green). Signal for secretory Rv2067c was detected with Rv2067c antibody + Alexa 568 conjugated secondary antibody (red). White and yellow arrows show presence of Rv2067c in the cytoplasm and nucleus respectively. Scale bar = 5mm. **g,** Confocal images of THP1 macrophages infected with *M.smeg*:FLAG and *M.smeg*:Rv2067c-FLAG. Signal for Rv2067c was detected by immunostaining with FLAG antibody + Alexa 488 conjugated secondary antibody (green). Inset shows zoomed in view of the region marked by red box. Scale bar = 5mm. **h,** Subcellular fractions of HEK293T transfected with pcDNA: Rv2067cSFB. Blot was probed with Rv2067c antibody, H3 and tubulin antibody was used as control for chromatin and cytoplasmic extract (CE), respectively. HEK cells transfected with pcDNA:SFB were kept as control. **i,** Nuclear localization signal for Rv2067c was detected by immunostaining with FLAG antibody + Alexa 568 conjugated secondary antibody (red). Topmost panel: pcDNA SFB control, second panel: localization of Rv2067c full length (Rv2067c SFB); last two panels: Rv2067c deletion mutants Rv2067cΔ30SFB and Rv2067cΔ55SFB as indicated. Scale bar = 5mm. **g**, **h** and **i**, Nuclei was stained with DAPI (blue). **j,** Subcellular fractions of HEK cells transfected with pcDNA SFB and pcDNA: Rv2067cΔ30SFB were probed with indicated antibodies. Tubulin and H3 antibodies were used as controls for cytoplasmic and nuclear fractions (CE and NE), respectively. SFB: S-protein, FLAG, streptavidin-binding peptide; *M.smeg*: *M. smegmatis*. Source data are provided as a Source Data file.

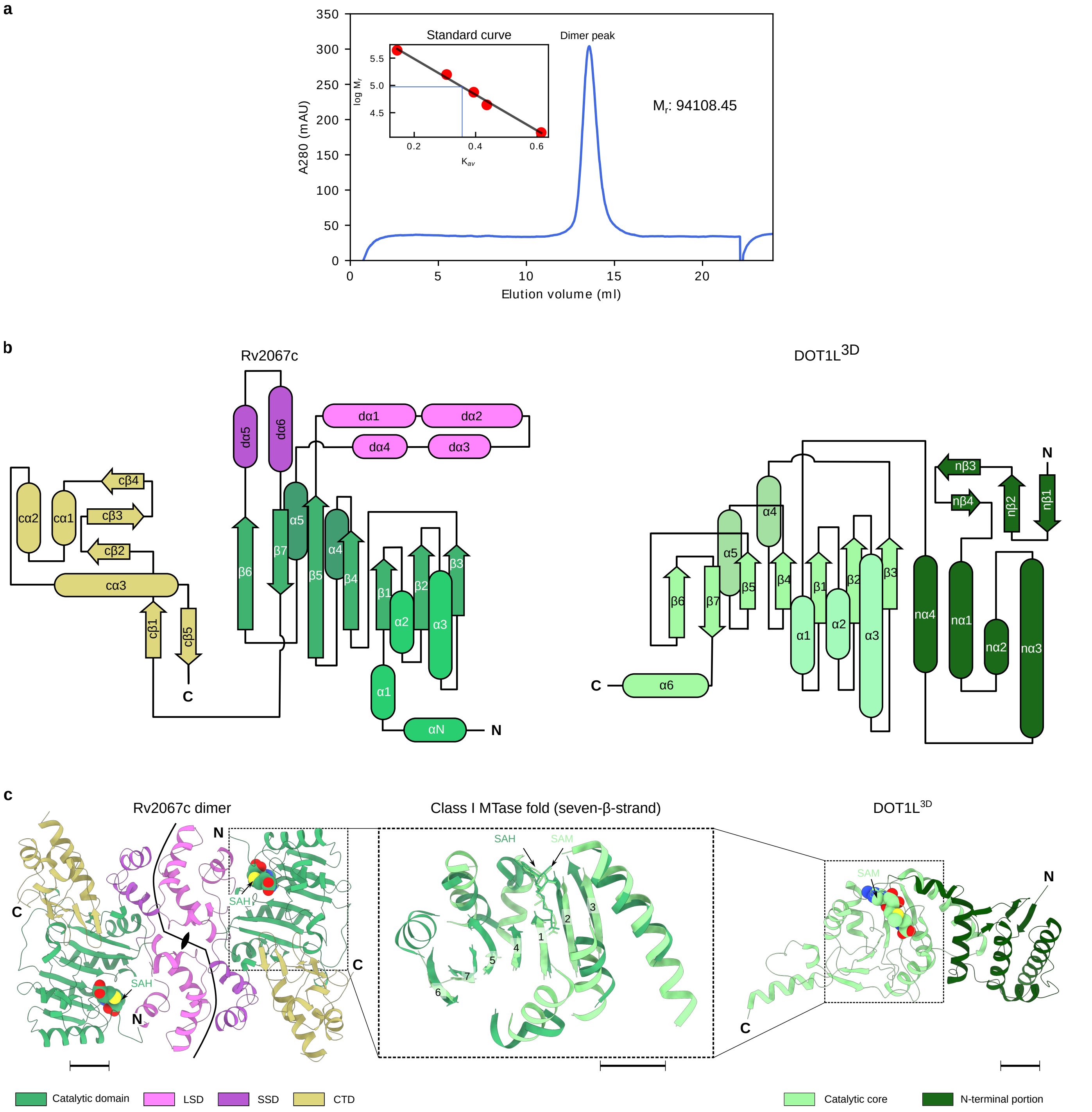

Supplementary Figure 4: Structural comparison between Rv2067c and DOT1L. **a,** Analytical gel filtration chromatogram showing Rv2067c elution profile. Rv2067c elutes as a dimer with observed M_r 94,108.45 (calculated monomer M_r 45,930). mAU: milli-absorbance units. **b,** Topology diagram of Rv2067c (left panel) and DOT1L^{3D} (right panel, PDB: 1NW3 [https://doi.org/10.2210/pdb1NW3/pdb]). **c,** Superposition of catalytic cores of Rv2067c and DOT1L (PDB: 1NW3). Both share a class I MTase fold (7BS: seven-β-strand). The β-strands are numbered through 1 to 7. The N- and C-termini are labeled as N and C, respectively. LSD: Large subdomain of dimerization domain; SSD: Small subdomain of dimerization domain; CTD: C-terminal domain; SAH: S-adenosyl-L-homocysteine; SAM: S-adenosyl-L-methionine. Source data for the elution profile of analytical gel filtration chromatography and plotting script are provided as Source Data file and Supplementary Software File 1, respectively.

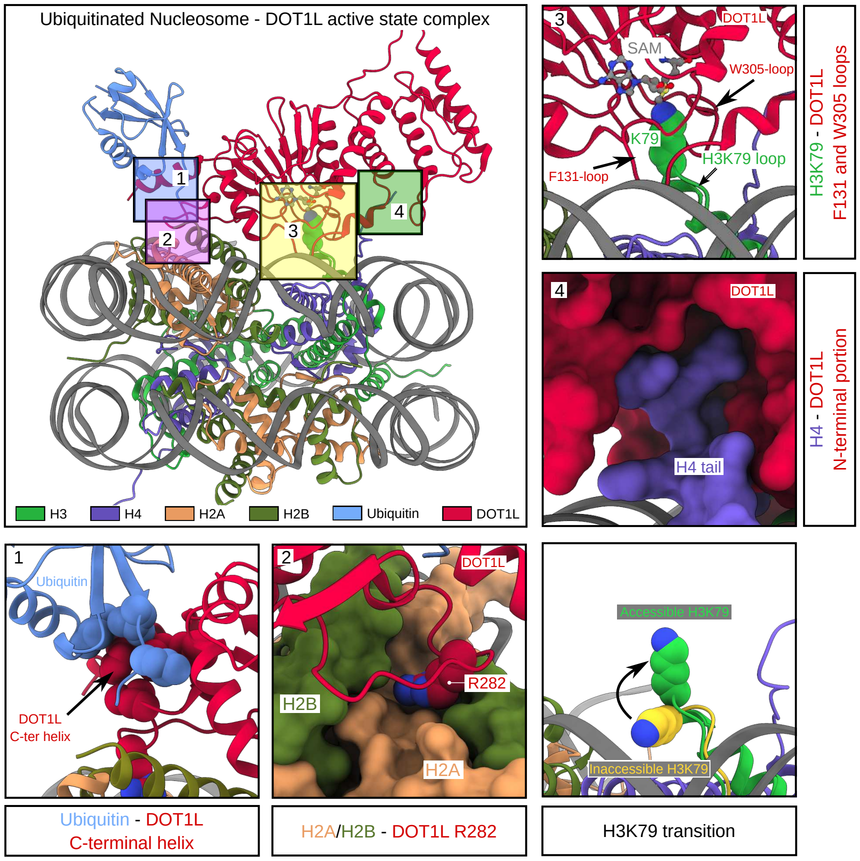

Supplementary Figure 5: Interactions between DOT1L and ubiquitinated nucleosome (PDB: 6NJ9 [https://doi.org/10.2210/pdb6NJ9/pdb]). DOT1L binds across the nucleosome surface and interacts with different regions of the nucleosome viz. (1) Ubiquitin, (2) H2A-H2B acidic path, (3) H3K79 loop, and (4) H4 tail. During methylation H3K79 loop orients to place the H3K79 from its inaccessible to accessible conformation (bottom right panel). SAM: S-adenosyl-L-methionine.

Structural elements of DOT1L that interact with UbNuc and their equivalences in Rv2067c:

DOT1L	Rv2067c
**Ubiquitin interaction**	
C-terminal helix (aa 319-330)	Leads to CTD
**H2A-H2B acidic patch interaction**	
R282 (β5-β6 loop)	LSD
**H4 tail interaction**	
Acidic groove of N-terminal portion	No N-terminal protion
**H3K79 loop interactoin**	
W305 loop (β6-β7 loop)	SSD
F131 loop (connects N-ter portion and catalytic core)	N-terminus (covers active site)

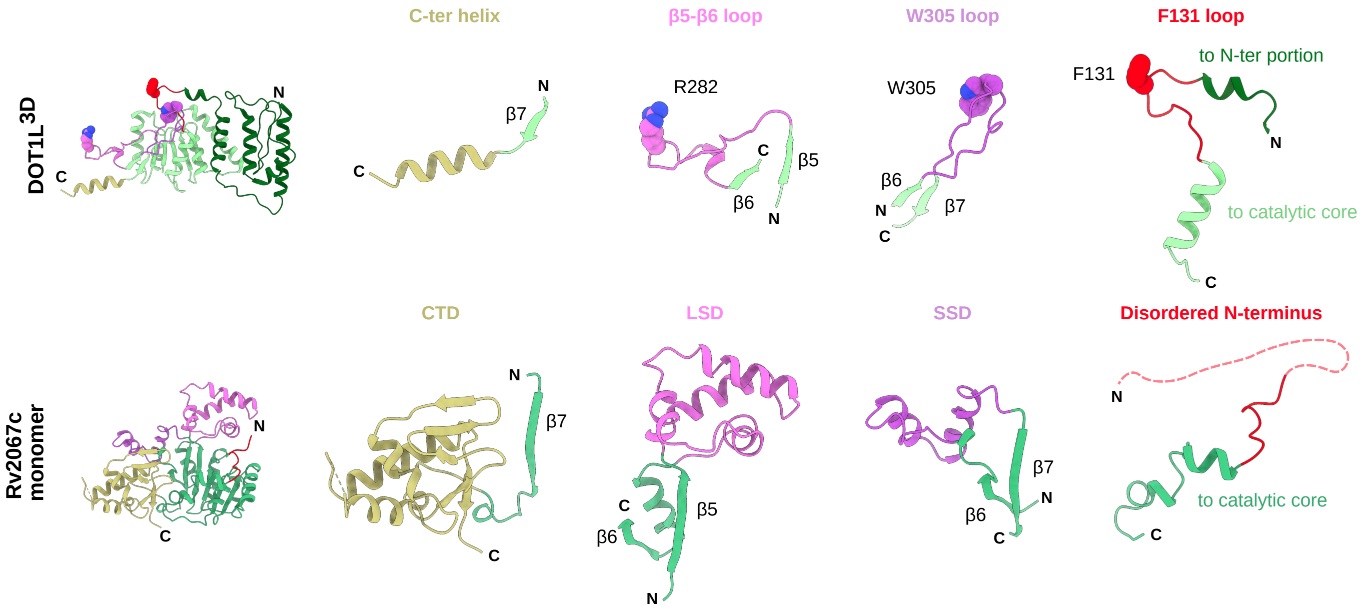

Supplementary Figure 6: Structural elements of DOT1L essential for nucleosomal H3K79 methylation and their structural equivalences in Rv2067c. The N- and C-termini are labeled as N and C, respectively. UbNuc: Ubiquitinated nucleosome; CTD: C-terminal domain; LSD: Large subdomain of dimerization domain; SSD: Small subdomain of dimerization domain.

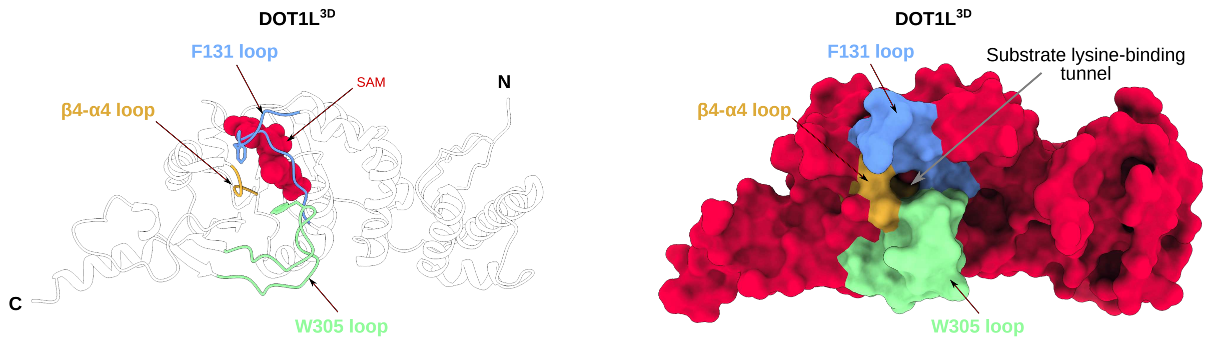

Supplementary Figure 7: The active site of DOT1L (PDB: 6NJ9 [https://doi.org/10.2210/pdb6NJ9/pdb]). The active site is a tunnel formed by three loops viz. F131, β4-α4 and W305. One end of the tunnel is capped by methyl group of SAM and the other end is open for substrate lysine (H3K79) entry. The DOT1L is represented as a cartoon (left) and surface (right). The N- and C-termini are labeled as N and C, respectively. SAM: S-adenosyl-L-methionine.

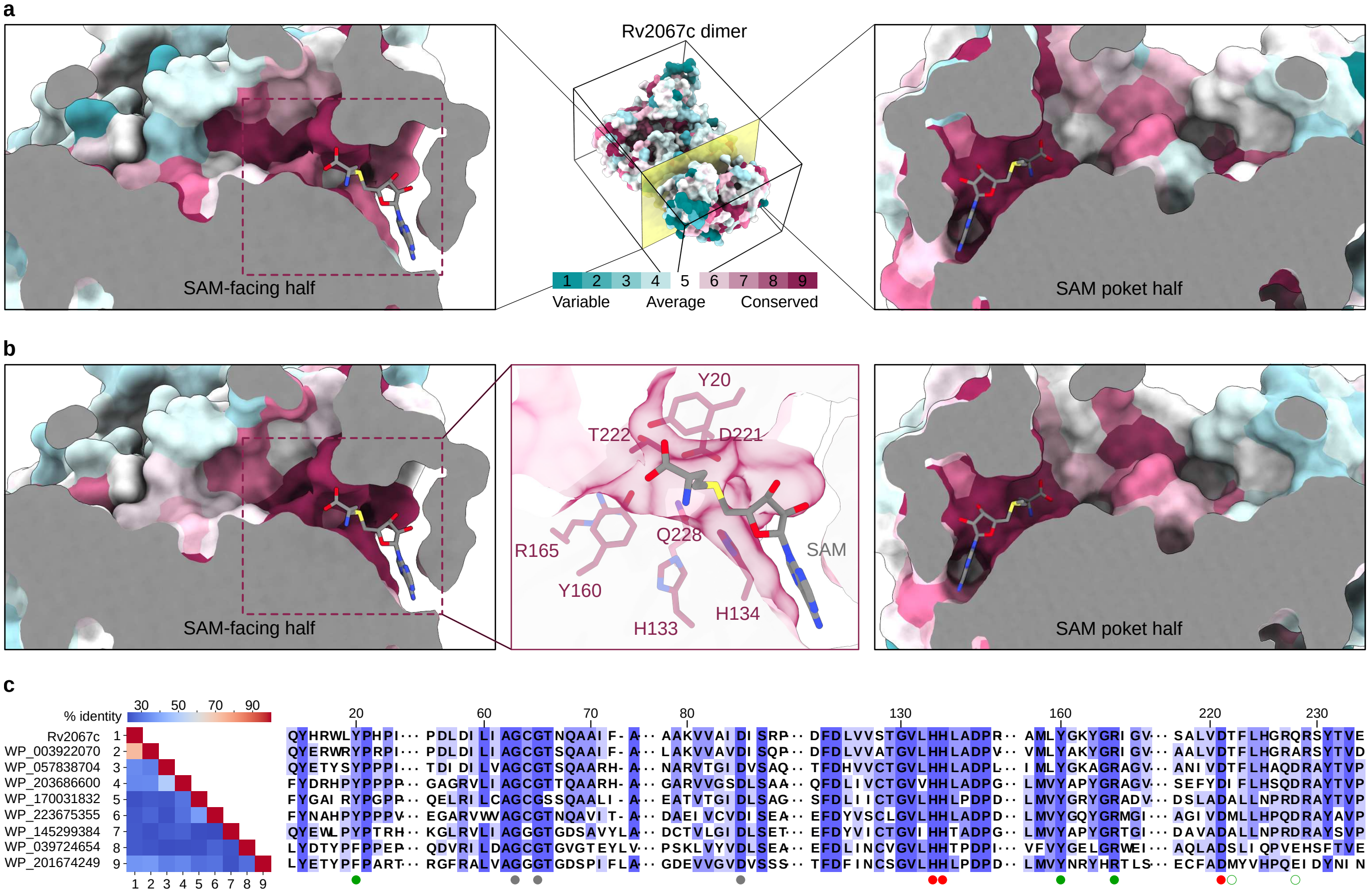

Supplementary Figure 8: Residue conservation in the substrate-binding trough of Rv2067c. a and **b,** Sequence conservation for set A (only mycobacterial species, (**a**)) and set B (all species, (**b**)) was mapped onto the Rv2067c structure using ConSurf (Supplementary Methods). Cross sections along the substrate-binding trough: SAM pocket half (right panel) and SAM-facing half (left panel). The region encompassing the SAM is highly conserved compared to the rest of the trough. The putative active site residues (Y20, Y160, R165, D221, T222 and Q228) and putative catalytic residues (H133, H134 and D221) form a conserved patch opposite to the methyl group of SAM (middle panel of **b**). **c,** Multiple sequence alignment of representative sequences from set B is presented. Pairwise sequence identity is shown as matrix. The highly conserved residues marked with filled circles: green for active site residues (possible substrate lysine binding), grey for SAM-binding (63-GxG-65 motif and D85) and red for putative catalytic residues. The open green circles represent the residues part of the active site and are less conserved or not conserved. SAM: S-adenosyl-L-methionine.

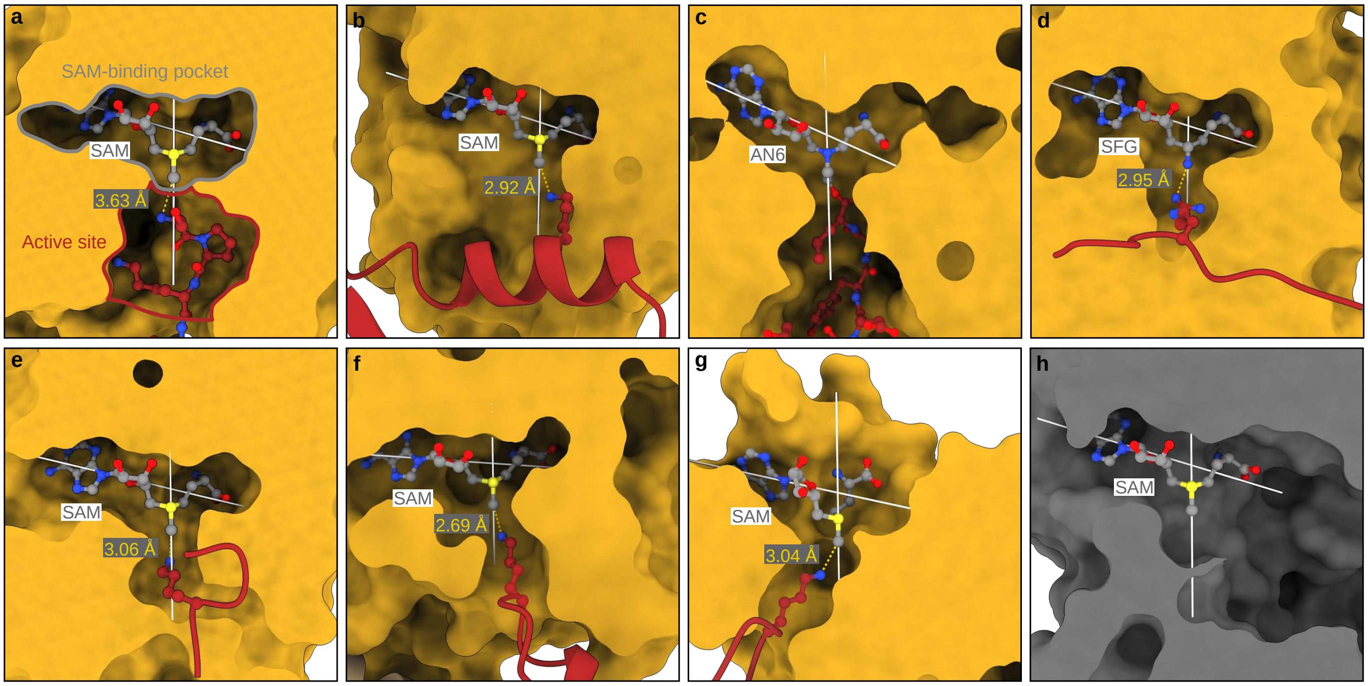

Supplementary Figure 9: Mode of methyl acceptor residue (substrate) binding to the active site of protein methyltransferases (MTases). The active site lies nearly perpendicular to the SAM-binding pocket. The oblique white line is the first principle component (PC1) of SAM and passes through the SAM-binding pocket. The vertical white line that is nearly orthogonal to PC1 that passes through the scissile bond of SAM (the bond between sulfur and methyl carbon atoms of SAM) and aligns with the active site where methyl acceptor residue binds. The MTase-substrate complexes are presented. **a,** N-terminal methyltransferase 1 (NTMT1) from human (PDB: 5E1B [https://doi.org/10.2210/pdb5E1B/pdb]), **b,** N-terminal/N-epsilon methyltransferase (PrmA, ribosomal protein L11 methyltransferase) from *Thermus thermophilus* (PDB: 2NXN [https://doi.org/10.2210/pdb2NXN/pdb] and 3EGV [https://doi.org/10.2210/pdb3EGV/pdb], combined), **c,** Leucine carboxy methyltransferase (LCMT) from human (PDB: 3P71 [https://doi.org/10.2210/pdb3P71/pdb]), **d,** Protein arginine methyltransferase (PRMT) from human (PDB: 5DX0 [https://doi.org/10.2210/pdb5DX0/pdb]), **e,** Lysine methyltransferase 9 (KMT9, histone H4 lysine 12 methyltransferase) from human (PDB: 6H1E [https://doi.org/10.2210/pdb6H1E/pdb]), **f,** DOT1L (Disruptor of telomeric silencing 1-like histone lysine methyltransferase) form human (PDB: 6NJ9 [https://doi.org/10.2210/pdb6NJ9/pdb]), **g,** SET (Su(var)3-9, Enhancer-of-zeste and Trithorax) domain methyltransferase, SET7/9, from human (PDB: 2F69 [https://doi.org/10.2210/pdb2F69/pdb]), and **h,** Rv2067c from *M. tuberculosis* H37Rv (current study). The SAM-binding pocket and the active site region are outlined for (**a**) in grey and crimson colors, respectively. The MTases are shown in goldenrod color, except Rv2067c (dim grey). The methly acceptor substrates are shown in crimson color. The distance between the SAM methyl carbon (nitrogen in the case of SFG) and the methyl acceptor atom is shown as yellow dashed line with distance value. SAM: S-adenosyl-L-methionine; AN6: 5′-[(3S)-3-amino-3-carboxypropyl](ethyl)amino-5′-deoxyadenosine; SFG: Sinefungin.

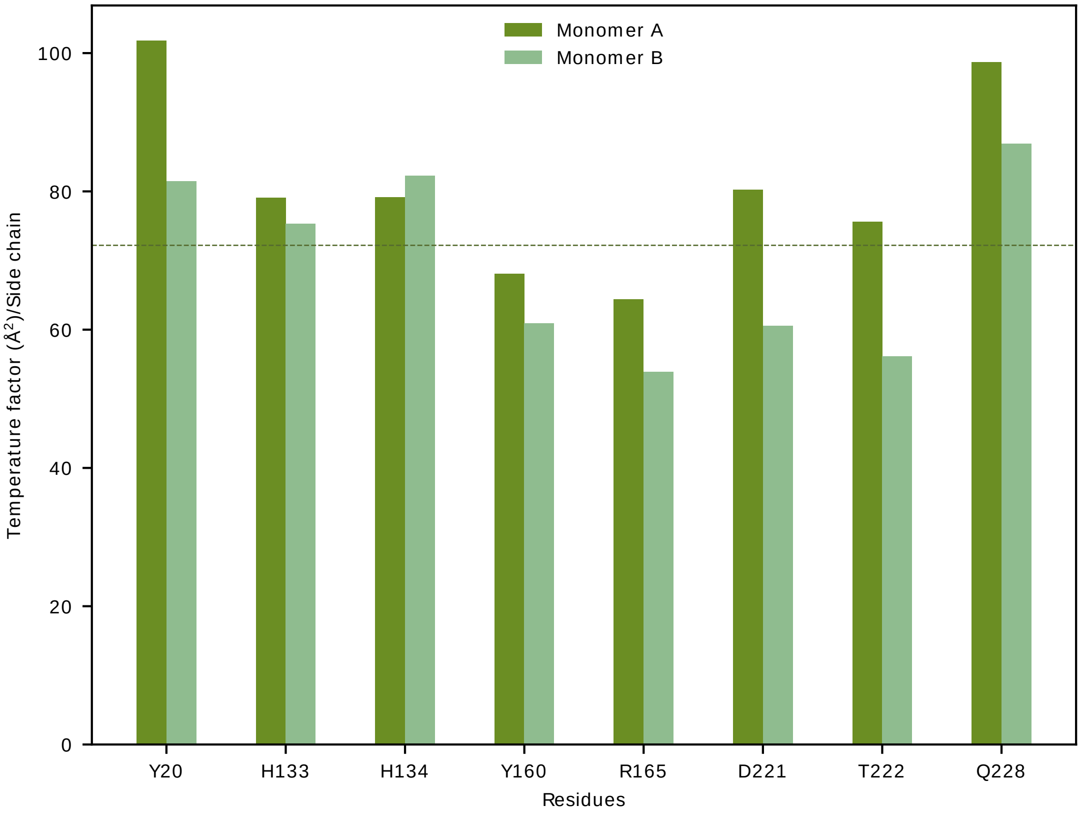

Supplementary Figure 10: Side chain temperature factors of active stie residues of Rv2067c. Residues Y20 and Q228 have relatively high temperature factors, especially for monomer A (chain A). The average side chain temperature factor is shown as a horizonal dashed line. Source data and plotting script are provided as Supplementary Data 3 and Supplementary Software File 2, respectively.

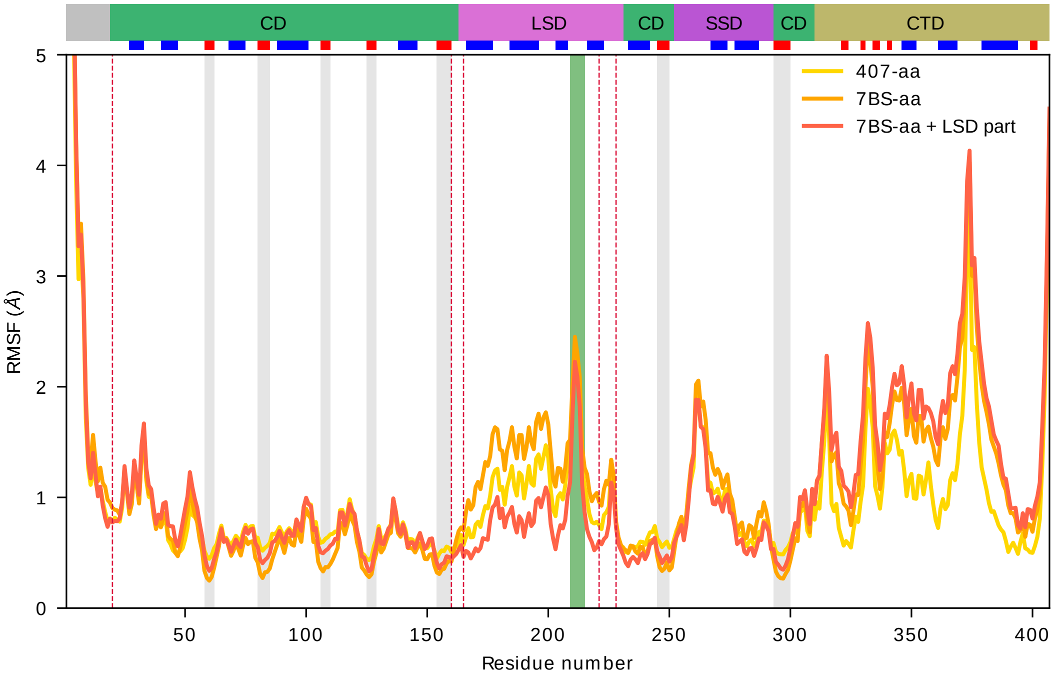

Supplementary Figure 11: The root mean square fluctuations (RMSF) of monomer A of Rv2067c. The RMSF was calculated after aligning the trajectory to the crystal structure using three separate backbone atom groups viz. All residues (407-aa), only β-strands of the catalytic domain (7BS-aa) and 7BS-aa + part of the LSD (see Supplementary Methods). Light grey vertical bars indicate the β-strands of the catalytic domain. Green vertical bar indicates the loop (209 –214 aa) of LSD near the putative active stie show relatively high RMSF. The vertical lines (red) mark the residues (Y20, Y160, R165, D221, T222 and Q228) at the occluded putative active site. The schematic of the domains' span and secondary structure elements of Rv2067c are shown at the top of the plot. 7BS: seven-β-strand; CD: Catalytic domain; LSD: Large subdomain of dimerization domain; SSD: Small subdomain of a dimerization domain; CTD: C-terminal domain; Secondary structure elements: α-helix (blue), β-strand (red). Source data and plotting script are provided as Source Data file and Supplementary Software File 3, respectively.

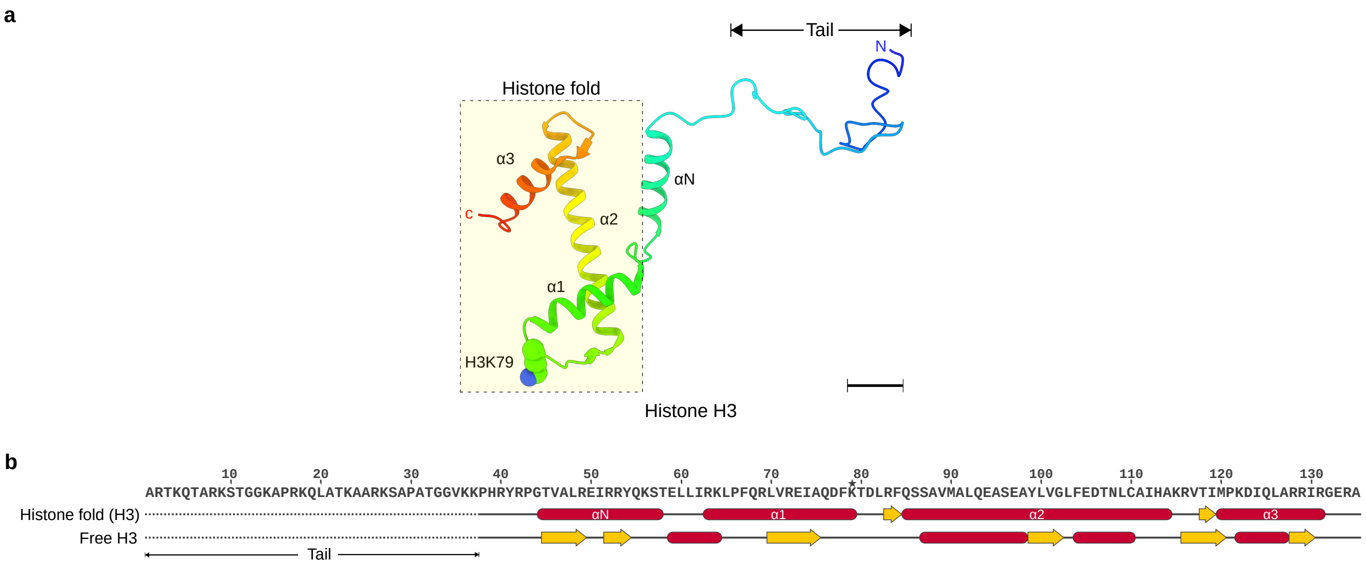

Supplementary Figure 12: Histone fold. a, The histone fold is shown for histone H3 (part of a nucleosome; chain A, PDB:1KX5 [https://doi.org/10.2210/pdb1KX5/pdb]). The three α-helices, α1, α2 and α3, constitute the histone fold. The N- and C-termini are labeled as N and C, respectively. **b,** The secondary structure elements of the histone fold and free H3. The secondary structure elements for free H3 were assigned based on synchrotron radiation circular dichroism experiments[10,11]. The composition of the secondary structure elements of free H3 are different from histone fold.

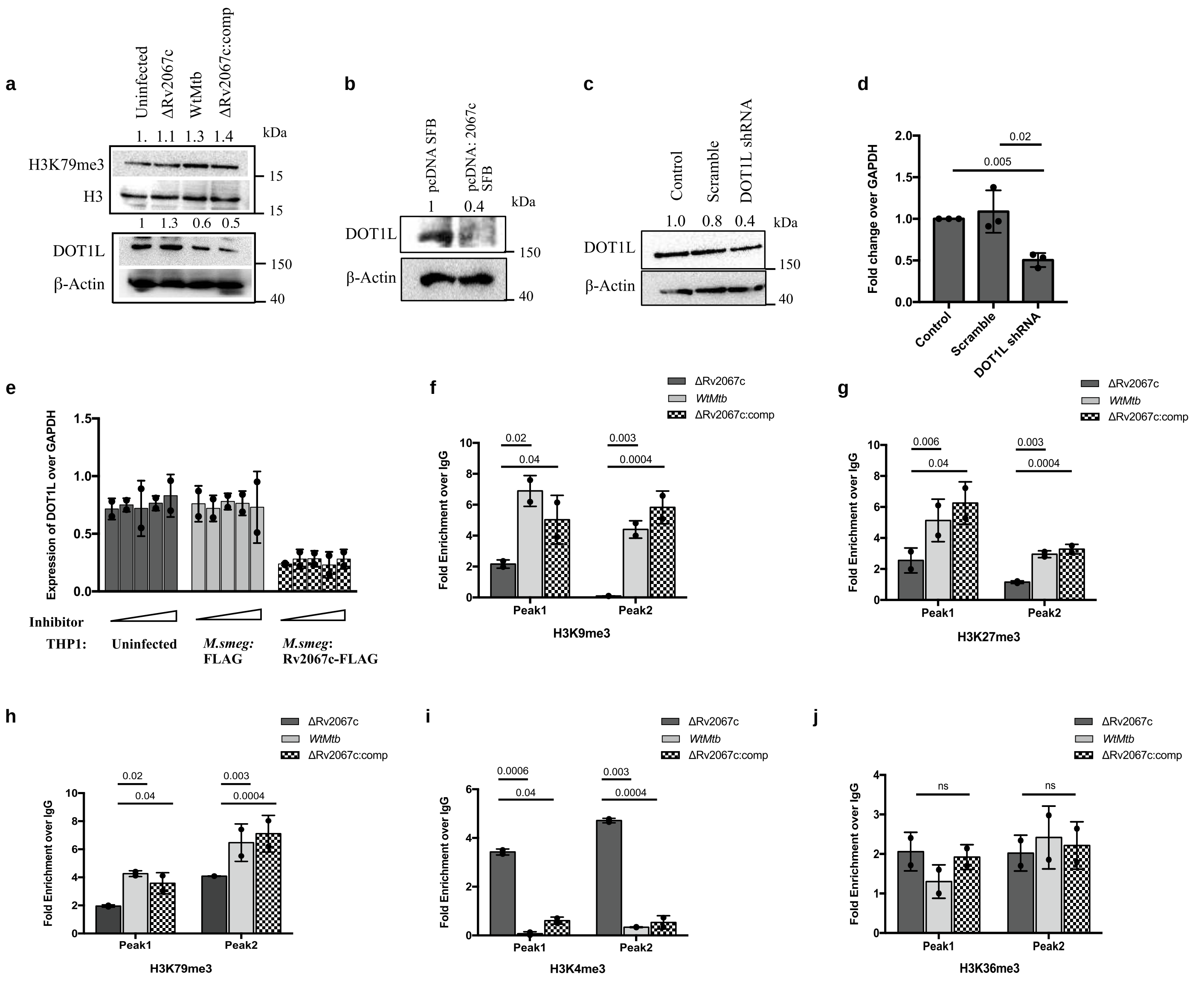

Supplementary Figure 13: Rv2067c modulates DOT1L expression. a, Immunoblots depict level of DOT1L and H3K79me3 in THP1 cell lysates 12 h.p.i with various *Mtb* strains. H3 and β-Actin were used as loading control. Lane1: uninfected THP1 macrophages; Lane 2, 3 and 4: THP1 macrophages infected with ΔRv2067c, Wt*Mtb* and ΔRv2067c:comp strains respectively. **b,** DOT1L level in cell lysates of HEK293T transfected with pcDNA: Rv2067cSFB and pcDNA SFB construct. β-Actin was kept as loading control. **c** and **d,** Expression of DOT1L in HEK293T cells after transfection with scramble (lane2) and DOT1L shRNA (lane3) by western analysis and qRT-PCR. Control represents untransfected cells. β-Actin was used as loading control. For qRT-PCR, Ct values were normalised against GAPDH. n = 3 independent experiments. **e,** Expression of DOT1L in uninfected THP1(left panel), THP1 infected with *M.smeg*:FLAG (middle panel) and *M.smeg*:Rv2067c-FLAG (right panel) post treatment with EPZ004777. Inhibitor was added in increasing concentration of 0, 1.25, 2.5, 5 and 10 μM for each set. Ct values were normalised against GAPDH. n = 2 independent infections. **f-j,** Fold enrichment of H3K9me3, H3K27me3, H3K79me3, H3K4me3 and H3K36me3 marks on peak 1 and peak 2 on the DOT1L gene in THP1 macrophages infected with *Mtb* strains. Bar pattern represents infection with following strains: Dark grey: ΔRv2067c, Light grey: Wt*Mtb*, checker: ΔRv2067c:comp. Data is represented as fold enrichment over IgG control. n = 2 independent infections. For all bar graphs - Data is plotted as mean and error bars represent SD. P-values depicted on the graph were calculated using unpaired two tailed Student's t-test; ns-non significant. Values above the blot represent quantitation (arbitary units). SFB: S-protein, FLAG, streptavidin-binding peptide; Comp: Complemented; *M.smeg*: *M. smegmatis*. Source data are provided as a Source Data file.

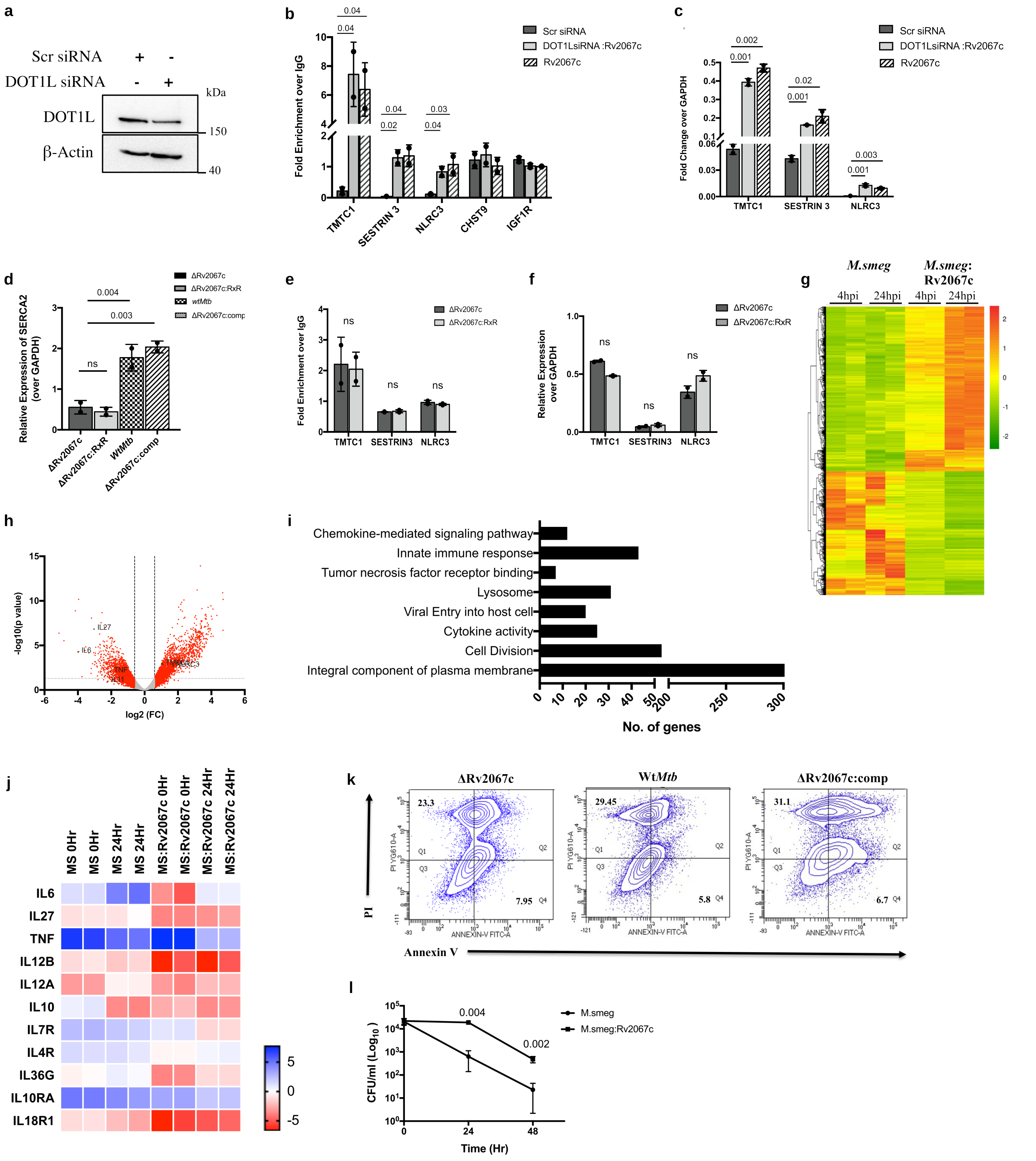

Supplementary Figure 14: Downstream events consequent to H3K79 methylation by Rv2067c. a, Immunoblots showing DOT1L levels in HEK293T treated with 20 nM of scramble and DOT1L siRNA; loading control - β-Actin. **b,** Bar graph depicts fold enrichment of H3K79me3 added by Rv2067c on indicated gene loci in HEK-scr:pcDNA (dark grey), HEK-DOT1L KD:Rv2067c (light grey) and HEK:Rv2067c (stripe) cells. Data is shown as fold enrichment over IgG control. **c,** Graph shows fold change in expression of the indicated genes in HEK-scr: pcDNA (dark grey), HEK-DOT1L KD:Rv2067c (light grey) and HEK:Rv2067c (stripe) cells. **d,** Graph shows relative expression of SERCA2 in THP1 infected with ΔRv2067c (dark grey), ΔRv2067c:RxR (light grey) Wt*Mtb* (checker) and ΔRv2067c:comp (stripe)

with respect to uninfected THP1. Ct values were normalized against GAPDH. **e,** Fold enrichment of H3K79me3 mark in THP1 macrophages infected with ΔRv2067c (dark grey) and ΔRv2067c:RxR (light grey). Data is shown as fold enrichment over IgG control. **f,** Relative expression of TMTC1, NLRC3 and SESTRIN3 in THP1 infected with ΔRv2067c (dark grey) and ΔRv2067c:RxR (light grey) with respect to uninfected THP1. Ct values were normalized against GAPDH. (**b-f**) All ChIP and qRT data is representative of two independent experiments (n = 2). Data is plotted as mean and error bars represent SD. P-values depicted on the graph were calculated using unpaired two tailed Student's t-test; ns-non significant. **g,** Heatmap shows relative expression profile of differentially expressed genes across the samples p.i with *M.smeg* and *M.smeg*:Rv2067c strains in macrophages. **h,** Volcano plot shows differential expression profile of genes after 24hr of THP1 infection with *M.smeg*:Rv2067c. Red dots beyond black (vertical) and grey dotted lines (horizontal) indicate genes with fold change $\geq$1.5 and P-value $\leq$0.05. Grey dots indicate genes with fold change <1.5. Black dots show representative upregulated and downregulated genes. **i,** Bar graph shows functional annotation clusters of downregulated genes using DAVID (enrichment score >5). **j,** Heatmap depicts relative expression levels for cytokines post infection with *M.smeg* and *M.smeg*:Rv2067c strains. $\log_2$RPKM values were plotted. **k,** Representative scatter plots of PI (y-axis) versus annexin V (x-axis) for macrophages infected with ΔRv2067c, Wt*Mtb* and ΔRv2067c:comp. Percentage of apoptotic and necrotic cells are indicated in Q4 and Q1 respectively. **l,** CFU analysis of THP1 macrophages infected with *M.smeg* (circle) and *M.smeg*:Rv2067c-FLAG (square). Colony forming units were counted after 4, 24 and 48 hr of infection. n = 3 independent infection. Data is plotted as mean and error bars represent SD. P-values depicted on the graph were calculated using unpaired two tailed Student's t-test. SFB: S-protein, FLAG, streptavidin-binding peptide; Comp: Complemented; *M.smeg*: *M. smegmatis*; scr: scramble; KD: Knockdown; CFU: Colony forming units. Source data are provided as a Source Data file.

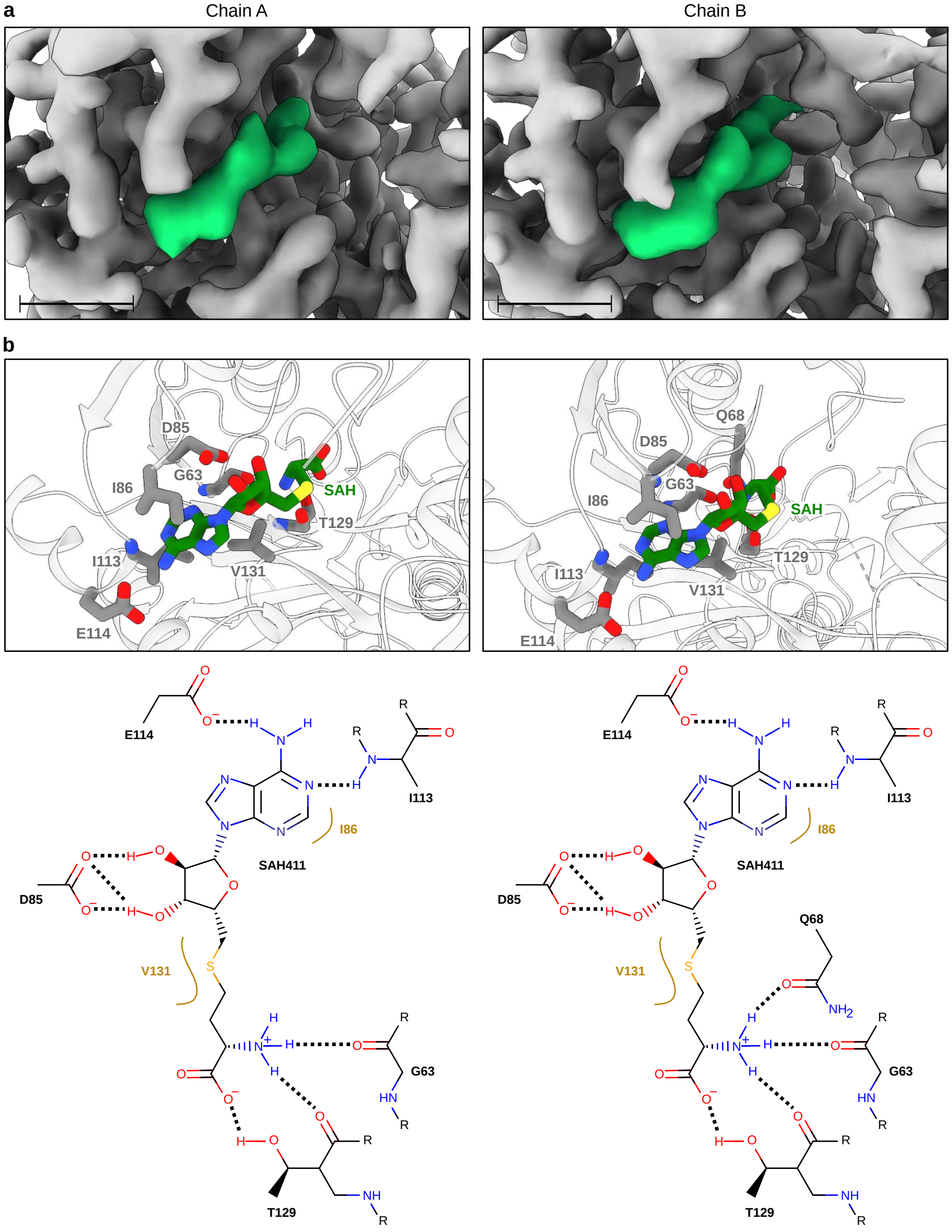

Supplementary Figure 15: **a,** The electron density map (2F_o - F_c at 1.5 σ, grey) is shown for protein (Rv2067c) . During model building, an unmodeled density (F_o - F_c map at 3.0 σ, green) at the SAM/SAH binding pocket was observed. The density was modeled as SAH. **b,** The interactions between Rv2067c and SAH are shown. SAM: S-adenosyl-L-methionine; SAH: S-adenosyl-L-homocysteine.

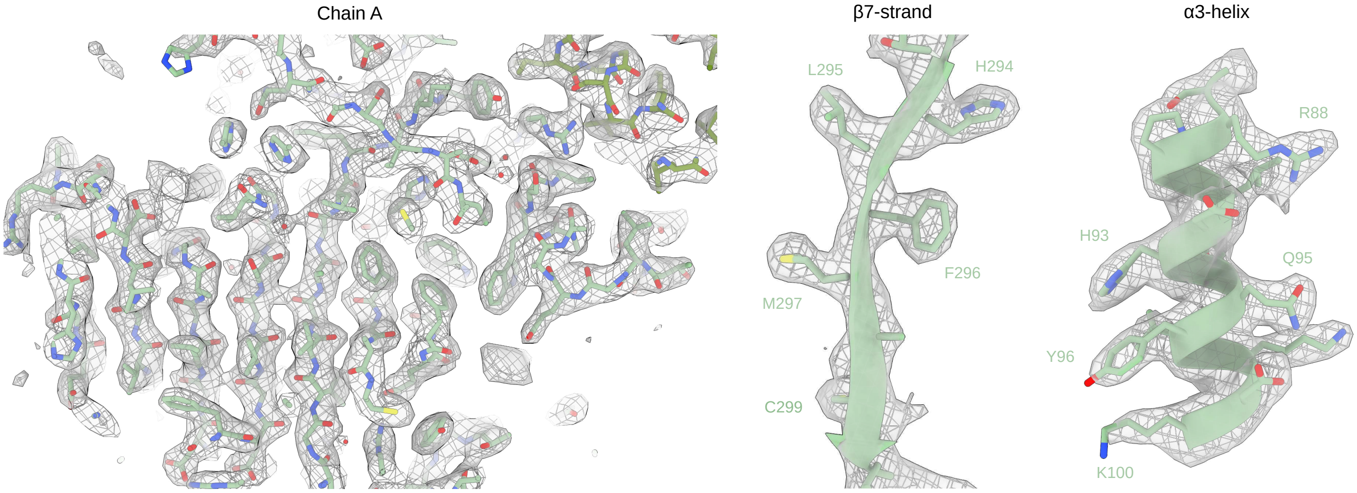

Supplementary Figure 16: Fourier difference map ($2F_o$ - F_c, at 1.0 σ) showing the representative electron density of Rv2067c crystal structure.

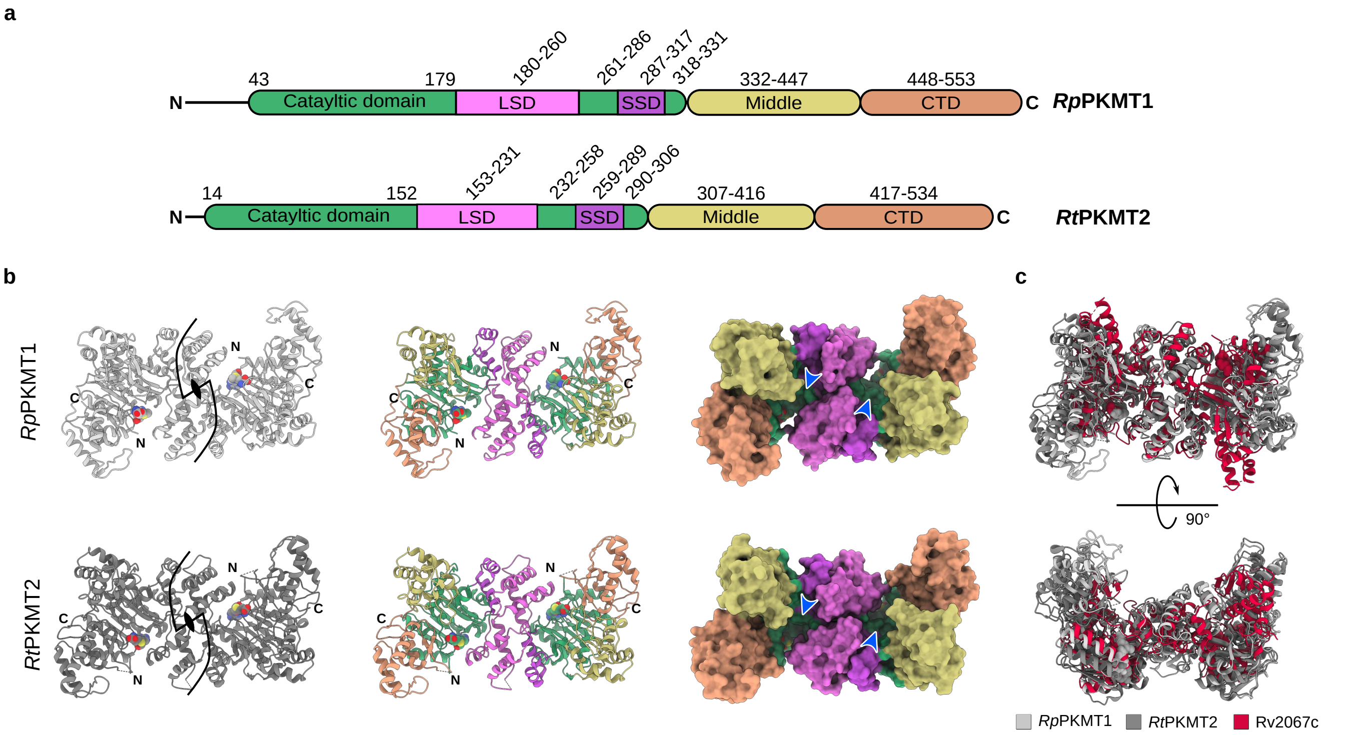

Supplementary Figure 17: Structures of rickettsial protein lysine MTases (PKMTs) and their comparison to Rv2067c. a, Schematic of *Rickettsia prowazekii* PKMT1 (*Rp*PKMT1) and *Rickettsia typhi* PKMT2 (*Rt*PKMT2) domain organization: catalytic domain, dimerization domain (LSD + SSD; LSD: Large subdomain of dimerization domain, SSD: Small subdomain of dimerization domain), Middle (middle domain) and CTD (C-terminal domain). **b,** Structures of *Rp*PKMT1 (PDB: 5DPD [https://doi.org/10.2210/pdb5DPD/pdb]) and *Rt*PKMT2 (PDB: 5DPL [https://doi.org/10.2210/pdb5DPL/pdb]). Domains are color coded according to (**a**). SAM or SAH are rendered as spheres. The dimer interface is marked with black line with a 2-fold axes (biconvex sign) passing perpendicular to the plane of the page. The substrate binding cleft is marked with blue arrow heads. **c,** Superposed structures of *Rp*PKMT1, *Rt*PKMT2 and Rv2067c. The CTD of Rv2067c is equivalent to the middle domain of PKMTs. The N- and C-termini are labeled as N and C, respectively. SAM: S-adenosyl-L-methionine; SAH: S-adenosyl-L-homocysteine;

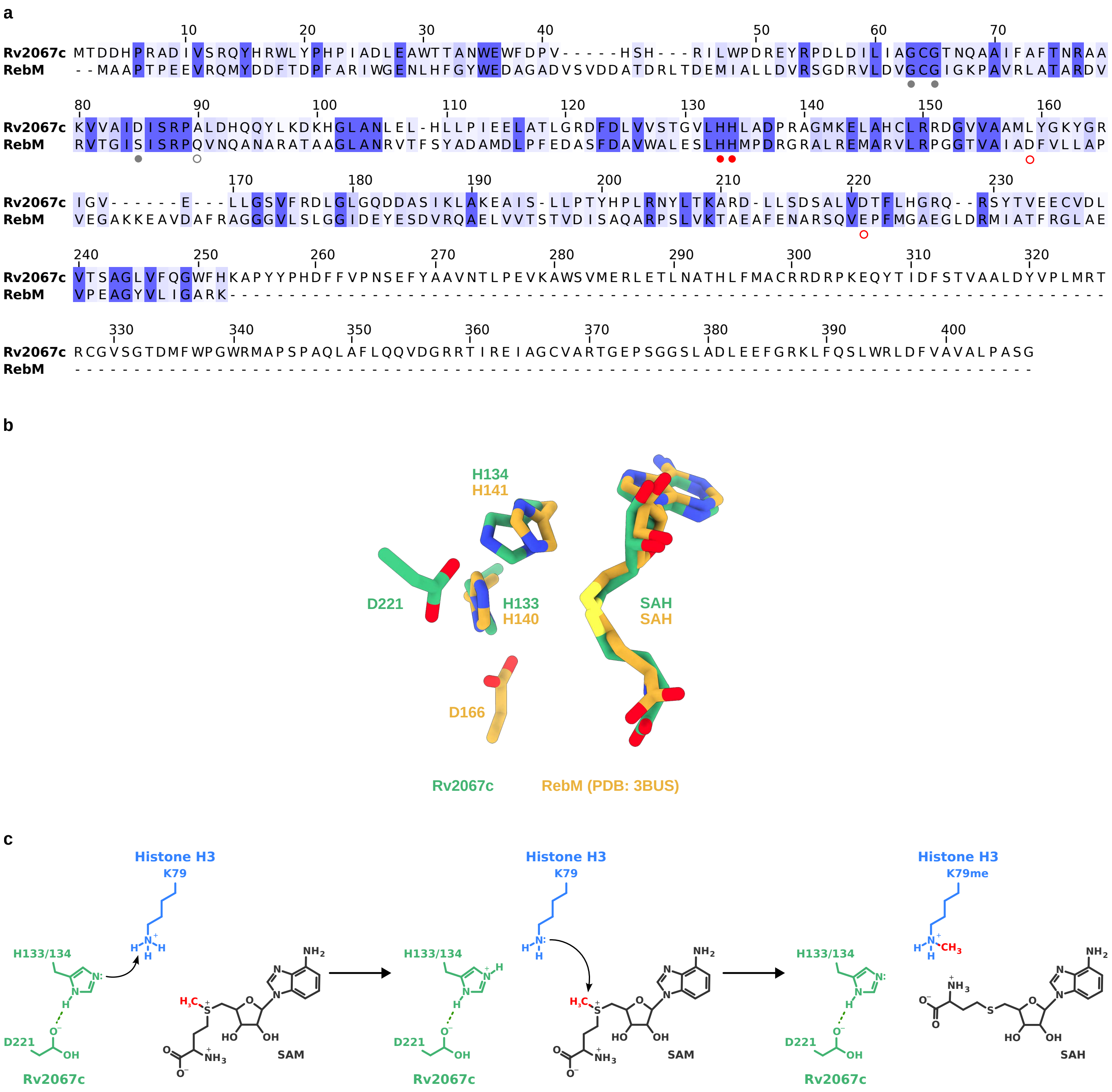

Supplementary Figure 18: Proposed catalytic mechanism of H3K79 methylation by Rv2067c. a, Sequence alignment between Rv2067c and a rebaccamycin sugar 4′-O-MTase, RebM. The important residues involved in SAM-binding and catalysis are marked with circles below the alignment. SAM-interacting residues are marked as filled grey circles for Rv2067c and RebM, and open grey circle is only of RebM; catalytic histidines are marked as filled red circles and aspartates (no positional conserveration) as open red circles. Residue numbering in the alignment is based on Rv2067c sequence. **b,** The structural alignment of Rv2067c and RebM (PDB: 3BUS [https://doi.org/10.2210/pdb3BUS/pdb]). For clarity, only the catalytic residues and SAH are shown as sticks. The catalytic histidines are well aligned, whereas the aspartates are at different positions but within the interacting distance with histidines. **c,** A proposed catalytic mechanism of H3K79 methylation by Rv2067c. Rv2067c follows a nucleophilic substitution (S_N2) mechanism. The general base, histidine (H133/H134) deprotonates H3K79 side chain. The neutral side chain amine with lone pair of electrons attacks the methyl carbon of SAM, leading to the transfer of methyl group from SAM to H3K79. SAM: S-adenosyl-L-methionine; SAH: S-adenosyl-L-homocysteine.

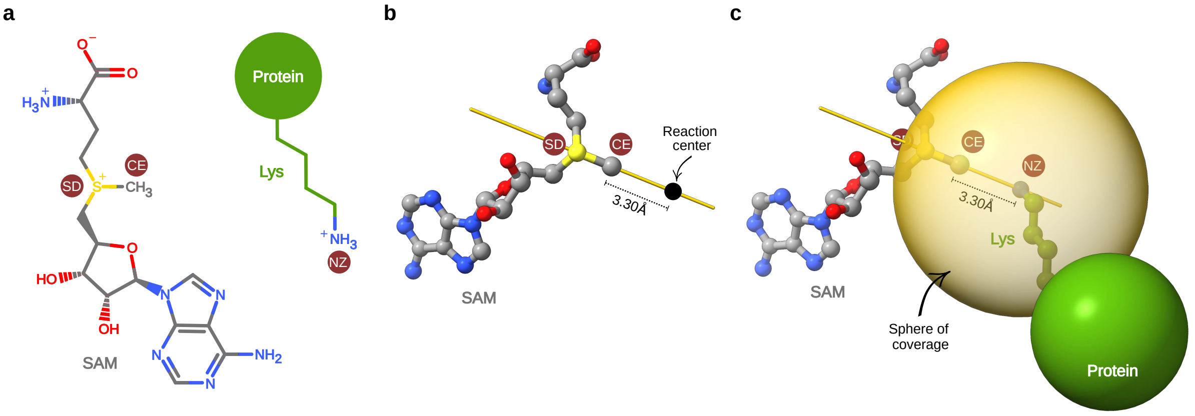

Supplementary Figure 19: The reaction center model for protein MTases used in rotation scan. a, Methyl donor SAM (left) and methyl acceptor substrate lysine (right). **b,** The reaction center is a point that lies on the axis that passes through the scissile bond of SAM (the bond between sulfur atom (SD^{SAM}, SD) and methyl carbon atom (CE^{SAM} , CE)), and at a distance from the CE^{SAM} atom equal to the sum of vdW radii of the CE^{SAM} and the methyl acceptor atom of lysine (NZ) of the substrate. **c,** In an imminent reaction, the methyl acceptor atom of the substrate (NZ of lysine) lies at the reaction center. In principle, the substrate can approach the reaction center from all orientations that constitute the sphere of coverage. SAM: S-adenosyl-L-methionine.

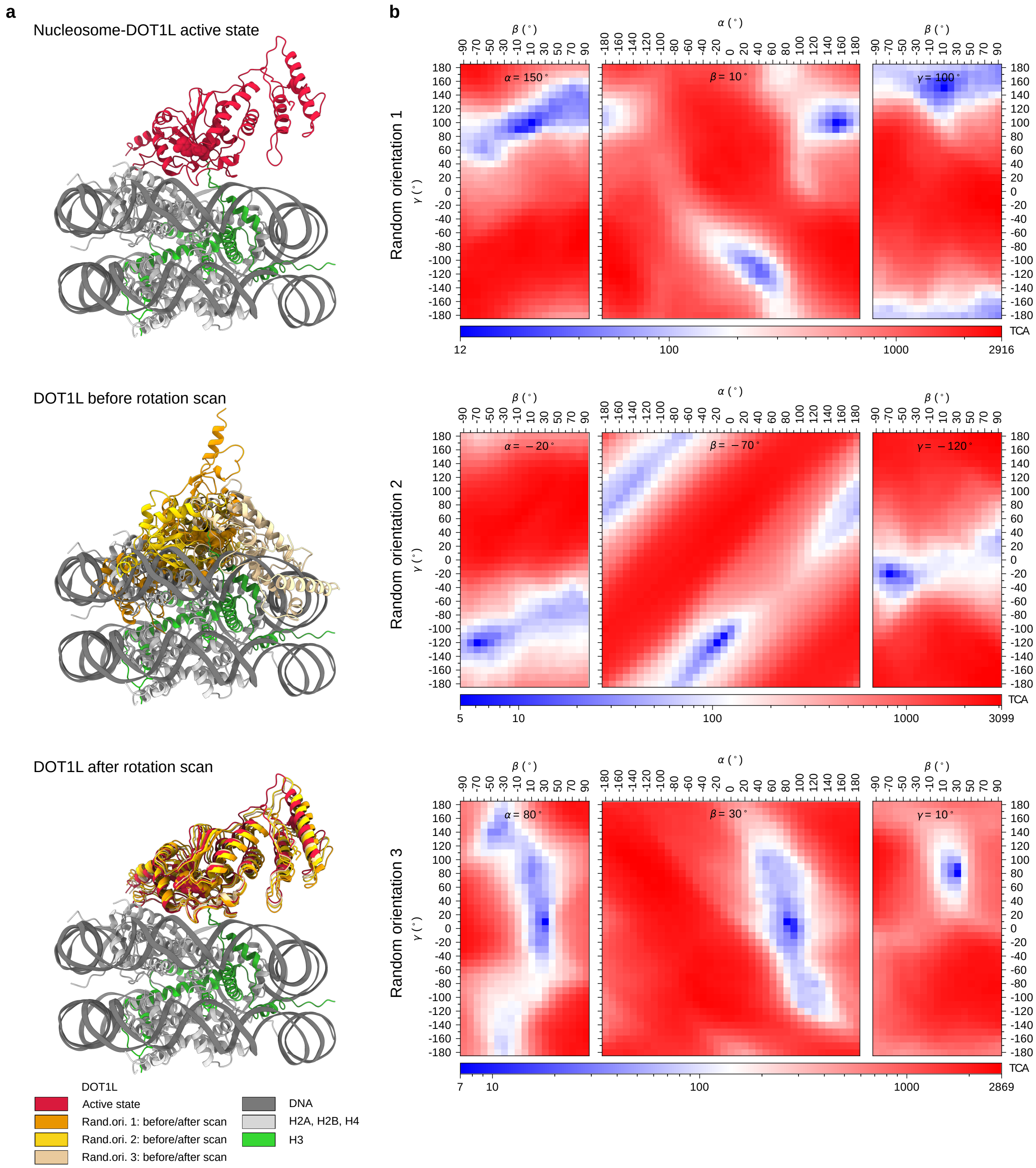

Supplementary Figure 20: Benchmarking the rotation scan on nucleosome-DOT1L complex (PDB: 6NJ9 [https://doi.org/10.2210/pdb6NJ9/pdb]). a, The active state nucleoosme-DOT1L complex was considered as the enzyme-substrate reaction complex (top). Three random orientations of DOT1L about the reaction center were generated (middle) to evaluate the rotation scan. Poses with minimum TCA generated by rotation scan from all three random orientations of DOT1L (bottom). All three poses with minimum TCA values align with active state DOT1L pose. **b,** Rotation scan TCA plots (see Fig. 4c) for all three random starting orientations of DOT1L in a nucleosome-DOT1L reaction complex. TCA: Total number of clashing atoms. Source data and plotting script for TCA plots are available at https://doi.org/10.5281/zenodo.8352903 and https://github.com/Venkat-Dadi/Rotation_Scan.

Supplementary References

1. Krissinel, E. Stock-based detection of protein oligomeric states in jsPISA. *Nucleic Acids Res.* **43**, W314–W319 (2015).

2. Egloff, M.-P., Benarroch, D., Selisko, B., Romette, J.-L. & Canard, B. An RNA cap (nucleoside-2'-O-)-methyltransferase in the flavivirus RNA polymerase NS5: crystal structure and functional characterization. *EMBO J* **21**, 2757–2768 (2002).

3. Min, J., Feng, Q., Li, Z., Zhang, Y. & Xu, R.-M. Structure of the catalytic domain of human DOT1L, a non-SET domain nucleosomal histone methyltransferase. *Cell* **112**, 711–723 (2003).

4. Coloma, J., Jain, R., Rajashankar, K. R., García-Sastre, A. & Aggarwal, A. K. Structures of NS5 methyltransferase from Zika virus. *Cell Rep.* **16**, 3097–3102 (2016).

5. Holm, L. DALI and the persistence of protein shape. *Protein Sci.* **29**, 128–140 (2020).

6. Abeykoon, A. H. *et al.* Structural insights into substrate recognition and catalysis in outer membrane protein B (OmpB) by protein-lysine methyltransferases from Rickettsia. *J Biol. Chem.* **291**, 19962–19974 (2016).

7. Abeykoon, A. *et al.* Multimethylation of Rickettsia OmpB catalyzed by lysine methyltransferases. *J Biol. Chem.* **289**, 7691–7701 (2014).

8. Singh, S. *et al.* Structure and mechanism of the rebeccamycin sugar 4'-O-methyltransferase RebM. *J Biol. Chem.* **283**, 22628–22636 (2008).

9. Campos, E. I. *et al.* The program for processing newly synthesized histones H3.1 and H4. *Nat. Struct. Mol. Biol.* **17**, 1343–1351 (2010).

10. Izumi, Y., Matsuo, K. & Namatame, H. Structural analysis of lysine-4 methylated histone H3 proteins using synchrotron radiation circular dichroism spectroscopy. *Chirality* **30**, 536–540 (2018).

11. Izumi, Y. *et al.* Circular dichroism spectroscopic study on structural alterations of histones induced by post-translational modifications in DNA damage responses: lysine-9 methylation of H3. *J Radiat. Res.* **59**, 108–115 (2018).

12. Worden, E. J., Hoffmann, N. A., Hicks, C. W. & Wolberger, C. Mechanism of cross-talk between H2B ubiquitination and H3 methylation by Dot1L. *Cell* **176**, 1490–1501 (2019).

13. Levenstein, M. E. & Kadonaga, J. T. Biochemical analysis of chromatin containing recombinant Drosophila core histones. *J Biol. Chem.* **277**, 8749–8754 (2002).

14. Luger, K., Rechsteiner, T. J. & Richmond, T. J. Expression and purification of recombinant histones and nucleosome reconstitution. *Methods Mol. Biol.* 1–16 (1999).

15. Lowary, P. & Widom, J. New DNA sequence rules for high affinity binding to histone octamer and sequence-directed nucleosome positioning. *J Mol. Biol.* **276**, 19–42 (1998).

16. Dyer, P. N. *et al.* Reconstitution of nucleosome core particles from recombinant histones and DNA. In *Meth. Enzymol.*, vol. 375, 23–44 (Elsevier, 2003).

17. Klinker, H., Haas, C., Harrer, N., Becker, P. B. & Mueller-Planitz, F. Rapid purification of recombinant histones. *PloS One* **9**, e104029 (2014).

18. Thumuluri, V., Almagro Armenteros, J. J., Johansen, A. R., Nielsen, H. & Winther, O. DeepLoc 2.0: multi-label subcellular localization prediction using protein language models. *Nucleic Acids Res.* **50**, W228–W234 (2022).

19. Guo, H.-B. & Guo, H. Mechanism of histone methylation catalyzed by protein lysine methyltransferase SET7/9 and origin of product specificity. *Proc. Natl. Acad. Sci. U.S.A.* **104**, 8797–8802 (2007).

20. Chu, Y., Yao, J. & Guo, H. QM/MM MD and free energy simulations of G9a-like protein (GLP) and its mutants: understanding the factors that determine the product specificity. *PLoS One* **7**, e37674 (2012).

21. Word, J. M. *et al.* Visualizing and quantifying molecular goodness-of-fit: small-probe contact dots with explicit hydrogen atoms. *J Mol. Biol.* **285**, 1711–1733 (1999).

22. Goldstein, H., Poole, C. & Safko, J. *Classical mechanics* (American Association of Physics Teachers, 2002).

23. Singh, P. R. *et al.* The *Mycobacterium tuberculosis* methyltransferase Rv2067c manipulates host epigenetic programming to promote its own survival. zenodo, DOI:10.5281/zenodo.8352903 (2023).

24. Emsley, P., Lohkamp, B., Scott, W. G. & Cowtan, K. Features and development of Coot. *Acta Crystallogr. D Biol. Crystal.* **66**, 486–501 (2010).

25. Boratyn, G. M. *et al.* BLAST: a more efficient report with usability improvements. *Nucleic Acids Res.* **41**, W29–W33 (2013).

26. Huang, Y., Niu, B., Gao, Y., Fu, L. & Li, W. CD-HIT Suite: a web server for clustering and comparing biological sequences. *Bioinformatics* **26**, 680–682 (2010).

27. Sievers, F. *et al.* Fast, scalable generation of high-quality protein multiple sequence alignments using Clustal Omega. *Mol. Syst. Biol.* **7**, 539 (2011).

28. Ashkenazy, H. *et al.* ConSurf 2016: an improved methodology to estimate and visualize evolutionary conservation in macromolecules. *Nucleic Acids Res.* **44**, W344–W350 (2016).

29. Waterhouse, A. M., Procter, J. B., Martin, D. M., Clamp, M. & Barton, G. J. Jalview Version 2—a multiple sequence alignment editor and analysis workbench. *Bioinformatics* **25**, 1189–1191 (2009).

30. Jumper, J. *et al.* Highly accurate protein structure prediction with AlphaFold. *Nature* **596**, 583–589 (2021).

31. Jorgensen, W. L., Chandrasekhar, J., Madura, J. D., Impey, R. W. & Klein, M. L. Comparison of simple potential functions for simulating liquid water. *J Chem. Phys.* **79**, 926–935 (1983).

32. Huang, J. *et al.* CHARMM36m: an improved force field for folded and intrinsically disordered proteins. *Nat. Methods* **14**, 71–73 (2017).

33. Bussi, G., Donadio, D. & Parrinello, M. Canonical sampling through velocity rescaling. *J Chem. Phys.* **126** (2007).

34. Parrinello, M. & Rahman, A. Polymorphic transitions in single crystals: A new molecular dynamics method. *J Appl. Phys.* **52**, 7182–7190 (1981).

35. Abraham, M. J. *et al.* GROMACS: High performance molecular simulations through multi-level parallelism from laptops to supercomputers. *SoftwareX* **1**, 19–25 (2015).

36. Roe, D. R. & Cheatham III, T. E. PTRAJ and CPPTRAJ: software for processing and analysis of molecular dynamics trajectory data. *J Chem. Theory Comput.* **9**, 3084–3095 (2013).

37. Wagner, J. R. *et al.* POVME 3.0: software for mapping binding pocket flexibility. *J Chem. Theory Comput.* **13**, 4584–4592 (2017).

38. Goddard, T. D. *et al.* UCSF ChimeraX: Meeting modern challenges in visualization and analysis. *Protein Sci.* **27**, 14–25 (2018).

39. Maier, J. A. *et al.* ff14SB: improving the accuracy of protein side chain and backbone parameters from ff99SB. *J Chem. Theory Comput.* **11**, 3696–3713 (2015).

40. Salomon-Ferrer, R., Gotz, A. W., Poole, D., Le Grand, S. & Walker, R. C. Routine microsecond molecular dynamics simulations with AMBER on GPUs. 2. Explicit solvent particle mesh Ewald. *J Chem. Theory Comput.* **9**, 3878–3888 (2013).

41. Saez, D. A. & Vöhringer-Martinez, E. A consistent S-Adenosylmethionine force field improved by dynamic Hirshfeld-I atomic charges for biomolecular simulation. *J Comput. Aided Mol. Des.* **29**, 951–961 (2015).

42. Belisle, J. T., Mahaffey, S. B. & Hill, P. J. Isolation of mycobacterium species genomic DNA. *Methods Mol. Biol.* 1–12 (2009).

43. Chen, S., Zhou, Y., Chen, Y. & Gu, J. fastp: an ultra-fast all-in-one FASTQ preprocessor. *Bioinformatics* **34**, i884–i890 (2018).

44. Ewels, P., Magnusson, M., Lundin, S. & Käller, M. MultiQC: summarize analysis results for multiple tools and samples in a single report. *Bioinformatics* **32**, 3047–3048 (2016).

45. Dobin, A. *et al.* STAR: ultrafast universal RNA-seq aligner. *Bioinformatics* **29**, 15–21 (2013).

46. Liao, Y., Smyth, G. K. & Shi, W. featureCounts: an efficient general purpose program for assigning sequence reads to genomic features. *Bioinformatics* **30**, 923–930 (2014).

47. Robinson, M. D., McCarthy, D. J. & Smyth, G. K. edgeR: a Bioconductor package for differential expression analysis of digital gene expression data. *Bioinformatics* **26**, 139–140 (2010).
