# Supplementary material for: The Mycobacterium tuberculosis methyltransferase Rv2067c manipulates host epigenetic programming to promote its own survival
Source: Nat Commun. 2023 Dec 21;14:8497. doi: 10.1038/s41467-023-43940-6 (PMC10739865; doi:10.1038/s41467-023-43940-6)
Supplement: Supplementary file 3 — Description of Additional Supplementary Files [file 41467_2023_43940_MOESM3_ESM.pdf]

Description of Additional Supplementary Files:

Supplementary Data 1: List of antibodies used in the present study.

Supplementary Data 2: List of primers/oligo nucleotides used in the present study.

Supplementary Data 3: Coordinates of the Rv2067c crystal structure (PDB format).

Supplementary Data 4: Coordinates of Rv2067c-H3 peptide model (PDB format).

Supplementary Movie 1: Overall structure and active site dynamics of Rv2067c. Dynamics were shown for monomer A (chain A).

Supplementary Software File 1: Analytical GFC plotting

Supplementary Software File 2: Temperature factors data plotting

Supplementary Software File 3: RMSF data plotting
