# Supplementary material for: The Mycobacterium tuberculosis methyltransferase Rv2067c manipulates host epigenetic programming to promote its own survival
Source: Nat Commun. 2023 Dec 21;14:8497. doi: 10.1038/s41467-023-43940-6 (PMC10739865; doi:10.1038/s41467-023-43940-6)
Supplement: Supplementary file 9 — Reporting Summary [file 41467_2023_43940_MOESM9_ESM.pdf]

nature portfolio

Corresponding author(s): V. Nagaraja

Last updated by author(s): 17/11/23

Reporting Summary

Nature Portfolio wishes to improve the reproducibility of the work that we publish. This form provides structure for consistency and transparency in reporting. For further information on Nature Portfolio policies, see our Editorial Policies and the Editorial Policy Checklist.

Statistics

For all statistical analyses, confirm that the following items are present in the figure legend, table legend, main text, or Methods section.

n/a	Confirmed	
☐	☒	The exact sample size (*n*) for each experimental group/condition, given as a discrete number and unit of measurement
☐	☒	A statement on whether measurements were taken from distinct samples or whether the same sample was measured repeatedly
☐	☒	The statistical test(s) used AND whether they are one- or two-sided<br>*Only common tests should be described solely by name; describe more complex techniques in the Methods section.*
☒	☐	A description of all covariates tested
☒	☐	A description of any assumptions or corrections, such as tests of normality and adjustment for multiple comparisons
☐	☒	A full description of the statistical parameters including central tendency (e.g. means) or other basic estimates (e.g. regression coefficient) AND variation (e.g. standard deviation) or associated estimates of uncertainty (e.g. confidence intervals)
☐	☒	For null hypothesis testing, the test statistic (e.g. *F*, *t*, *r*) with confidence intervals, effect sizes, degrees of freedom and *P* value noted<br>*Give P values as exact values whenever suitable.*
☒	☐	For Bayesian analysis, information on the choice of priors and Markov chain Monte Carlo settings
☒	☐	For hierarchical and complex designs, identification of the appropriate level for tests and full reporting of outcomes
☒	☐	Estimates of effect sizes (e.g. Cohen's *d*, Pearson's *r*), indicating how they were calculated

Our web collection on statistics for biologists contains articles on many of the points above.

Software and code

Policy information about availability of computer code

Data collection: GROMACS, custom python script for rotation scan are available at https://github.com/Venkat-Dadi/Rotation_Scan.

Data analysis:

Software	Version
XDSAPP3.0	v1-8-2022-08-23
CRANK2	2.0.253
PHASER	2.8.3
Coot	0.9.6
REFMAC5	5.8.0267
GROMACS	2021.2
AMBER20	-
CPPTRAJ	V5.1.0
POVME3	3.0.34
ConSurf	web server
CD-HIT	web server
BLAST	web server
ChimeraX	1.3rc202111230539
GraphPad Prism	v/7 and 9.1.1
Microsoft excel	
FastQC	

DAVID	2021
edgeR	
STAR2	
MultiQC	

For manuscripts utilizing custom algorithms or software that are central to the research but not yet described in published literature, software must be made available to editors and reviewers. We strongly encourage code deposition in a community repository (e.g. GitHub). See the Nature Portfolio guidelines for submitting code & software for further information.

Data

Policy information about availability of data

All manuscripts must include a data availability statement. This statement should provide the following information, where applicable:

- Accession codes, unique identifiers, or web links for publicly available datasets
- A description of any restrictions on data availability
- For clinical datasets or third party data, please ensure that the statement adheres to our policy

Source Data for sequencing reads is available under accession code PRJNA907927 [https://www.ncbi.nlm.nih.gov/sra/PRJNA907927] (Raw reads for RNA sequencing of THP1 infected with Mycobacterium expressing Rv2067c). All preliminary results are available in Analysed Transcriptomic Data (Source Data 5)
Accession code: Coordinates for Rv2067c-SAH are deposited in the Protein Data Bank under the accession code 8HKR.
Other PDB codes used in this study are as follows
8HKR [https://doi.org/10.2210/pdb8HKR/pdb] (Rv2067c-SAH)
1NW3 [https://doi.org/10.2210/pdb1NW3/pdb] (DOT1L)
AF-Q8TEK3-F1 [https://alphafold.ebi.ac.uk/entry/Q8TEK3] (human DOT1L full-length AlphaFold2 model)
6NJ9 [https://doi.org/10.2210/pdb6NJ9/pdb] (Ubiquitinated nucleosome and DOT1L complex)
5E1B [https://doi.org/10.2210/pdb5E1B/pdb] (NRMT1, Supplementary Figure 9)
2NXN [https://doi.org/10.2210/pdb2NXN/pdb] (PrmA, Supplementary Figure 9)
3EGV [https://doi.org/10.2210/pdb3EGV/pdb] (PrmA, Supplementary Figure 9)
3P71 [https://doi.org/10.2210/pdb3P71/pdb] (LCMT, Supplementary Figure 9)
5DX0 [https://doi.org/10.2210/pdb5DX0/pdb] (PRMT, Supplementary Figure 9)
6H1E [https://doi.org/10.2210/pdb6H1E/pdb] (KMT9, Supplementary Figure 9)
2F69 [https://doi.org/10.2210/pdb2F69/pdb] (SET7/9, Supplementary Figure 9)
1KX5 [https://doi.org/10.2210/pdb1KX5/pdb] (Histone H3, chain A, Supplementary Figure 12)
5DPD [https://doi.org/10.2210/pdb5DPD/pdb] (PKMT1, Supplementary Figure 17)
5DPL [https://doi.org/10.2210/pdb5DPL/pdb] (PKMT2, Supplementary Figure 17)
3BUS [https://doi.org/10.2210/pdb3BUS/pdb] (RebM, Supplementary Figure 18)

All the data generated and/or analyzed during the current study is available in the Figure 1-10 and its supplementary figures, supplementary movie 1 , supplementary information and supplementary data 1-4. Source data is provided.

Research involving human participants, their data, or biological material

Policy information about studies with human participants or human data. See also policy information about sex, gender (identity/presentation), and sexual orientation and race, ethnicity and racism.

Reporting on sex and gender	Not applicable for this study.
Reporting on race, ethnicity, or other socially relevant groupings	Not applicable for this study.
Population characteristics	Not applicable for this study.
Recruitment	Not applicable for this study.
Ethics oversight	Not applicable for this study.

Note that full information on the approval of the study protocol must also be provided in the manuscript.

Field-specific reporting

Please select the one below that is the best fit for your research. If you are not sure, read the appropriate sections before making your selection.

☒ Life sciences ☐ Behavioural & social sciences ☐ Ecological, evolutionary & environmental sciences

For a reference copy of the document with all sections, see nature.com/documents/nr-reporting-summary-flat.pdf

Life sciences study design

All studies must disclose on these points even when the disclosure is negative.

Sample size	For mice experiments, 6 BALB/cJ mice (6-8 week old) were included in each group. Animal number was sufficient to identify differences in bacterial burden as published previously and also conformed to the ethical guidelines.<br>1 New Zealand rabbit was used to generate Rv2067c antibody.
Data exclusions	No data exclusion was used
Replication	Reproducibility was confirmed by 2 or more independent repeats of the experiment.<br>For ChIP and qRT, data shown is mean ± standard deviation of two independent sets of infection. For qRT-PCR, two technical replicates per sample were kept.<br>Two independent THP1 infections were performed for RNA isolation and RNA sequencing<br>For Scintillation counts , data plotted is mean ± standard deviation for 3 independent experiments.<br>Immunostaining was performed 3 times and multiple fields were captured.<br>The exact replicate numbers are mentioned in figure legends.
Randomization	Mice used were BALB/cJ mice (6-8 week old) which were inbred and genetically identical. Mice were distributed randomly into different groups.
Blinding	Not applicable for the experiments performed in this study.

Reporting for specific materials, systems and methods

We require information from authors about some types of materials, experimental systems and methods used in many studies. Here, indicate whether each material, system or method listed is relevant to your study. If you are not sure if a list item applies to your research, read the appropriate section before selecting a response.

Materials & experimental systems

n/a	Involved in the study	
☐	☒	Antibodies
☐	☒	Eukaryotic cell lines
☒	☐	Palaeontology and archaeology
☐	☒	Animals and other organisms
☒	☐	Clinical data
☒	☐	Dual use research of concern
☒	☐	Plants

Methods

n/a	Involved in the study	
☒	☐	ChIP-seq
☐	☒	Flow cytometry
☒	☐	MRI-based neuroimaging

Antibodies

Antibodies used	FLAG (#F3165 Sigma, 1:10,000); 6x-His (#MA1-21315 Invitrogen, 1:10,000); H3K79me3 (#C15410068 Diagenode, 1:1,000); H3K4me3 (#49-1005 Thermo Fisher, 1:1,000); H3K9me3 (#49-1008 Thermo Fisher, 1:1,000); H3K27me3(#9733 Cell Signaling Technology, 1:1,000); H3K36me3 (#PA5-96118 Thermo Fisher, 1:1,000); DOT1L (#MA5-35166 Invitrogen, 1:1,000); β-Actin(#A3854 Sigma, 1:1,000); Histone H3(#ab1791 Abcam, 1:10,000); Tubulin(#CAB870Hu22 cloud clone, 1:1,000; Rv2067c (Lab generated, 1:10,000); Rho (Lab generated, 1:10,000); GroEL1 (kind gift from Dr Shekhar Mande, NCCS, Pune, India, 1:10,000); Alexa Fluor 488 (#A32723 Invitrogen, 1:1,000); Alexa Fluor 568 (#A11004 and A11011 Invitrogen 1:1,000); BCL-xL(#sc-8392 Santa Cruz Biotechnology, 1:1,000); BCL2 (#sc-7382 Santa Cruz Biotechnology, 1:1,000); BAX (#sc-7873 Santa Cruz Biotechnology, 1:1,000); BID (#sc-11423 Santa Cruz Biotechnology, 1:1,000); TNF-a (#AMC3012 Invitrogen, 1:1,000); Caspase 8 (#D35G2 Cell Signaling Technology, 1:1,000); RIPK1 (#MAE640Hu21Cloud-Clone Corp., 1:1,000); RIPK3 (#PAE639Hu01 Cloud-Clone Corp., 1:1,000); FADD(#PAK078Hu01 Cloud-Clone Corp., 1:1,000)FLAG (#F3165 Sigma, 1:10,000); 6x-His (#MA1-21315 Invitrogen, 1:10,000); H3K79me3 (#C15410068 Diagenode, 1:1,000); H3K4me3(#49-1005 Thermo Fisher, 1:1,000); H3K9me3 (#49-1008 Thermo Fisher, 1:1,000); H3K27me3(#9733 Cell Signaling Technology, 1:1,000); H3K36me3 (#PA5-96118 Thermo Fisher, 1:1,000); DOT1L (#MA5-35166 Invitrogen, 1:1,000); β-Actin(#A3854 Sigma, 1:1,000); Histone H3(#ab1791 Abcam, 1:10,000); Tubulin(#CAB870Hu22 cloud clone, 1:1,000; Rv2067c (Lab generated, 1:10,000); Rho (Lab generated, 1:10,000); GroEL1 (kind gift from Dr Shekhar Mande, NCCS, Pune, India, 1:10,000); Alexa Fluor 488 (#A32723 Invitrogen, 1:1,000); Alexa Fluor 568 (#A11004 and A11011 Invitrogen 1:1,000); BCL-xL(#sc-8392 Santa Cruz Biotechnology, 1:1,000); BCL2 (#sc-7382 Santa Cruz Biotechnology, 1:1,000); BAX (#sc-7873 Santa Cruz Biotechnology, 1:1,000); BID (#sc-11423 Santa Cruz Biotechnology, 1:1,000); TNF-a (#AMC3012 Invitrogen, 1:1,000); Caspase 8 (#9496 Cell Signaling Technology, 1:1,000); RIPK1 (#MAE640Hu21Cloud-Clone Corp., 1:1,000); RIPK3 (#PAE639Hu01 Cloud-Clone Corp., 1:1,000); FADD(#PAK078Hu01 Cloud-Clone Corp., 1:1,000).
Validation	Antibody against Rv2067 was raised in rabbit and its specificity is shown in Supplementary Figure 3e.<br>Validation information for the other commercial antibodies is available on the manufacturer's website.

Eukaryotic cell lines

Policy information about cell lines and Sex and Gender in Research

Cell line source(s)	THP-1 and HEK293T cell lines was purchased from ATCC
Authentication	The cell line was not authenticated by us
Mycoplasma contamination	Mycoplasma contamination was ruled out on monthly basis
Commonly misidentified lines (See ICLAC register)	No misidentified cell line was used in this study

Animals and other research organisms

Policy information about studies involving animals; ARRIVE guidelines recommended for reporting animal research, and Sex and Gender in Research

Laboratory animals	6-8 week old, BALB/cJ mice were used for infection. Infected mice were kept in animal facility at BioSafety Levels -3 (BSL-3). (Approval number: CAF/Ethics/850/2021)<br>For generating Rv2067c antibody, female rabbit (New Zealand) was used. (Approval number: CAF/Ethics/620/2018).<br>The animals were maintained at animal care facility, Indian Institute of Science. The facility adheres to animal welfare standards and guidelines. 12 hr light/dark cycle, ambient temp (23-25 degree Celsius) and humidity of 50-60 % was maintained.
Wild animals	No wild animals were used in this study.
Reporting on sex	Both male and female mice were used for the study.
Field-collected samples	Field-collected samples were not used in this study.
Ethics oversight	All animal experiments were carried out in strict accordance with the guidelines prescribed by the Committee for the Purpose of Control and Supervision of Experiments on Animals(CPCSEA), Government of India. Experiments were carried out in a biosafety level 3 containment facility and approved by the Institutional Animal Ethical Committee (IAEC), Indian Institute of Science (IISc).

Note that full information on the approval of the study protocol must also be provided in the manuscript.

Plants

Seed stocks	Not applicable for this study
Novel plant genotypes	Not applicable for this study
Authentication	Not applicable for this study

Flow Cytometry

Plots

Confirm that:

- [x] The axis labels state the marker and fluorochrome used (e.g. CD4-FITC).
- [x] The axis scales are clearly visible. Include numbers along axes only for bottom left plot of group (a 'group' is an analysis of identical markers).
- [x] All plots are contour plots with outliers or pseudocolor plots.
- [x] A numerical value for number of cells or percentage (with statistics) is provided.

Methodology

Sample preparation	Single cell suspensions of infected macrophages were incubated with the indicated dyes in the indicated buffers followed by flow cytometry analysis. The detailed experimental procedure is described in the methods.
Instrument	FACSAria Fusion (BD Biosciences)

Software	FACSDiva
Cell population abundance	Not applicable
Gating strategy	Forward versus side scatter (FSC vs SSC) gating was used to identify cells of interest and exclude debris and dead cells

☒ Tick this box to confirm that a figure exemplifying the gating strategy is provided in the Supplementary Information.
