# Supplementary material for: The Mycobacterium tuberculosis methyltransferase Rv2067c manipulates host epigenetic programming to promote its own survival
Source: Nat Commun. 2023 Dec 21;14:8497. doi: 10.1038/s41467-023-43940-6 (PMC10739865; doi:10.1038/s41467-023-43940-6)
Supplement: Supplementary file 13 — Source Data [file 41467_2023_43940_MOESM13_ESM.zip › Source data File/Source data 2 - uncropped and unprocessed versions of blots with markers blots for main and supplementary figures.pdf]

Fig 1a

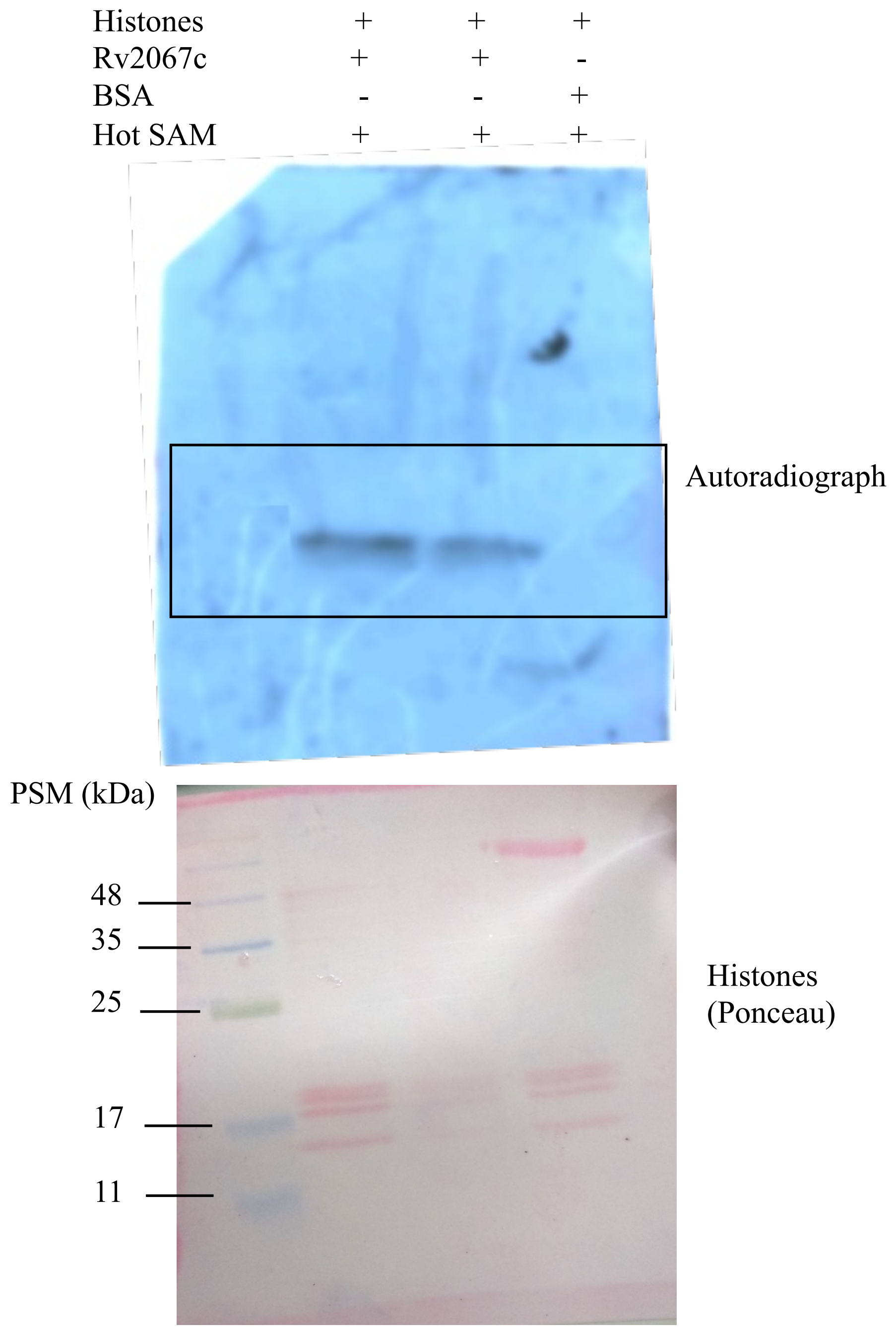

Fig 1c

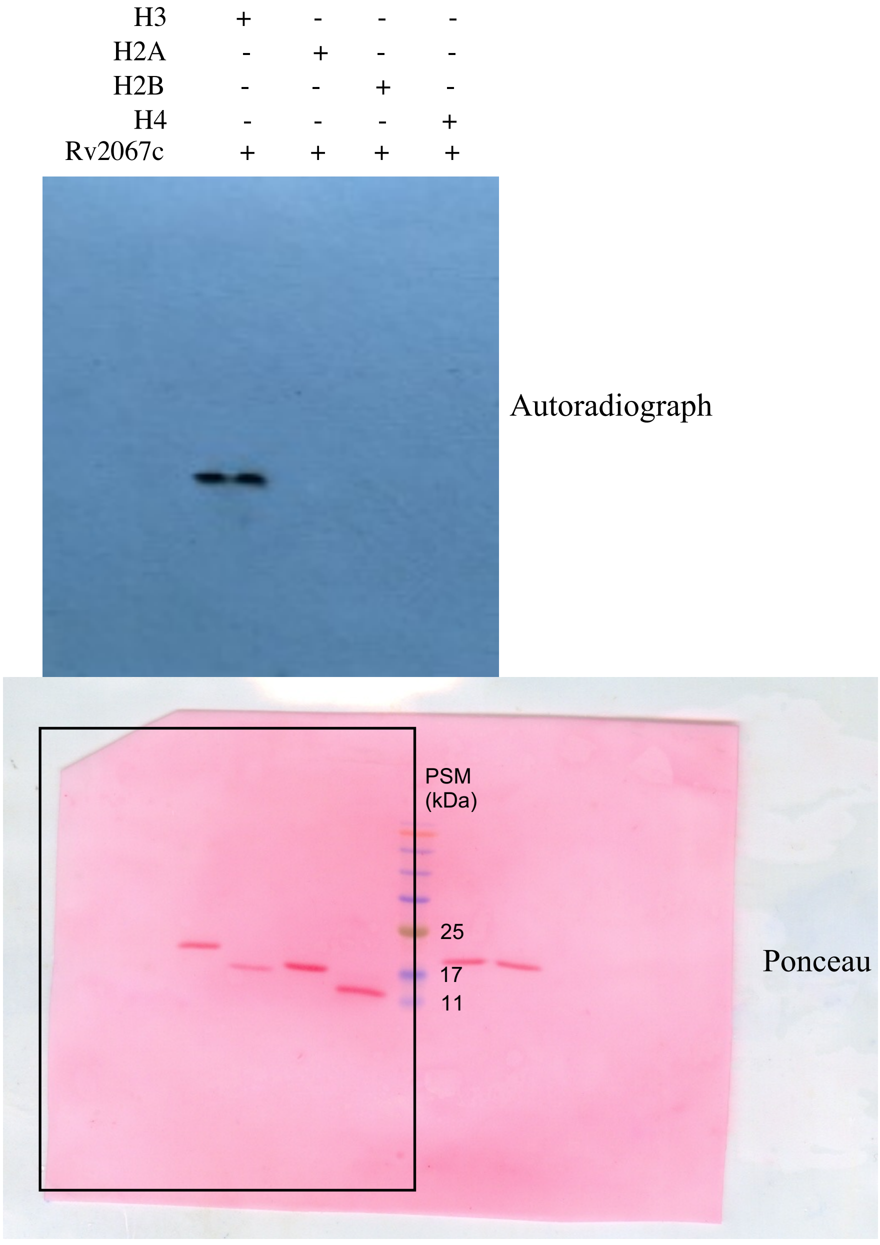

Fig 1e

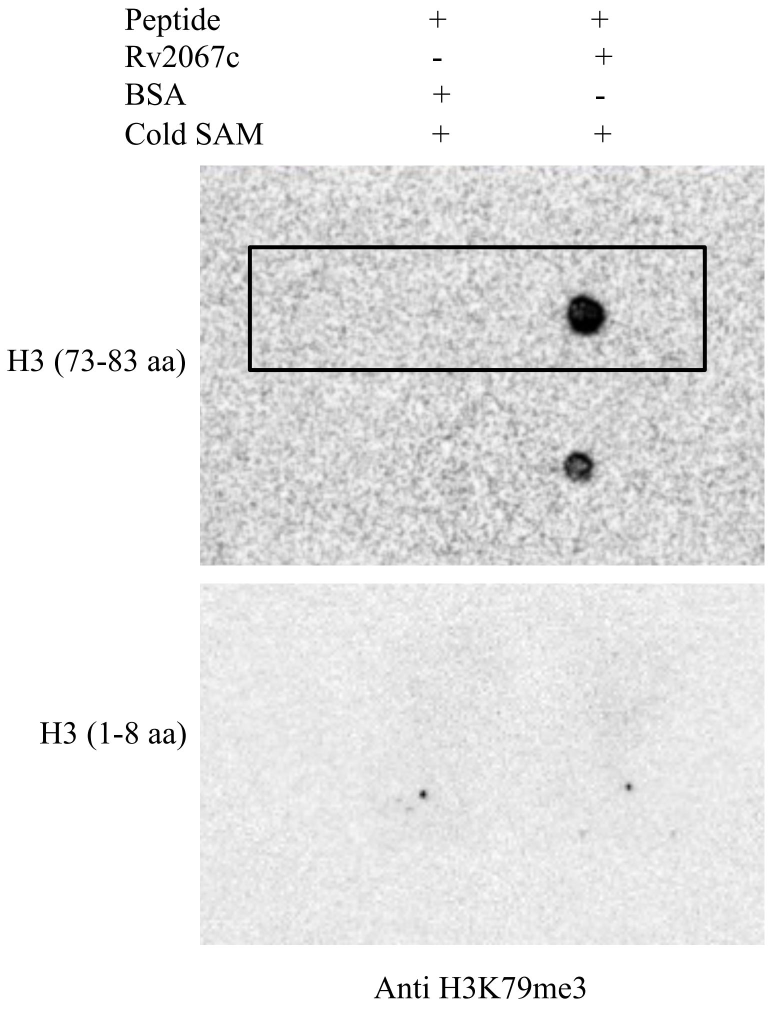

Figure 1. Rv2067c *in vitro* methylates histone H3 at lysine 79.

Fig. 2a

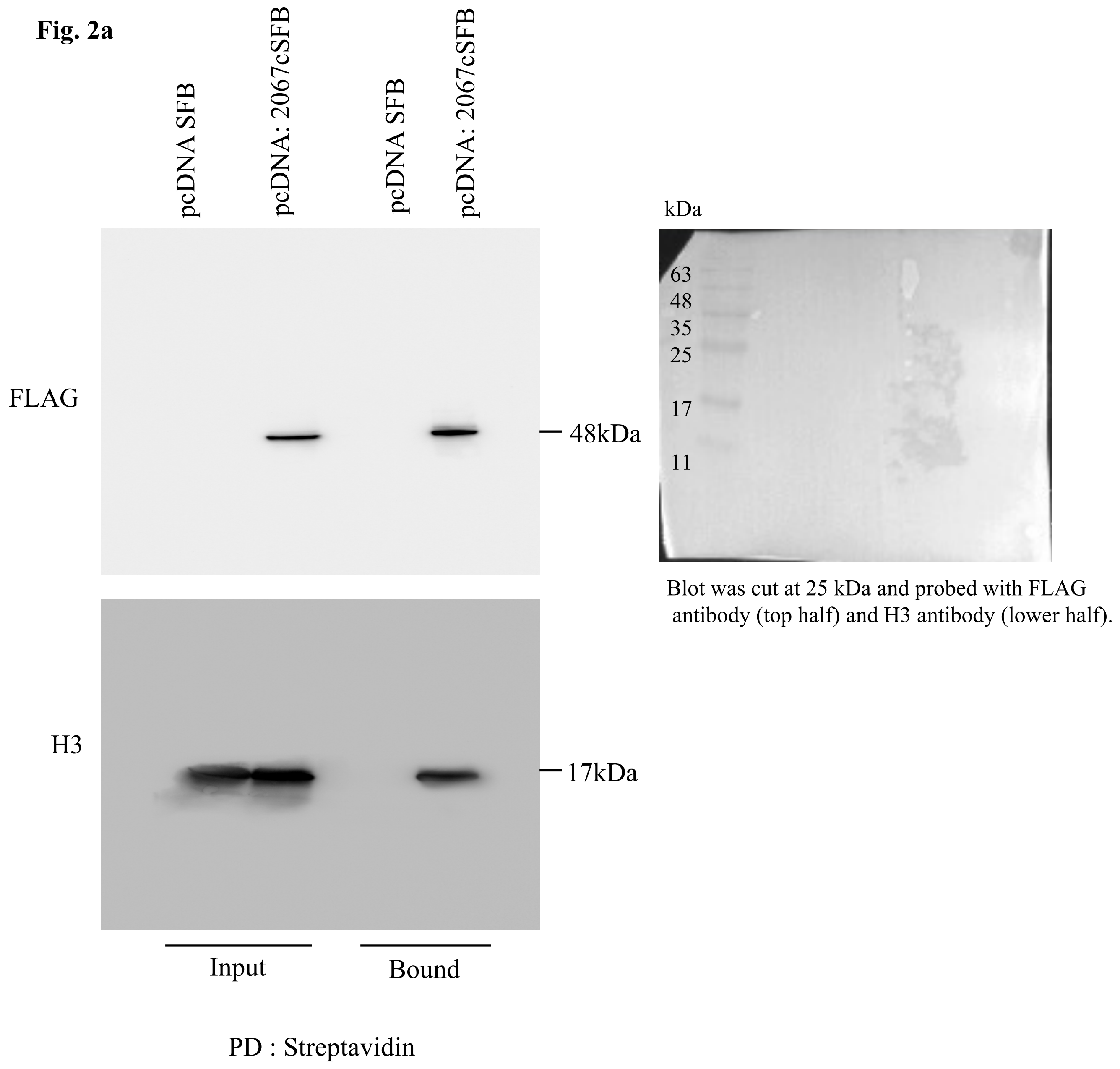

Blot was cut at 25 kDa and probed with FLAG antibody (top half) and H3 antibody (lower half).

Fig. 2b

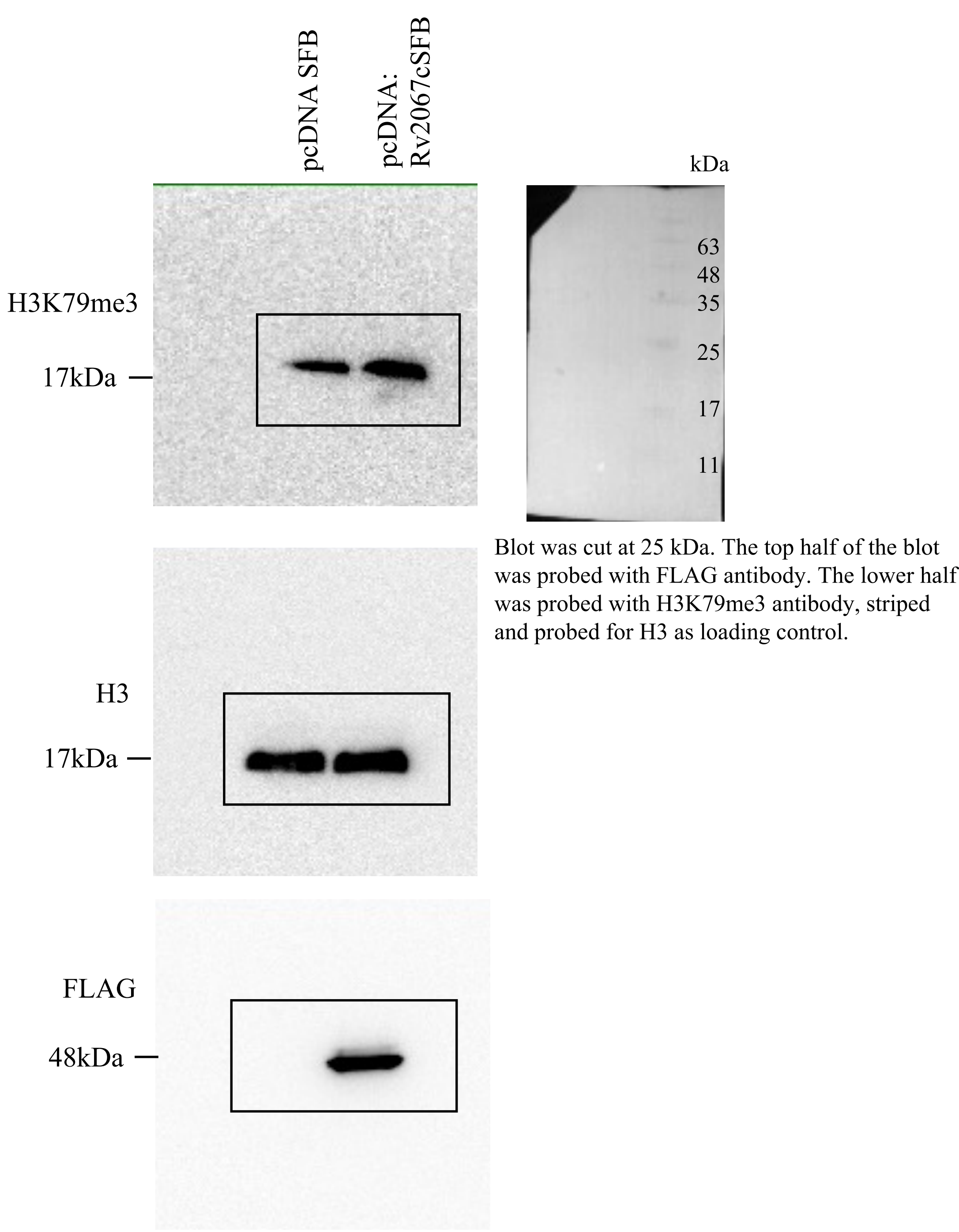

Blot was cut at 25 kDa. The top half of the blot was probed with FLAG antibody. The lower half was probed with H3K79me3 antibody, striped and probed for H3 as loading control.

Fig. 2c

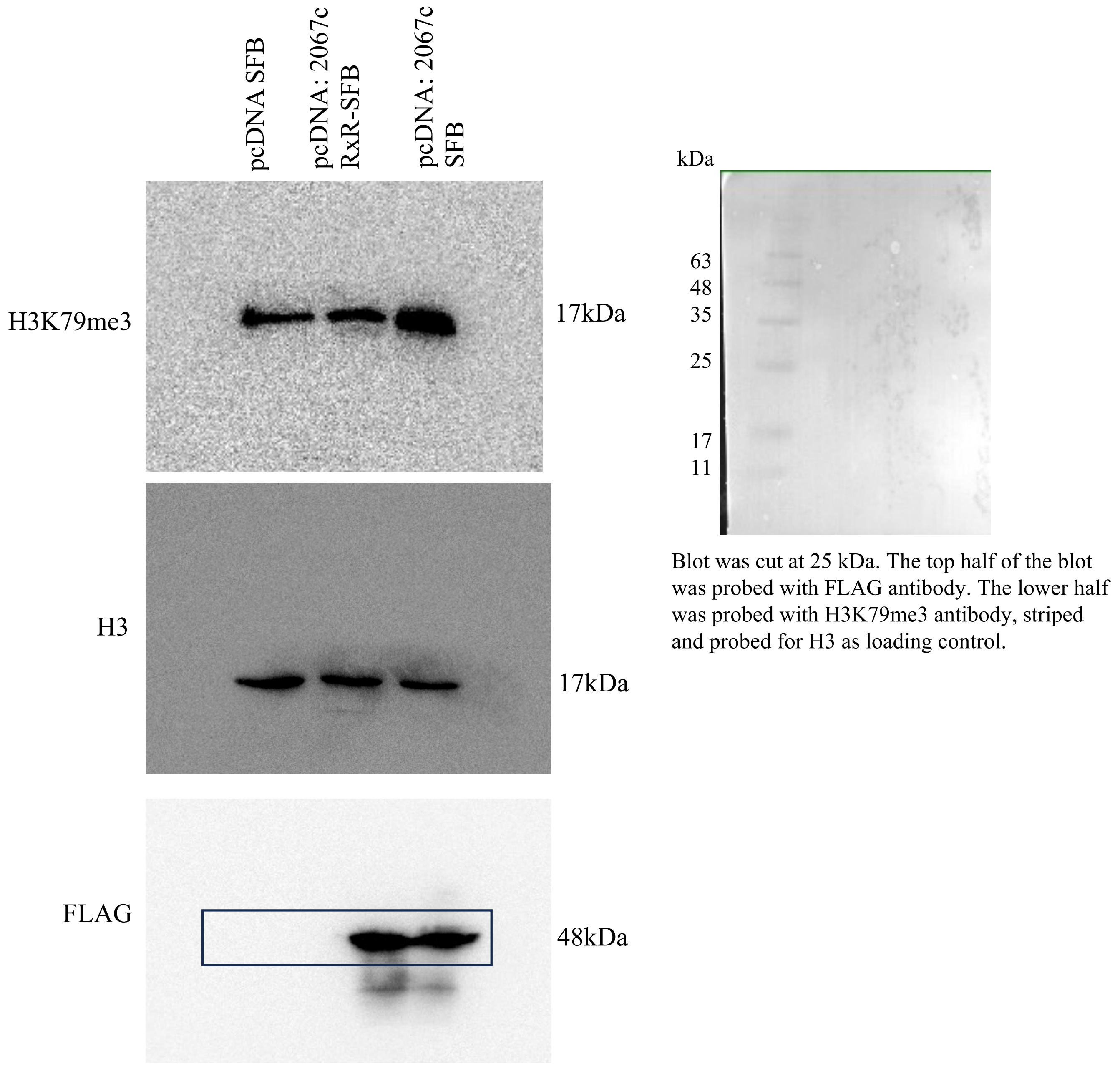

Blot was cut at 25 kDa. The top half of the blot was probed with FLAG antibody. The lower half was probed with H3K79me3 antibody, striped and probed for H3 as loading control.

Fig. 2e

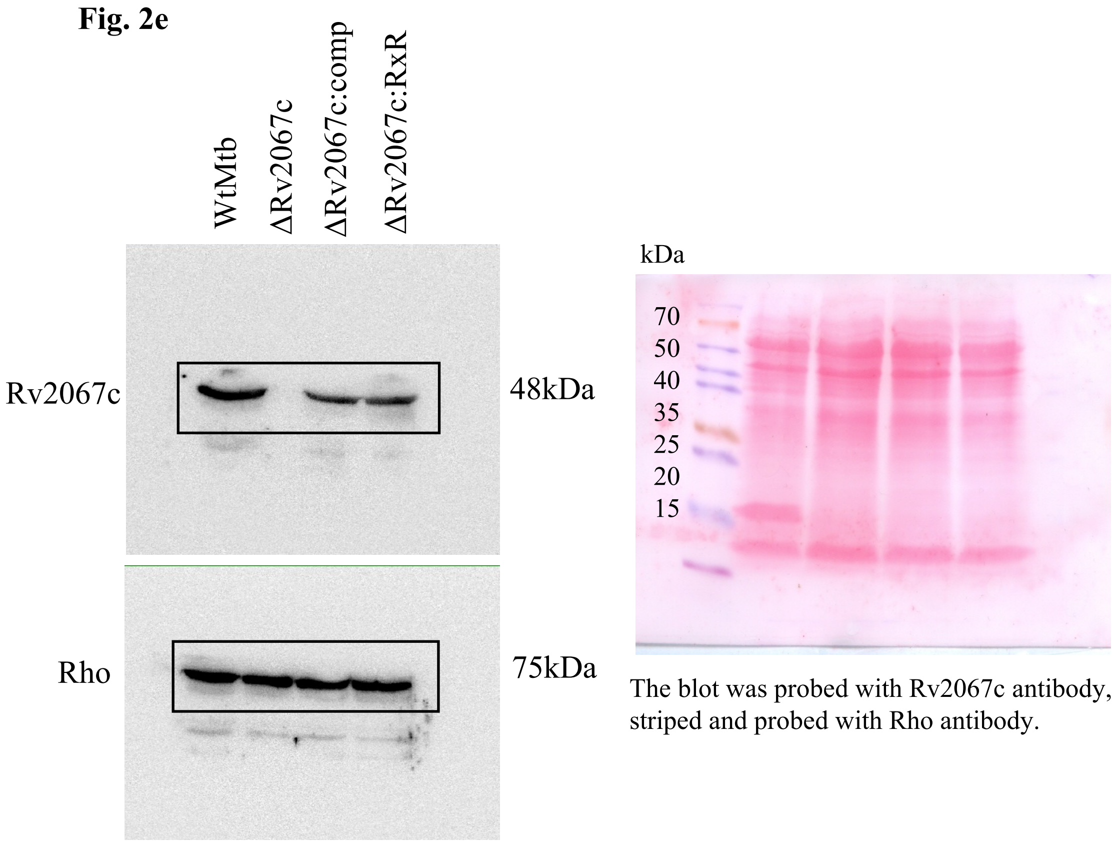

The blot was probed with Rv2067c antibody, striped and probed with Rho antibody.

Figure 2. Rv2067c methylates histone H3 at lysine 79 upon infection in THP1 macrophages

Fig. 2f

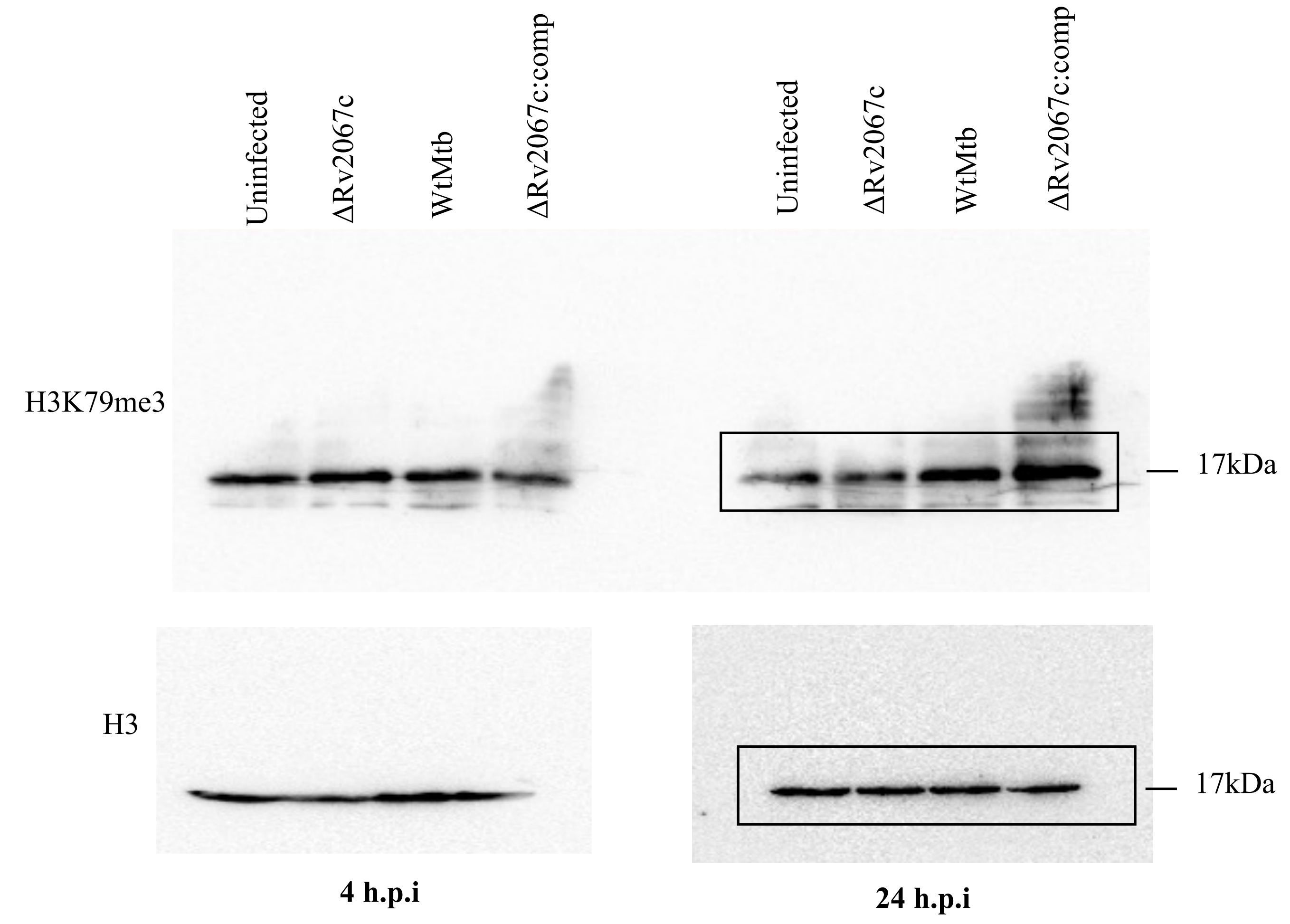

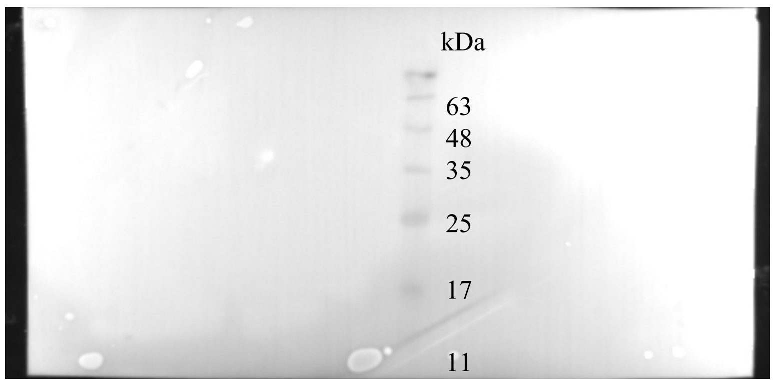

Blot was probed with H3K79me3, stripped and probed with H3 antibody

Fig. 2g

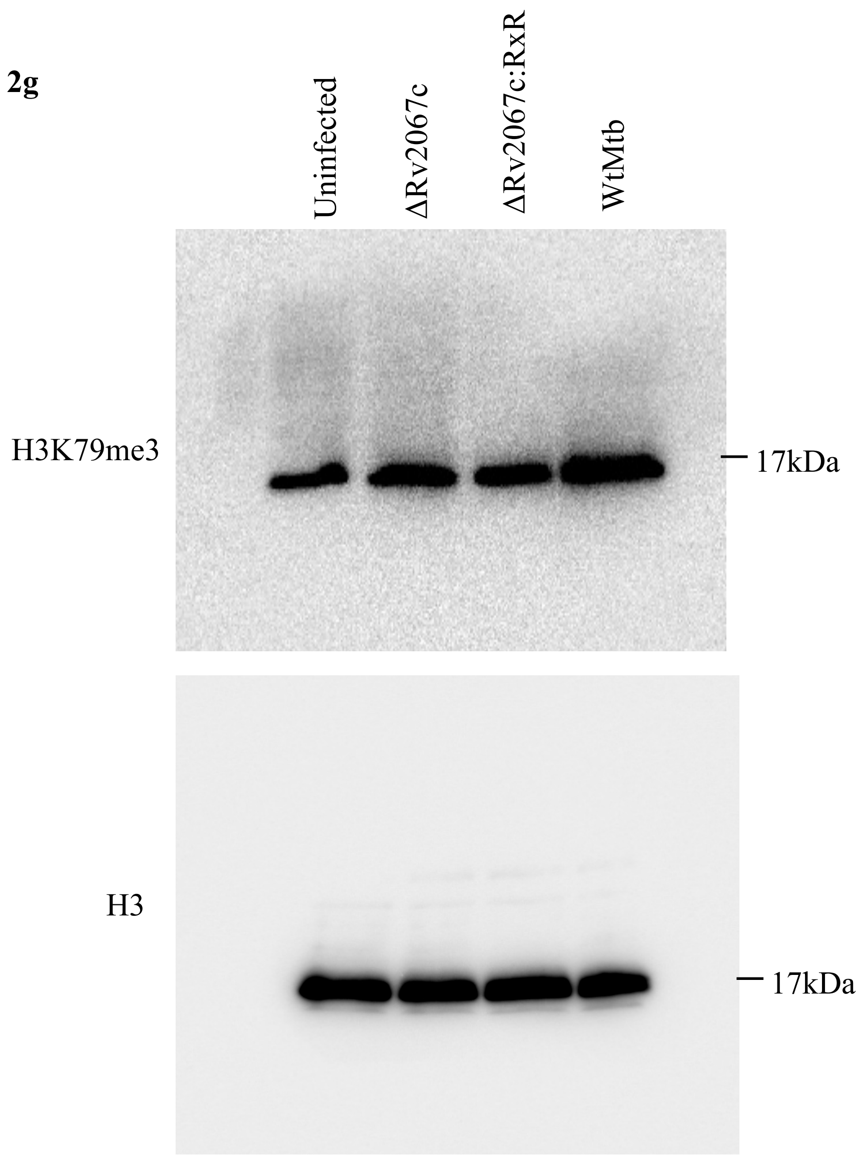

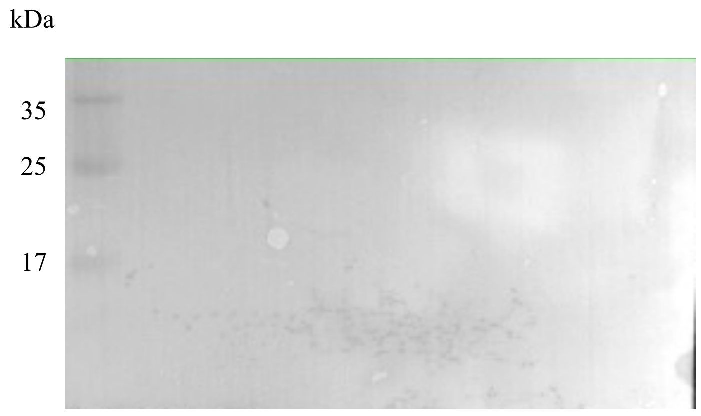

Blot was probed with H3K79me3, stripped and probed with H3 antibody

Figure 2. Rv2067c methylates histone H3 at lysine 79 upon infection in THP1 macrophages

Fig. 2g

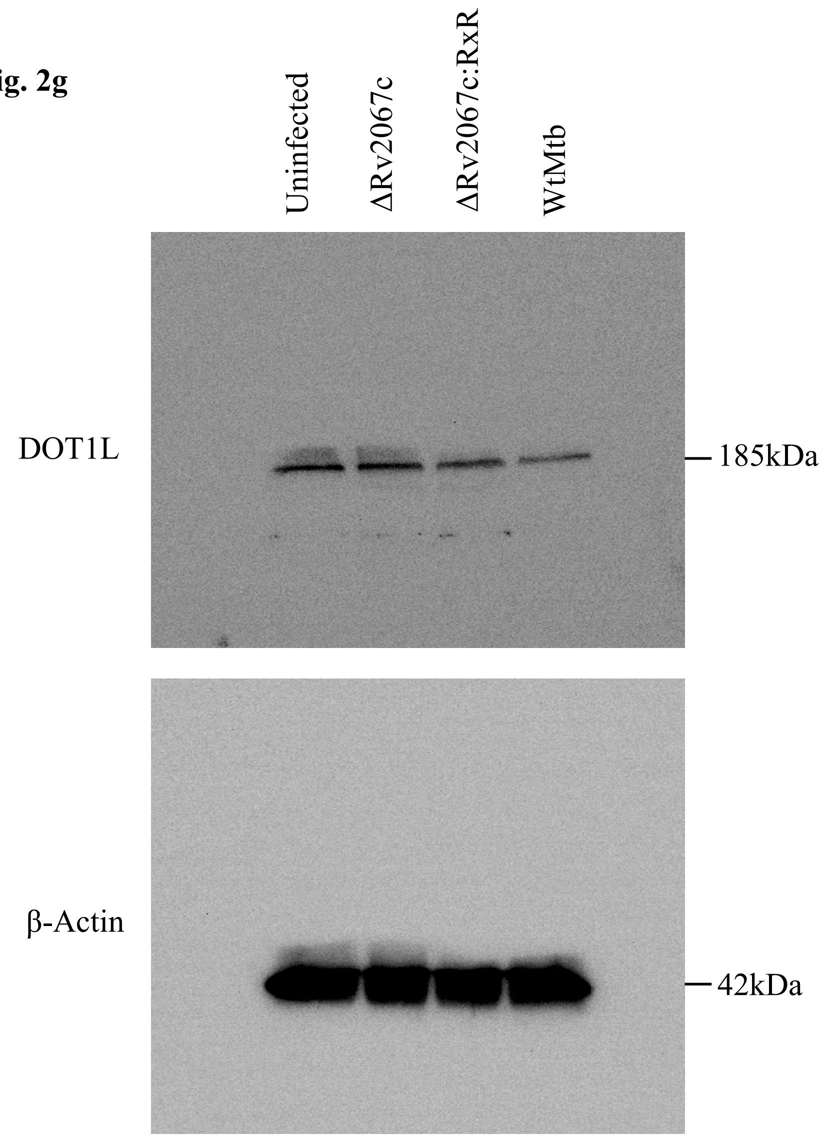

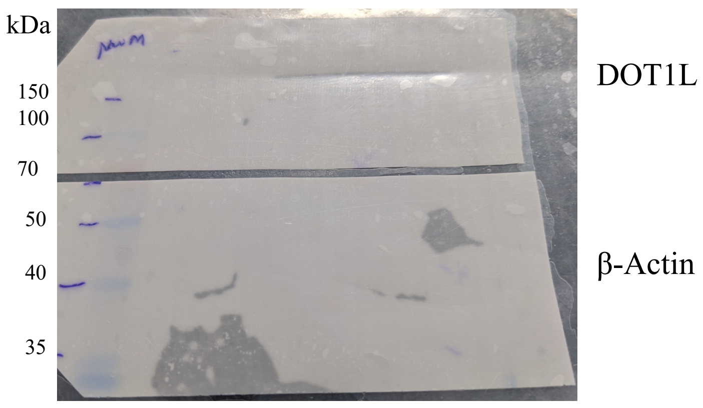

Blot was cut at 70KD and probed with DOT1L (upper half) and β-actin (lower half)

Fig 2h

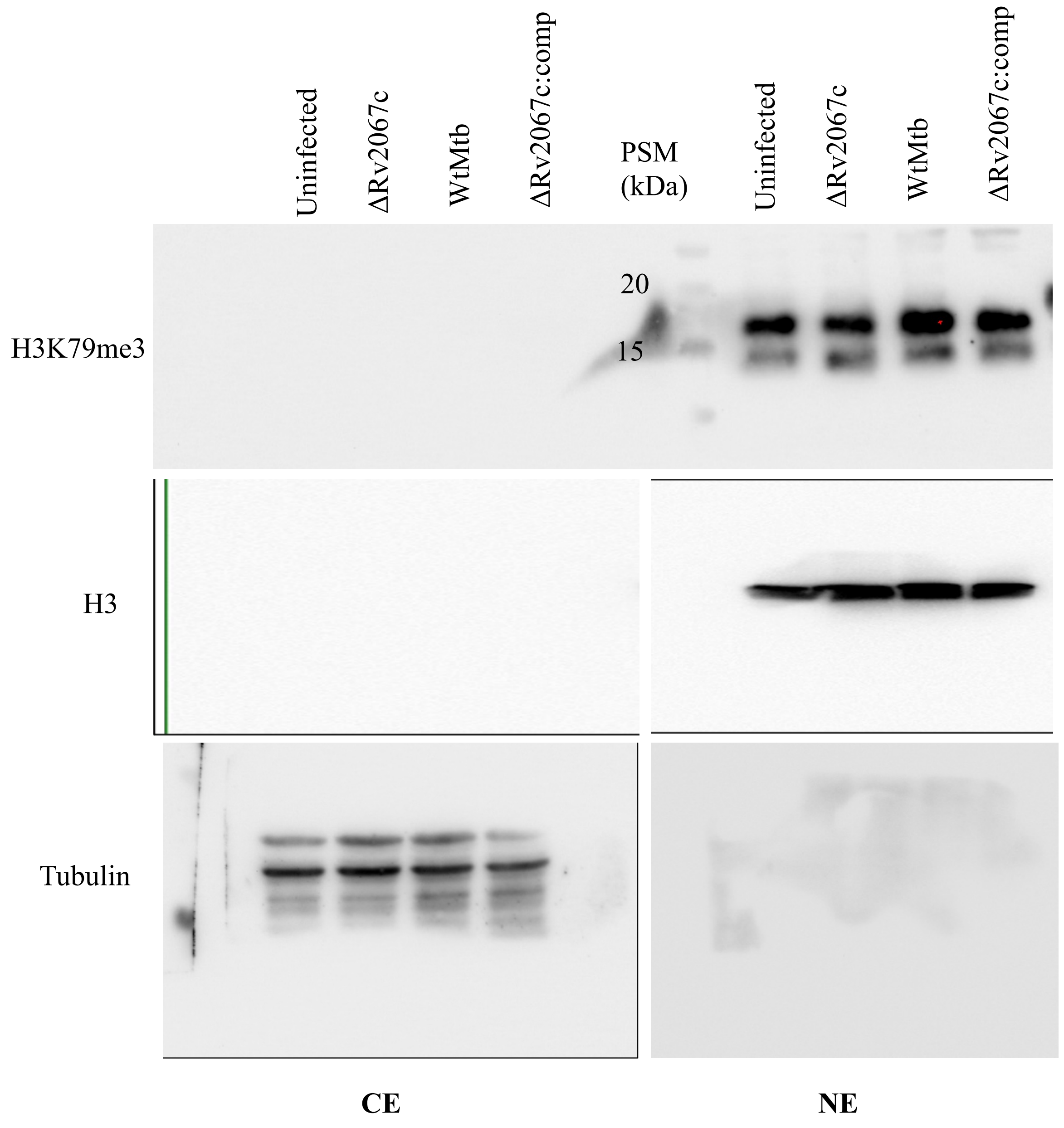

Figure 2. Rv2067c methylates histone H3 at lysine 79 upon infection in THP1 macrophages

Fig 2i

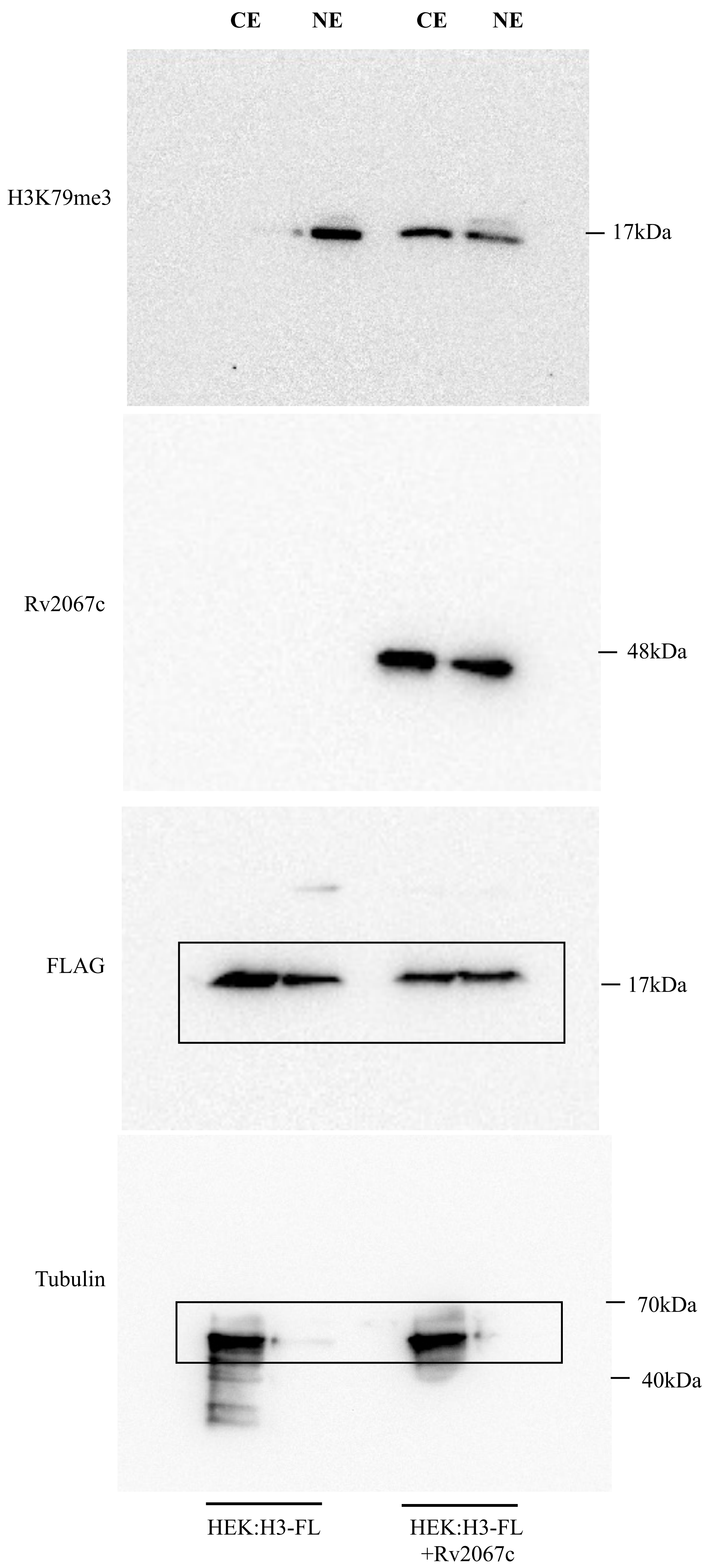

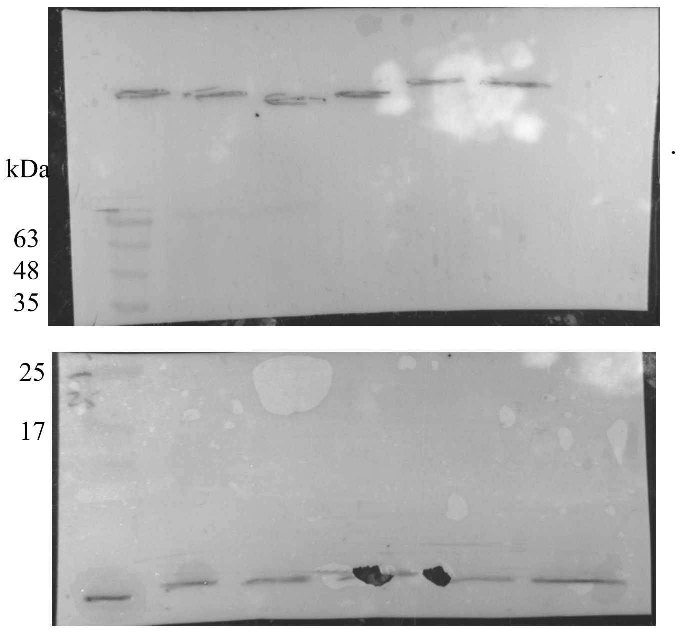

Blot was cut between 35 and 25kDa. Top half of the blot was probed with Rv2067c, stripped and probed tubulin antibody. Lower half of the blot was probed with H3K79me3,stripped and probed with FLAG antibody.

Figure 2. Rv2067c methylates histone H3 at lysine 79 upon infection in THP1 macrophages

Fig. 7a

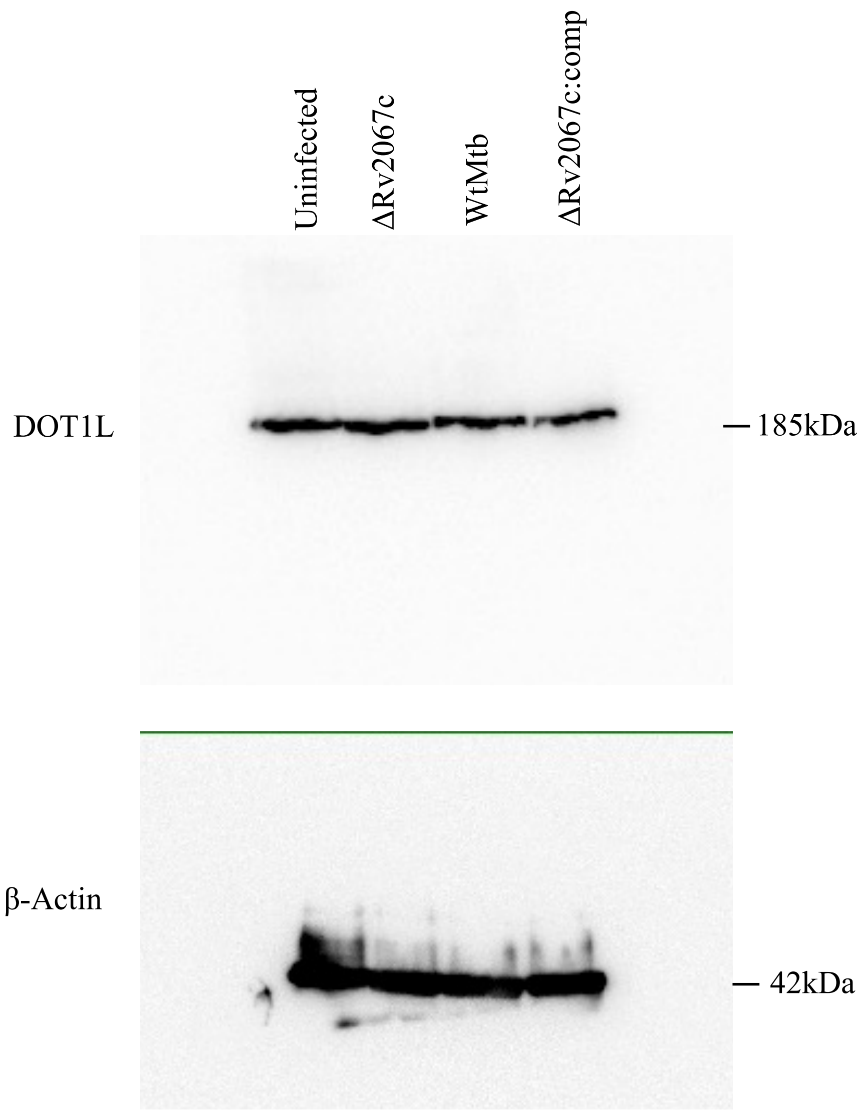

4 h.p.i

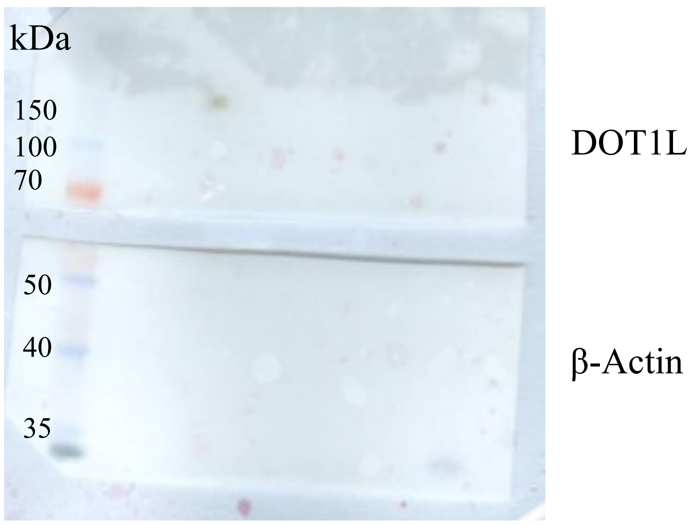

4 h.p.i

Blot was cut in-between 70 and 50 kDa and probed with DOT1L (upper half) and β-actin (lower half)

Fig. 7a

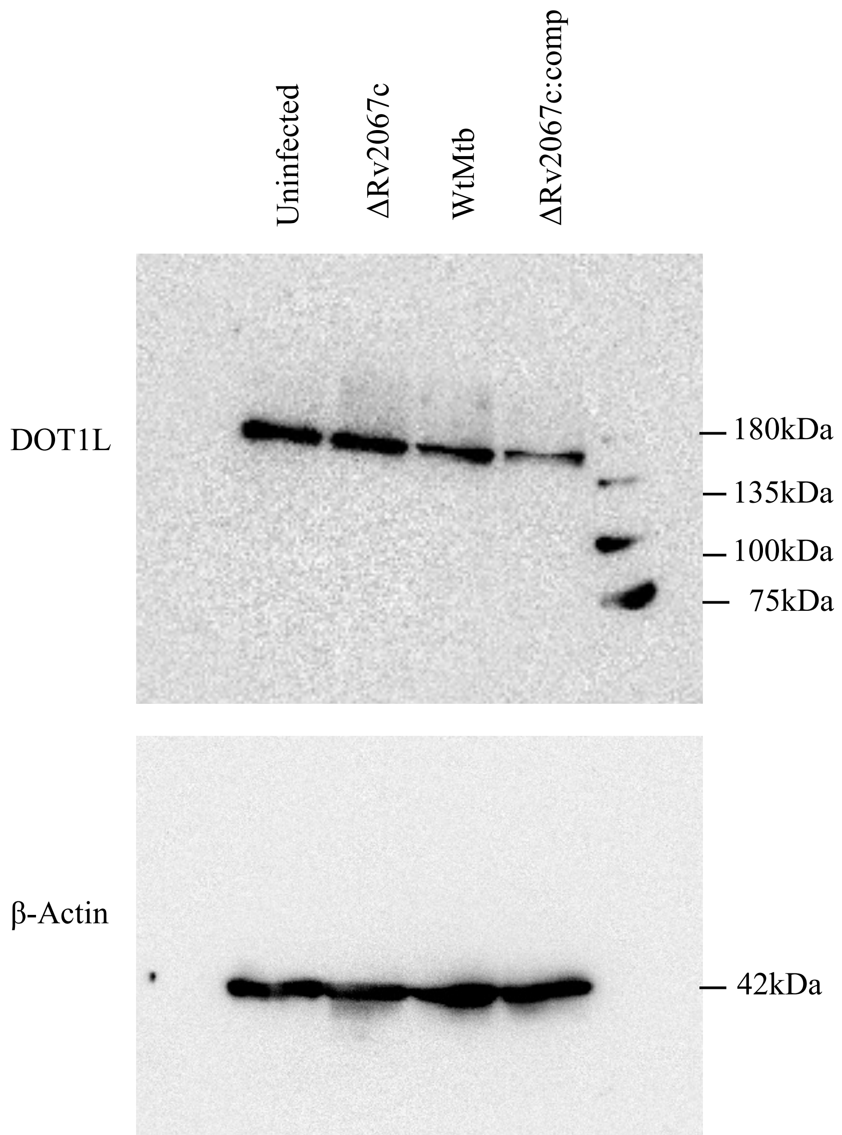

24 h.p.i

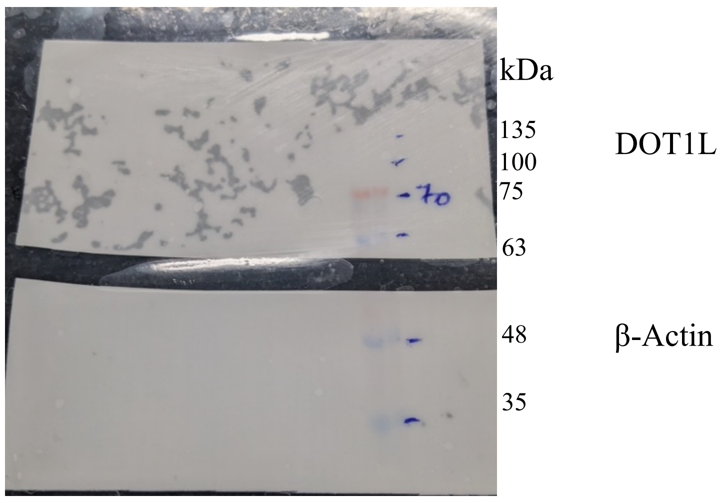

24 h.p.i

Blot was cut at 63kDa and probed with DOT1L (upper half) and β-actin (lower half)

Fig. 7: Rv2067c modulates DOT1L expression

Fig . 7c

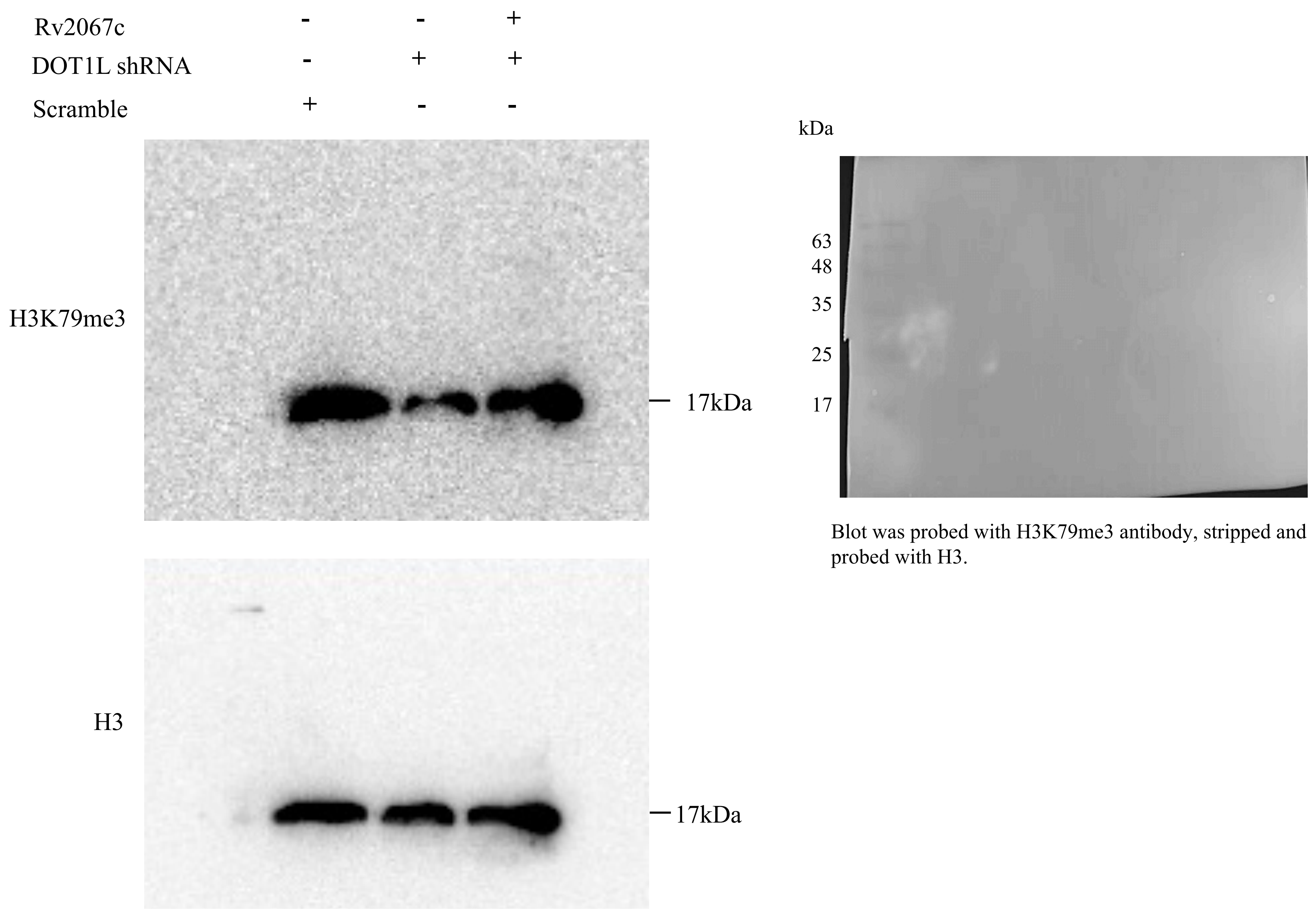

Fig . 7e

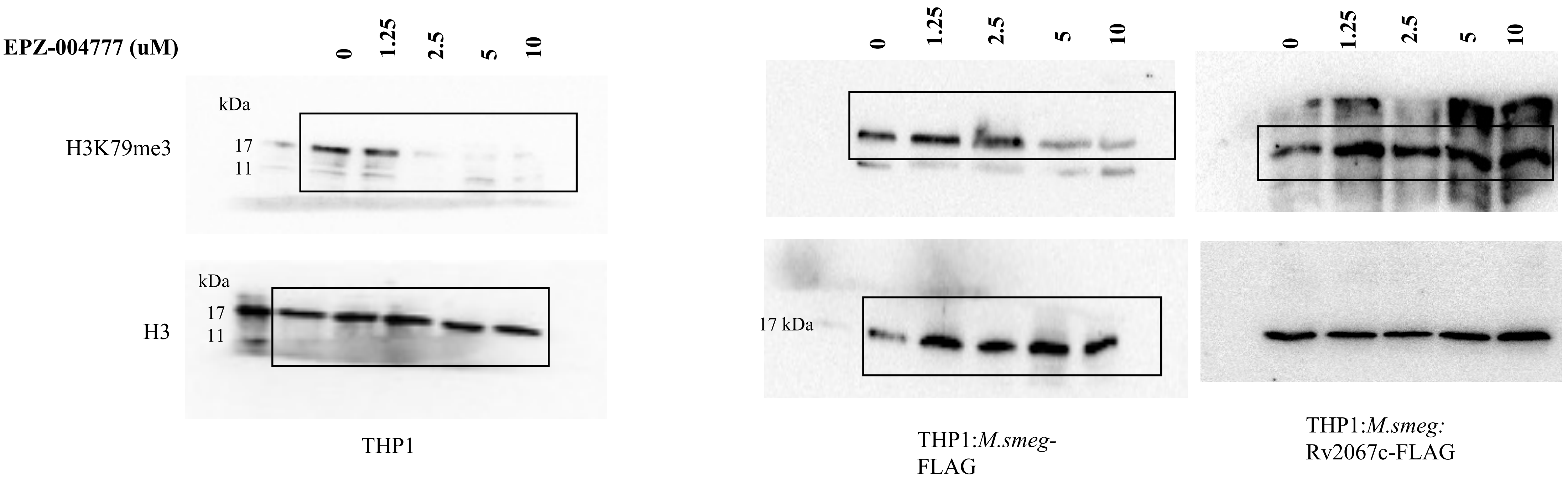

Fig. 7: Rv2067c modulates DOT1L expression

Fig. 8e

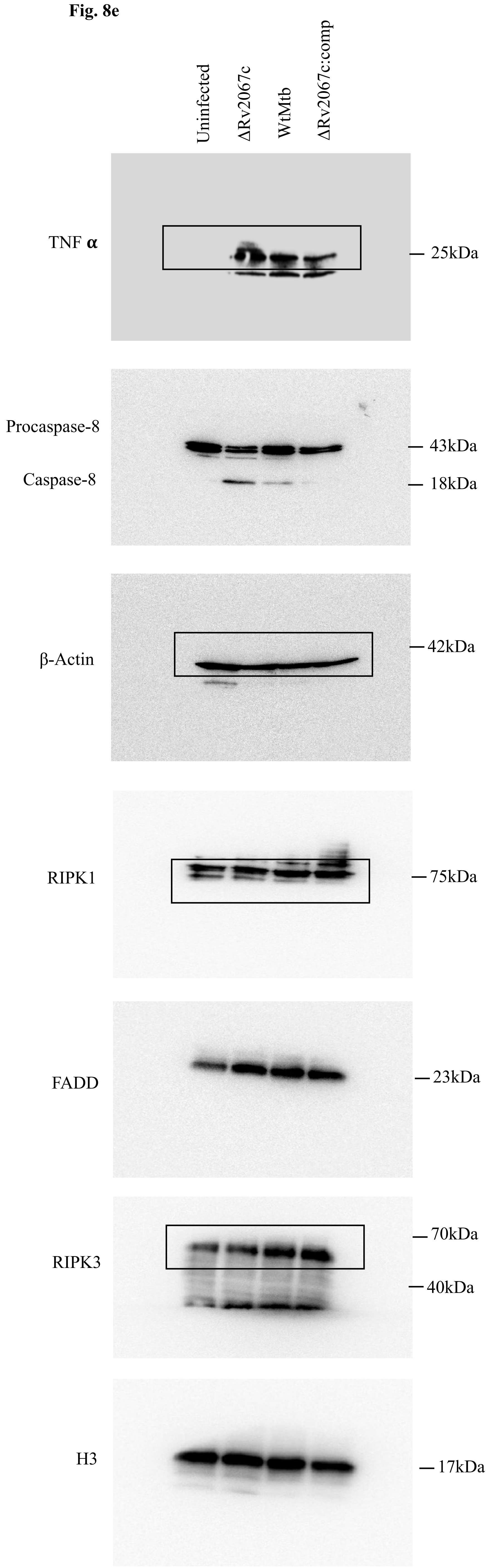

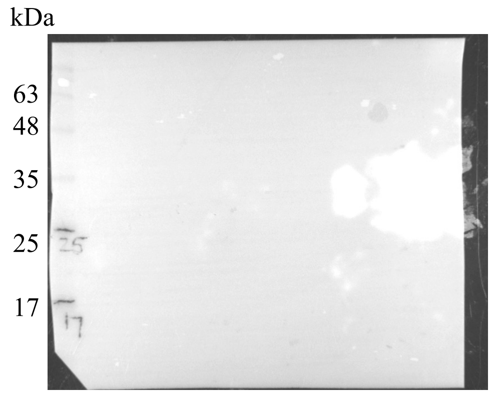

Probed with TNF**α**, caspase 8 and β-actin antibodies. Blot was stripped and reprobed with the mentioned antibodies

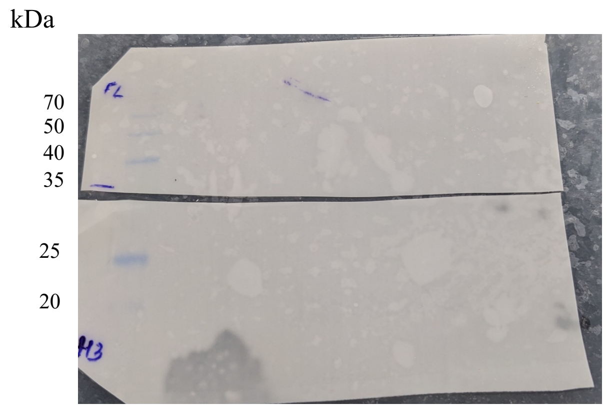

Probed with RIPK3, stripped and probed RIPK1 antibody.

Probed with FADD, stripped and probed H3 antibody.

Blot was cut between 35 kDa

Fig. 8:. Downstream events consequent to H3K79 methylation by Rv2067c

Fig. 8f

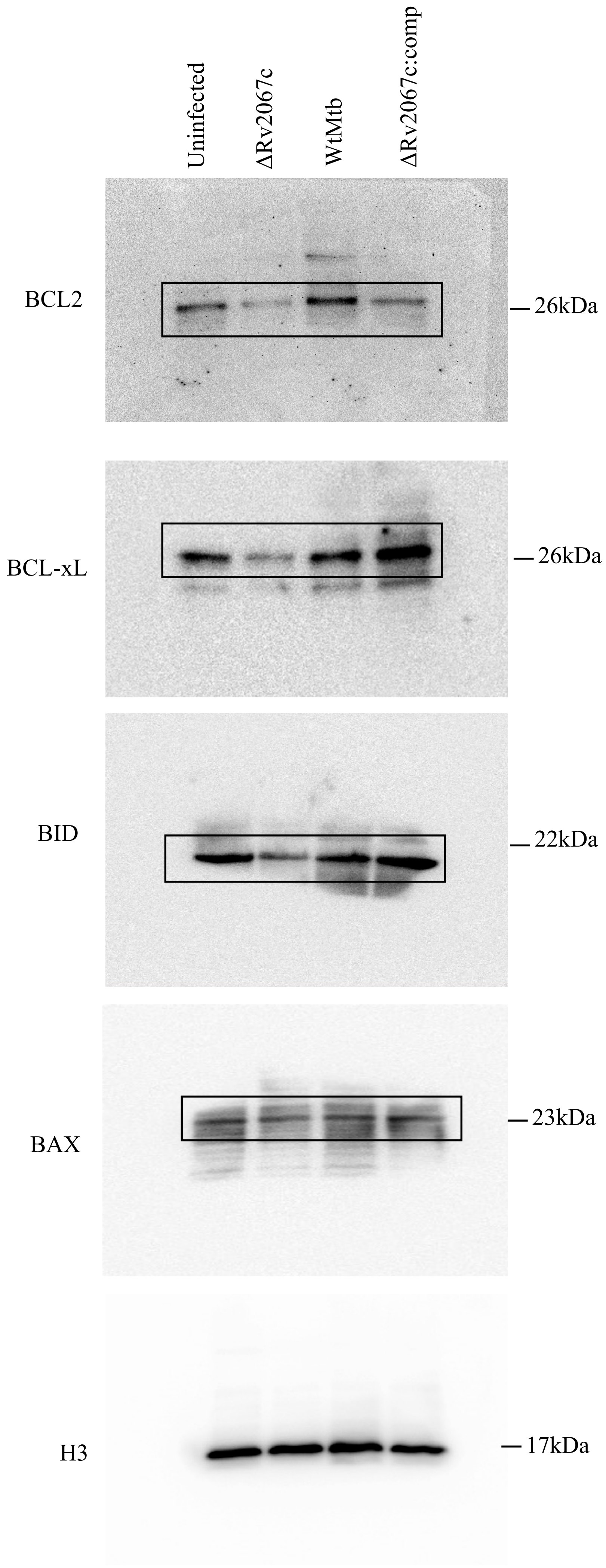

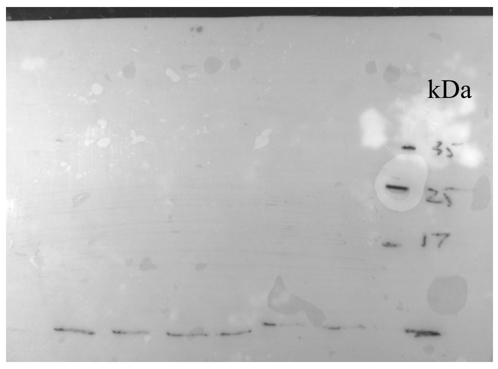

Blot was stripped and reporbed with different antibodies

Fig. 8:. Downstream events consequent to H3K79 methylation by Rv2067c

Supplementary Fig.1e

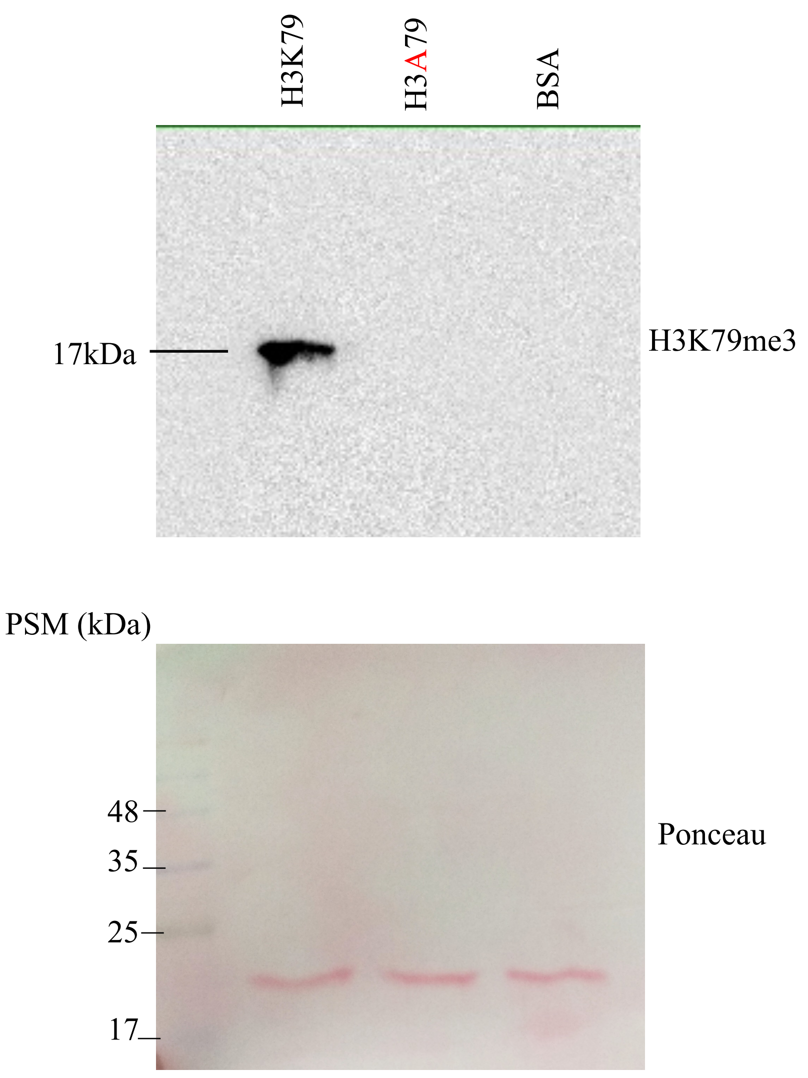

Supplementary Fig.1f

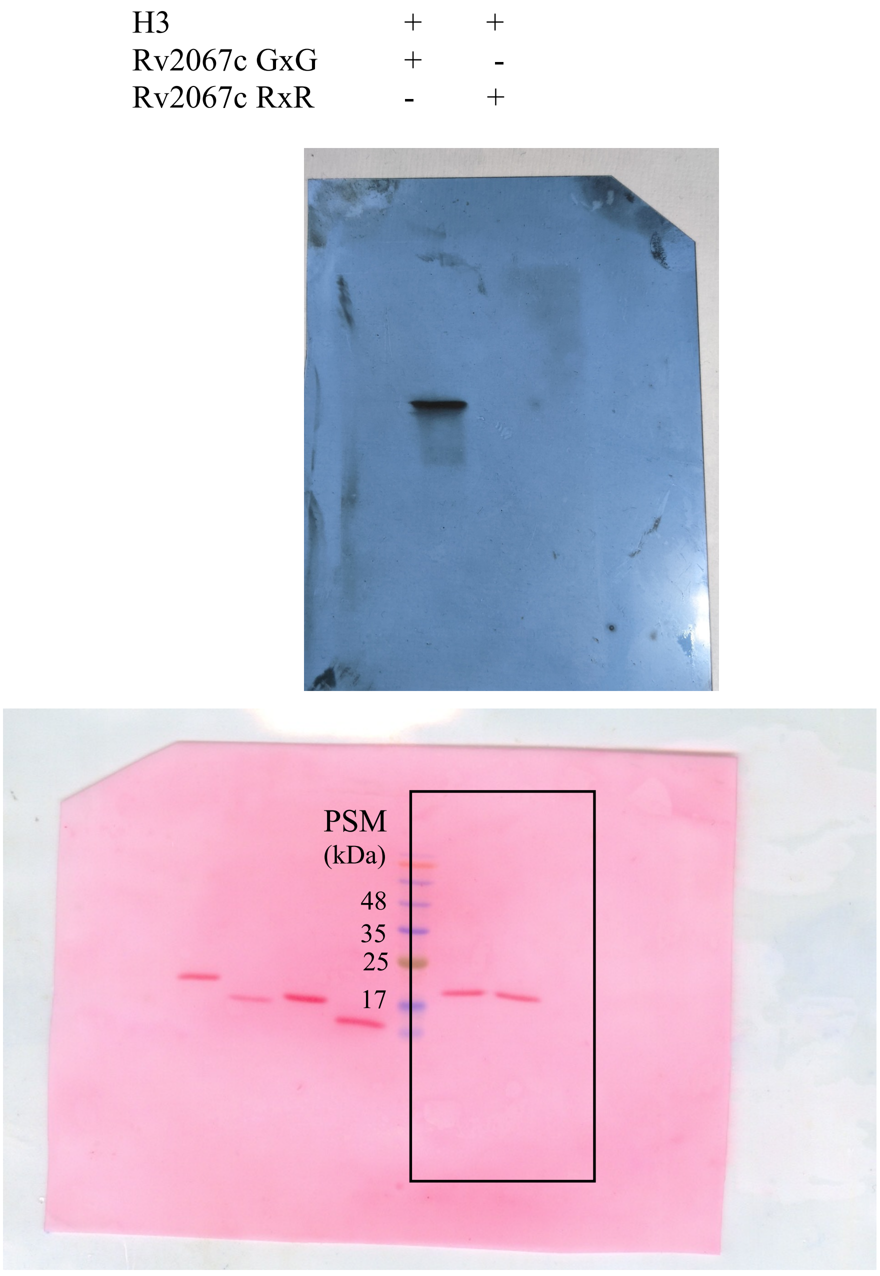

Supplementary Fig. 1i

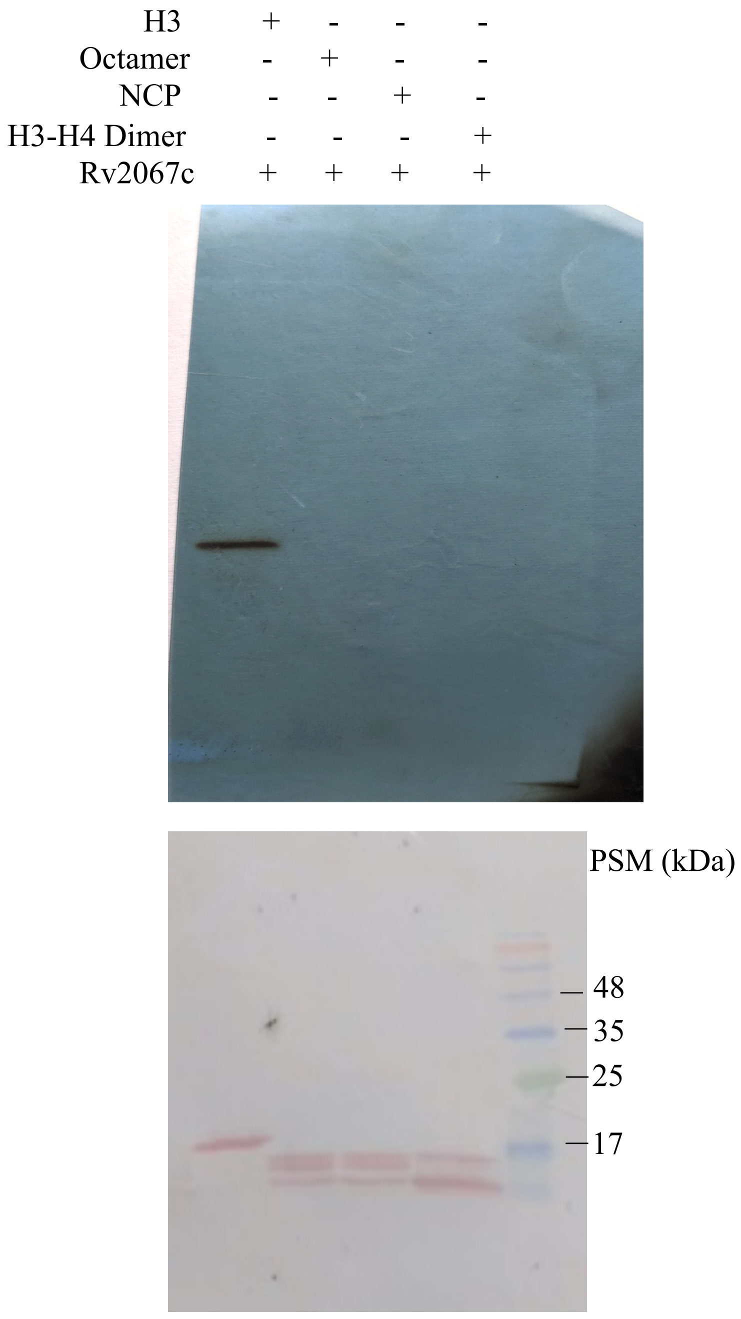

Supplementary Fig. 1: Rv2067c methylates histone H3 at Lysine 79

Supplementary Fig. 2a

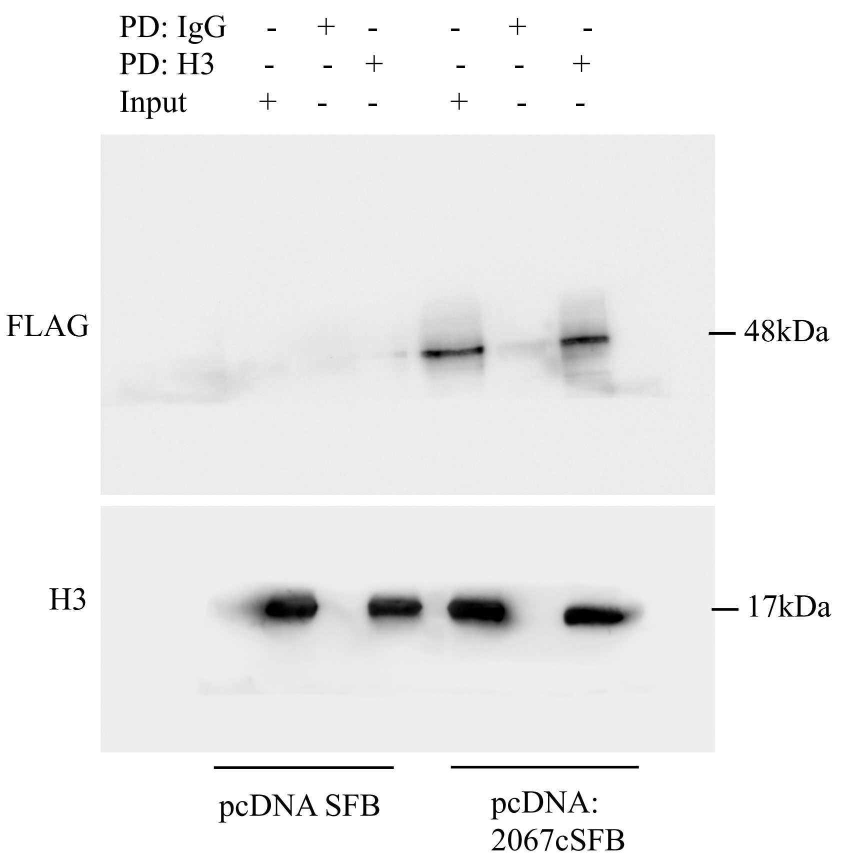

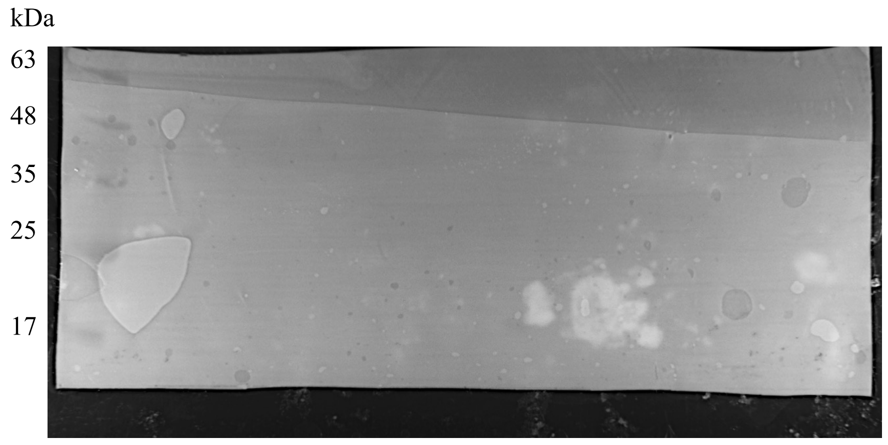

Blot was cut at 25 kDa and probed with FLAG (top half) and H3 (lower half) antibodies.

Supplementary Fig. 2b

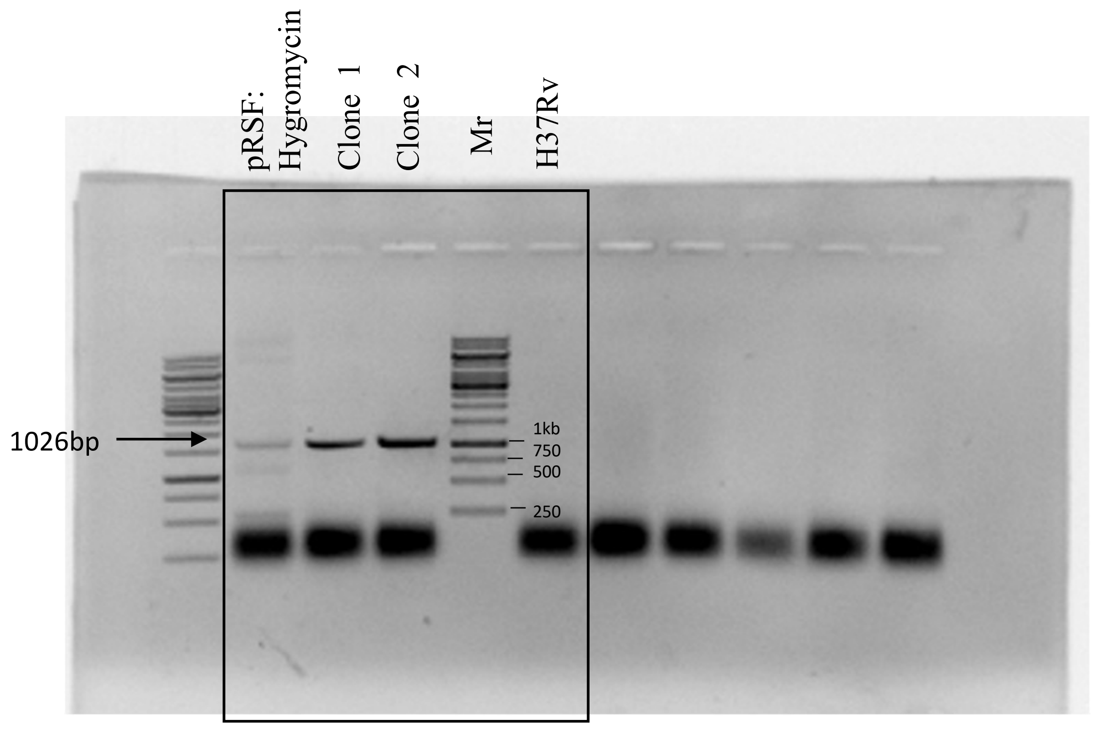

Hygromycin specific primers

Supplementary Fig. 2c

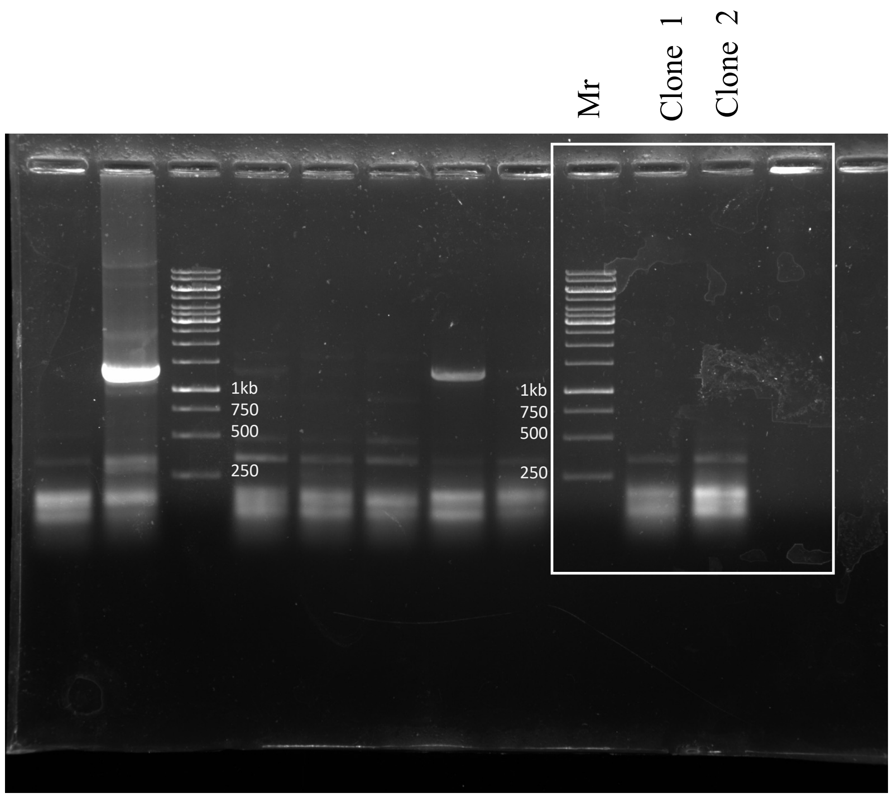

Internal primers for Rv2067c

Supplementary Fig. 2d

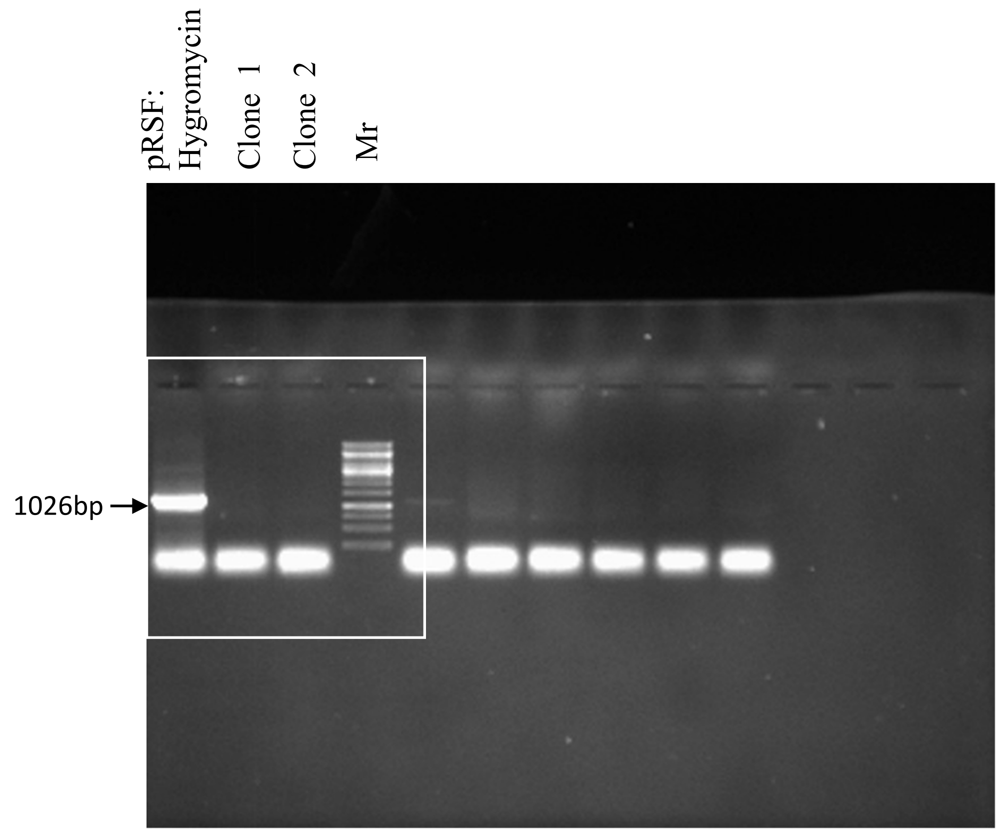

Hygromycin specific primers

Supplementary Fig. 2: Rv2067c methylates histone H3 at Lysine 79

Supplementary Fig. 2f

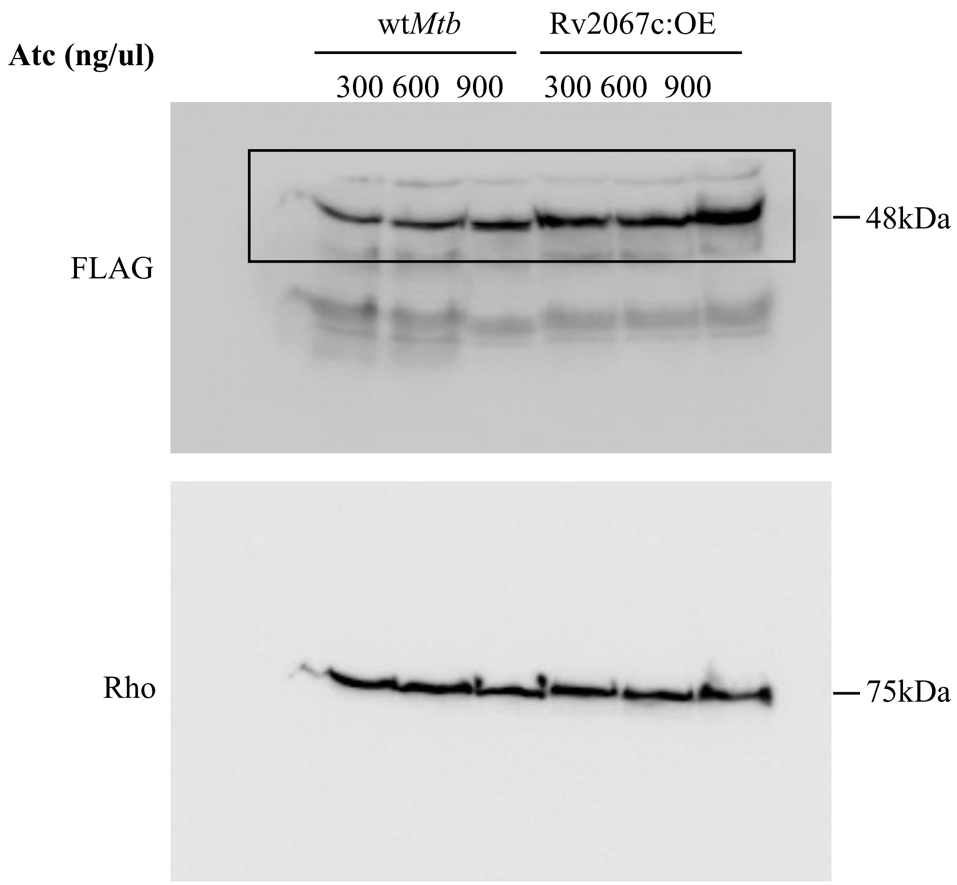

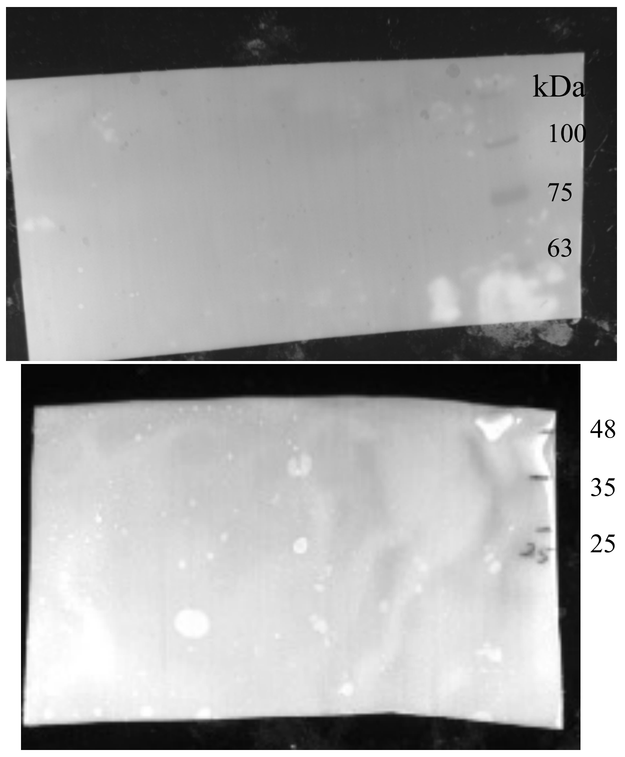

Blot was cut in-between 63 and 48 kDa and probed with rho (upper half) and FLAG (lower half)

Supplementary Fig. 2: Rv2067c methylates histone H3 at Lysine 79

Supplementary Fig. 3a

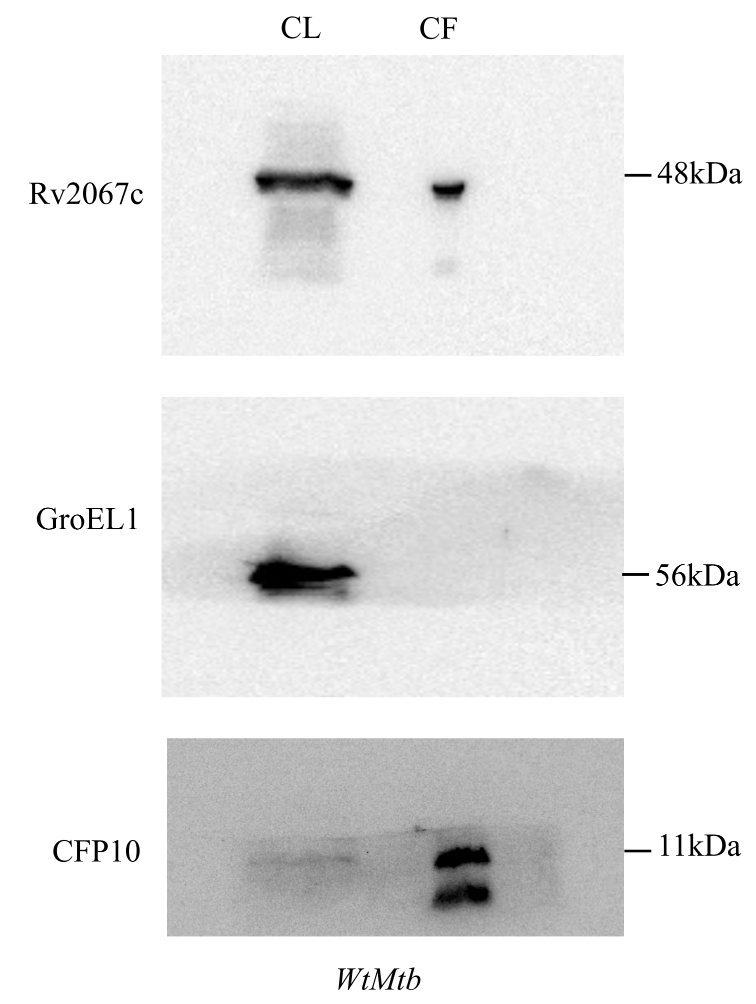

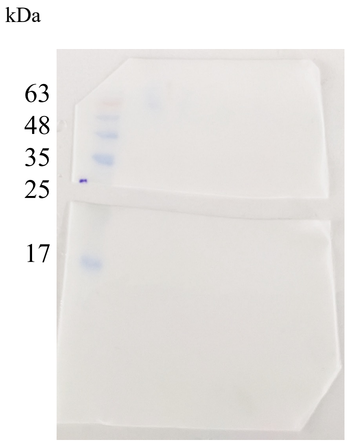

Blot was cut at 25 kDa. The top half was probed Rv2067c, stripped and probed with GroEL1 antibody; lower half was probed with H3 antibody.

Supplementary Fig. 3b

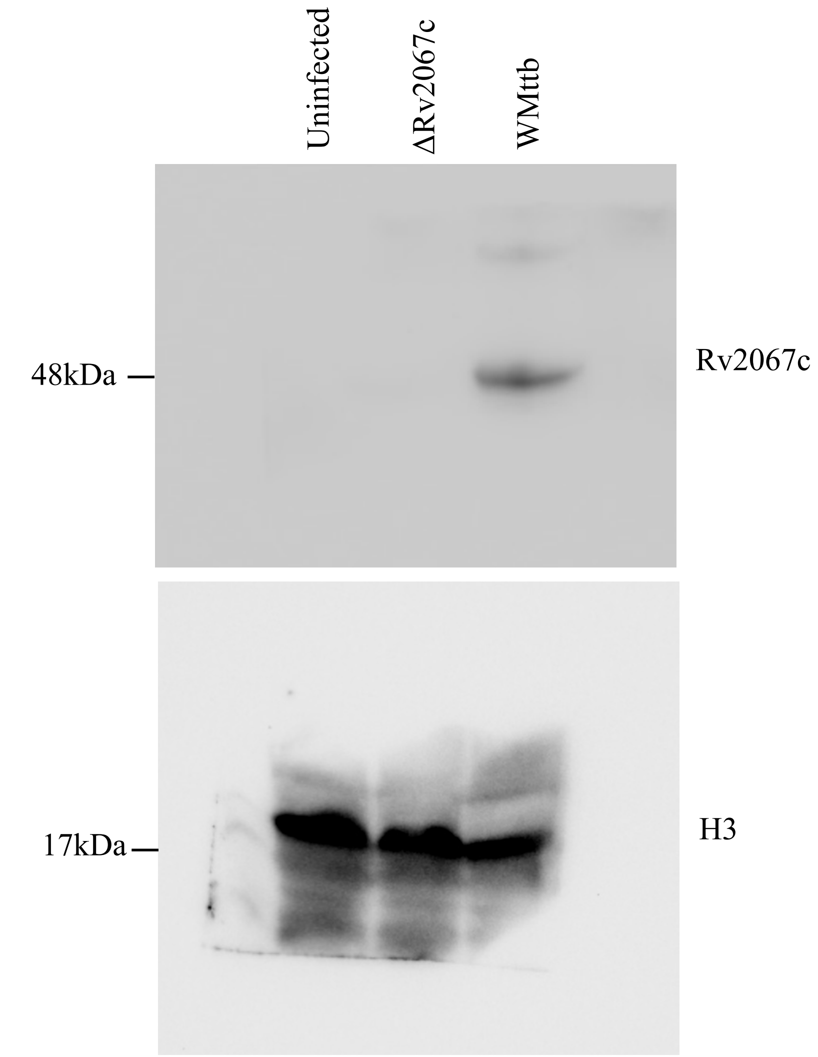

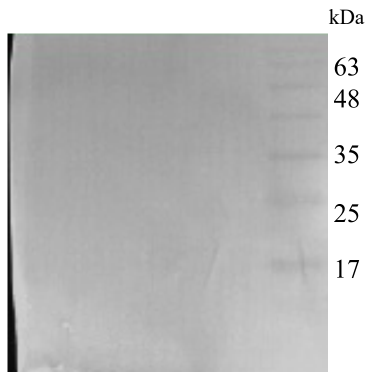

Blot was cut at 25 kDa and probed with Rv2067c (top half) and H3 (lower half) antibodies

Supplementary Fig. 3: Secretion and localization of Rv2067c

Supplementary Fig. 3c

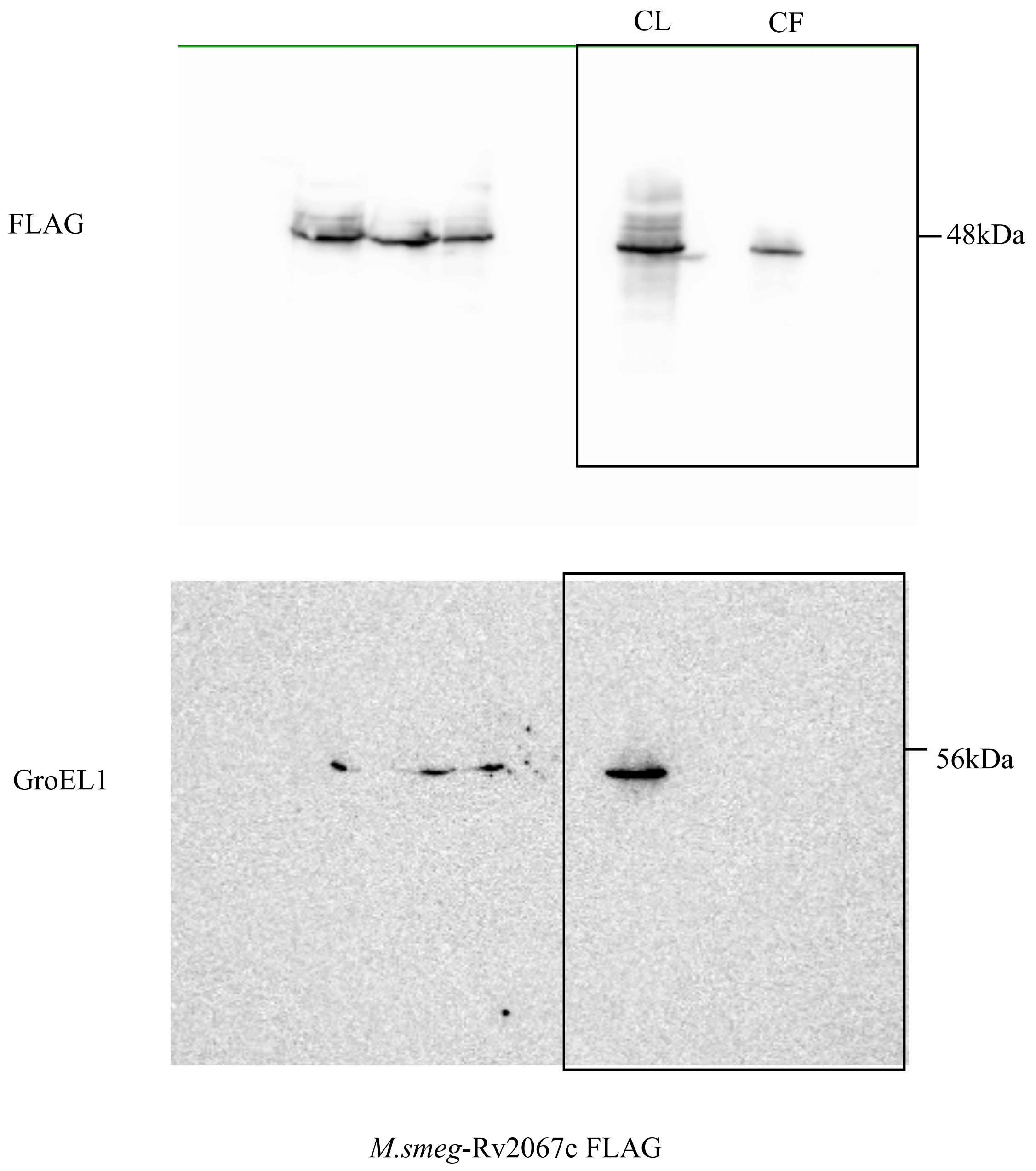

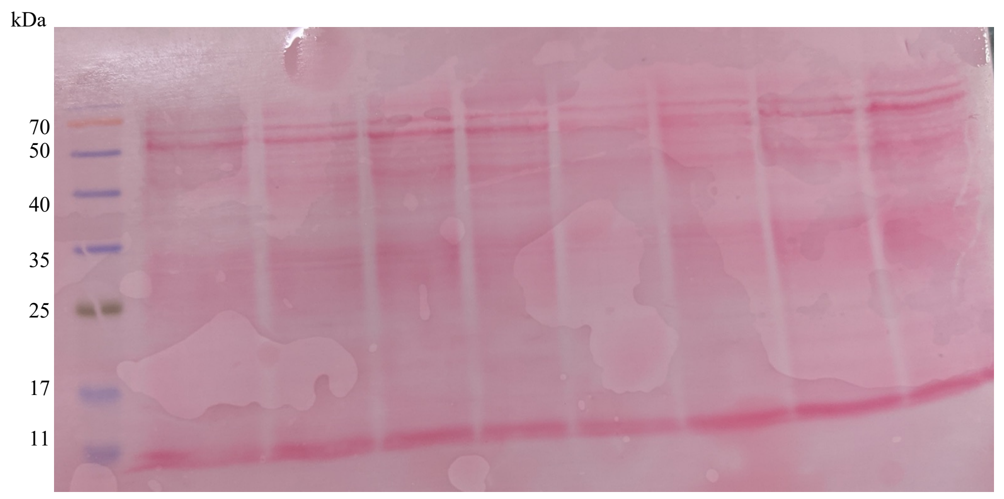

Ponceau staining : Blot was probed with FLAG, stripped and probed with GroEL1 antibody

Supplementary Fig. 3e

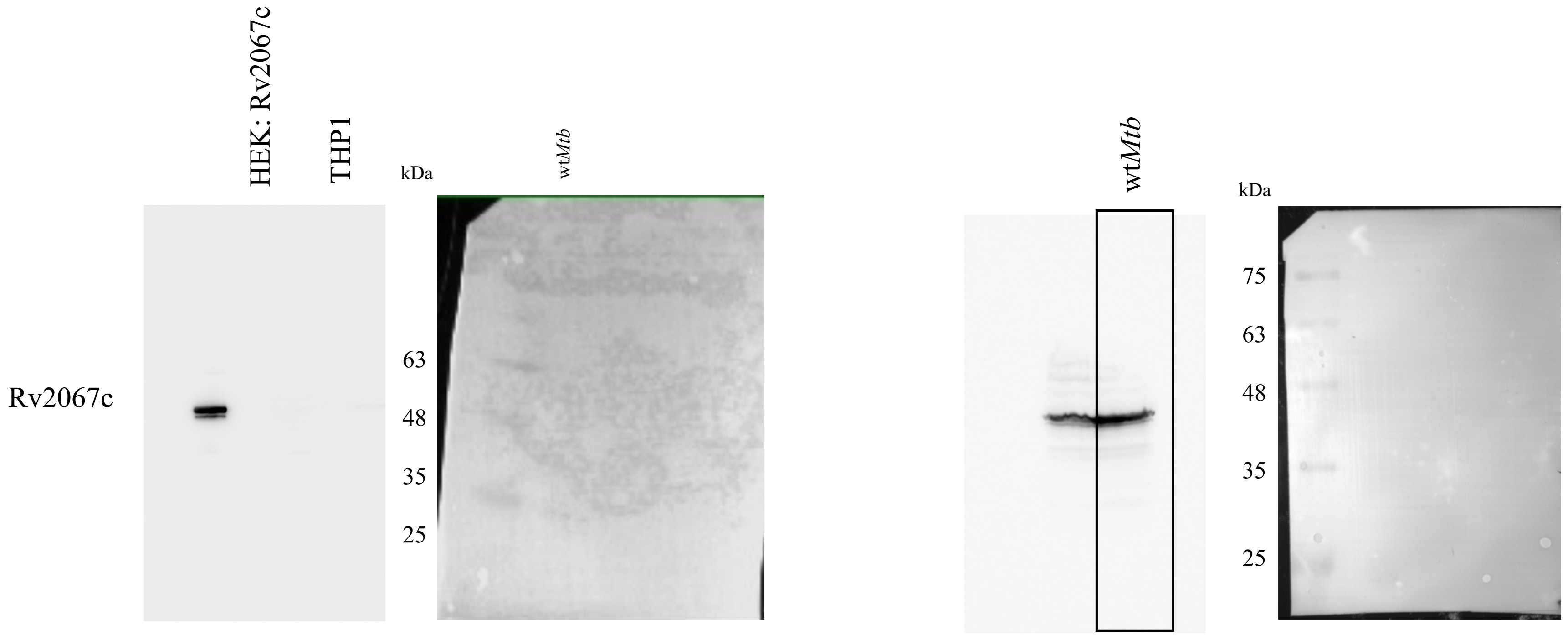

Supplementary Fig. 3: Secretion and localization of Rv2067c

Supplementary Fig. 3h

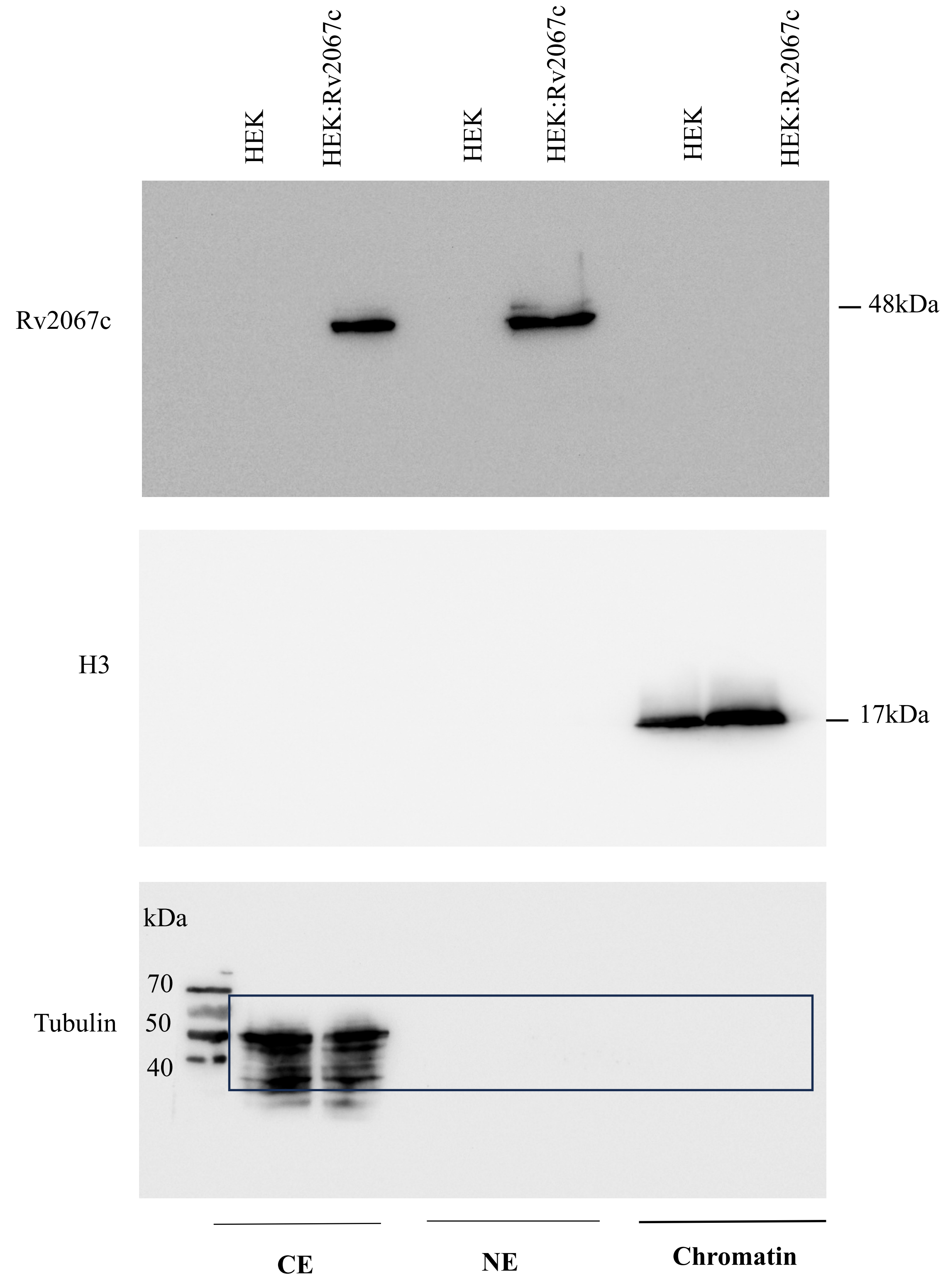

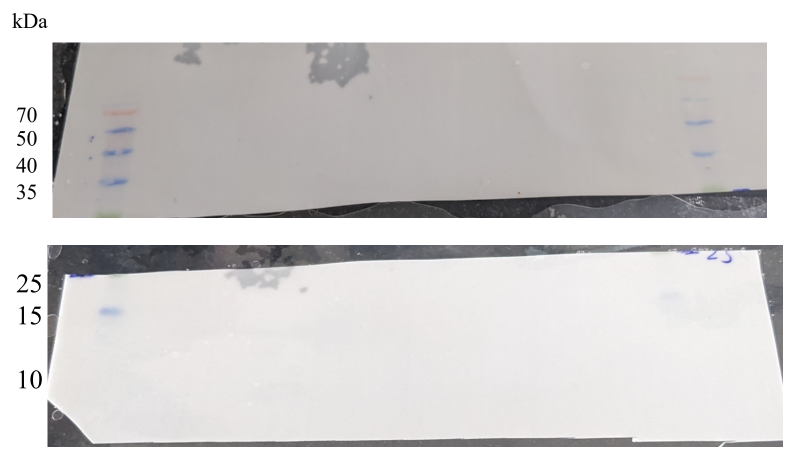

Blot was cut at 25 kDa. The top half of the blot was probed with Rv2067c, stripped and probed with tubulin antibody. The lower half was probed with H3 antibody.

Supplementary Fig. 3: Secretion and localization of Rv2067c

Supplementary Fig. 3j

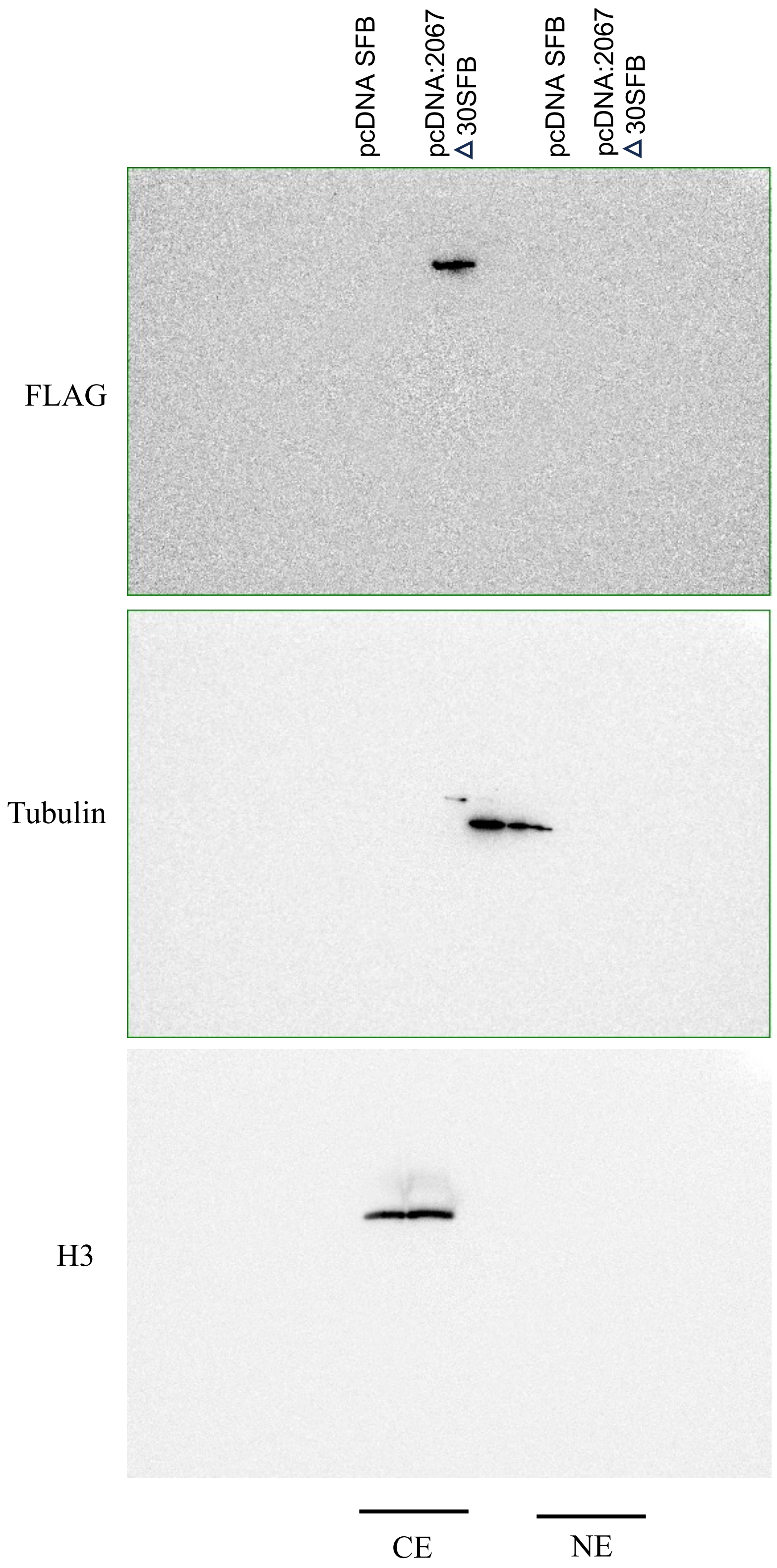

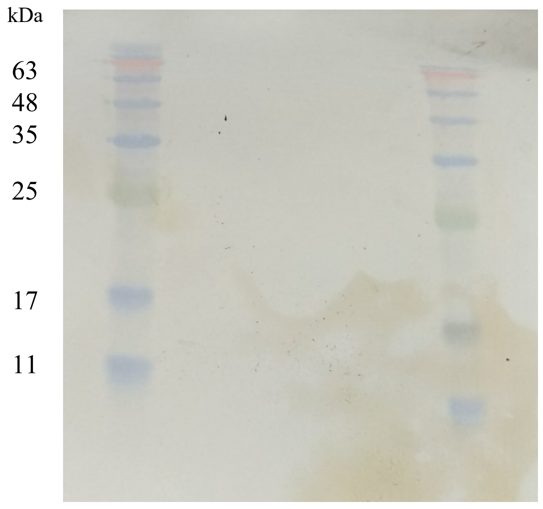

Blot was cut at 25 kDa. Upper blot was probed with FLAG, stripped and probed with tubulin antibody. Lower blot was probed with H3 antibody

Supplementary Fig. 3: Secretion and localization of Rv2067c

Supplementary Fig. 13a

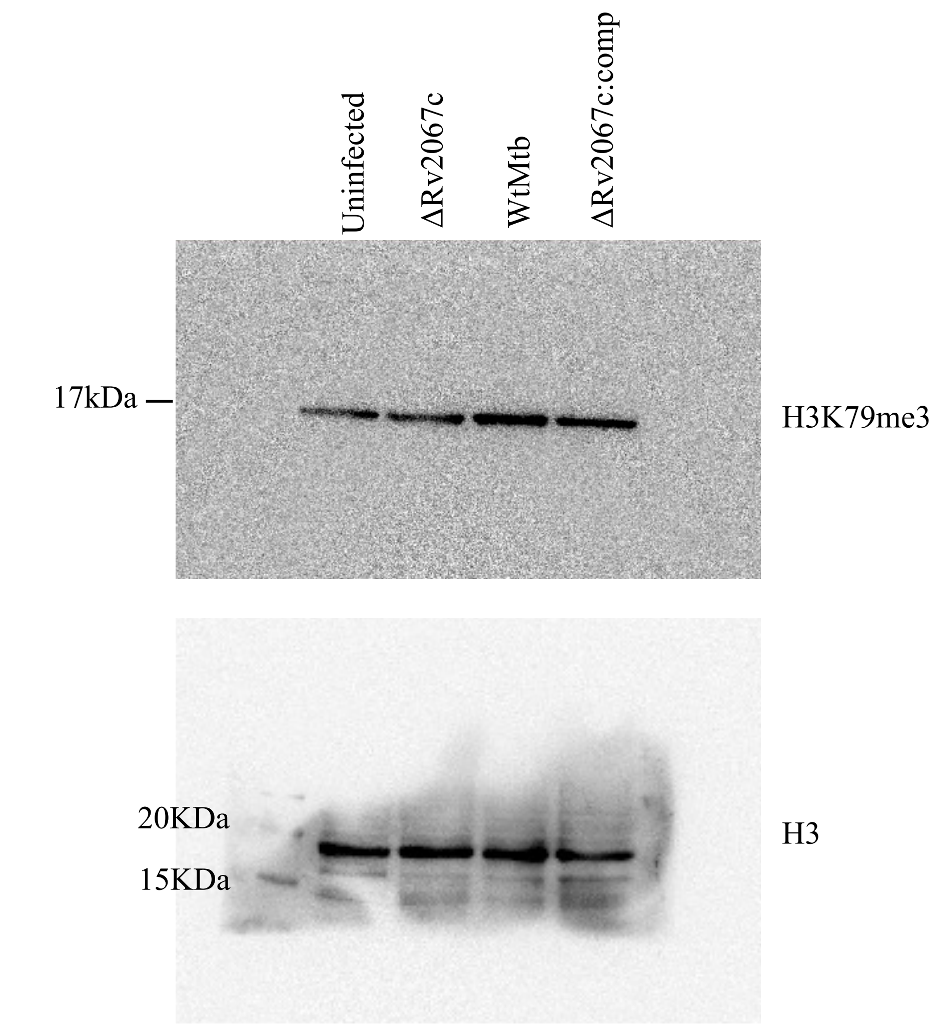

12h.p.i

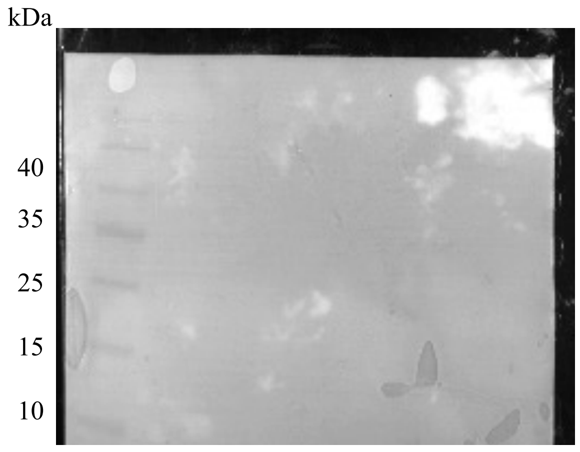

Blot was probed with H3, stripped and probed with H3K79me3 antibody

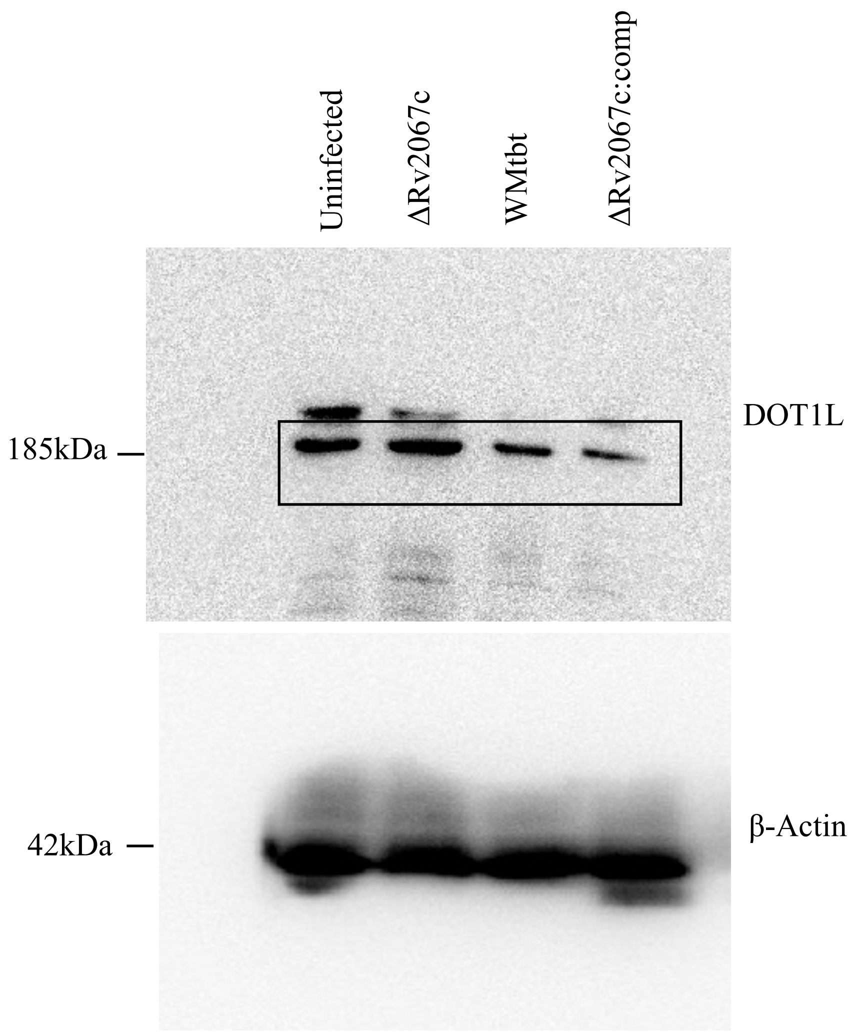

12h.p.i

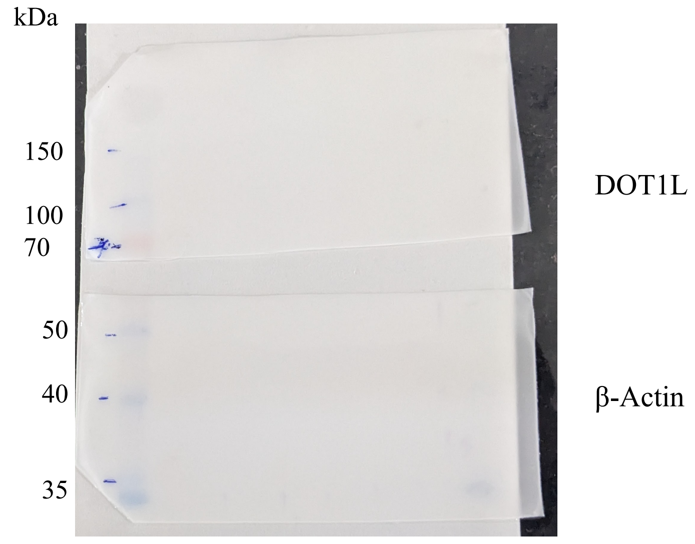

Blot was cut at 70 kDa. The top half of the blot was probed with DOT1Lantibody. The lower half was probed with β-Actin antibody.

Supplementary Fig. 13: Rv2067c modulates DOT1L expression

Supplementary Fig. 13b

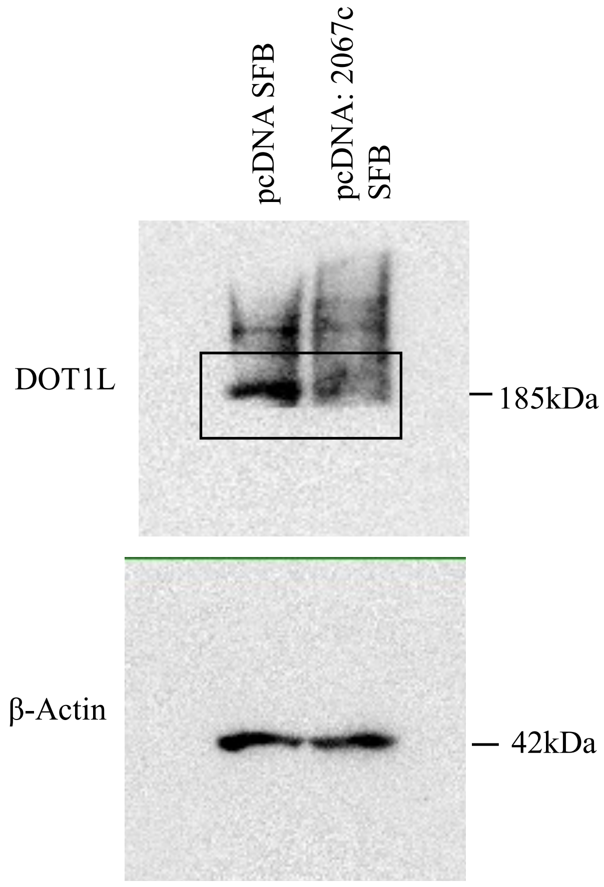

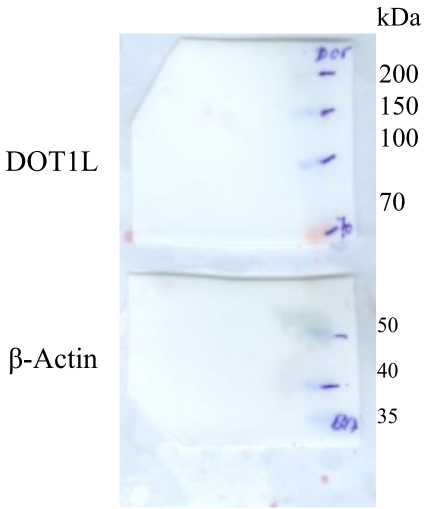

Blot was cut at 70 kDa. The top half of the blot was probed with DOT1Lantibody. The lower half was probed with β-Actin antibody.

Supplementary Fig. 13c

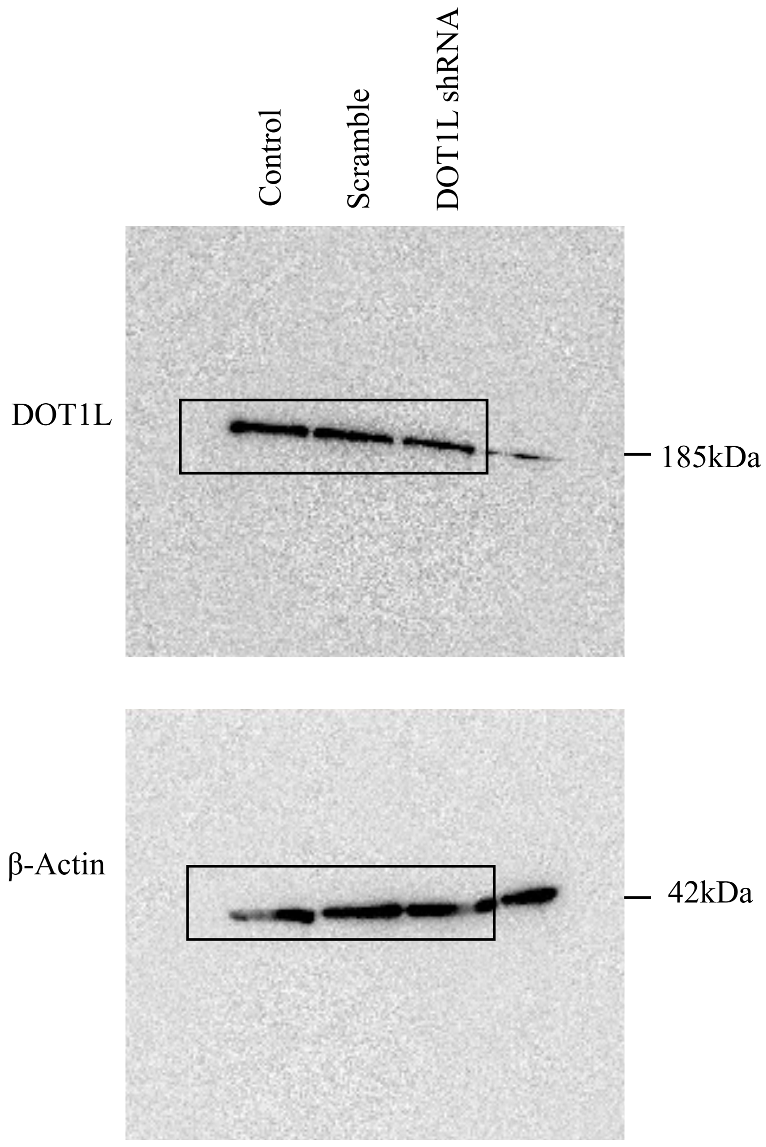

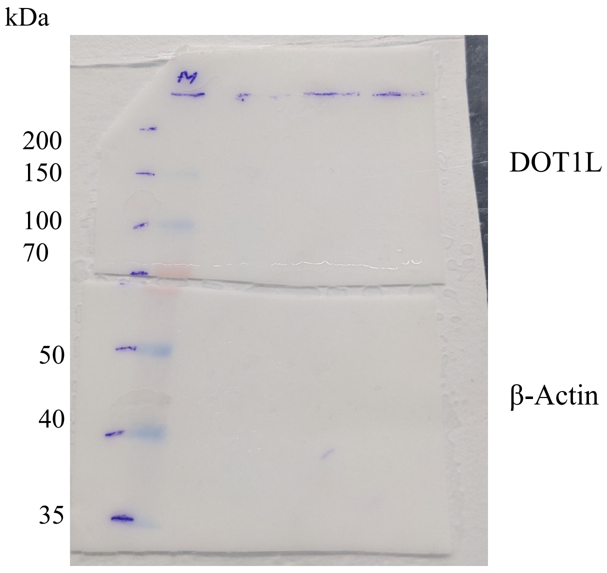

Blot was cut at 70 kDa. The top half of the blot was probed with DOT1L antibody. The lower half was probed with β-Actin antibody.

Supplementary Fig. 14a

**Scr siRNA**	+	-
**DOT1L siRNA**	-	+

DOT1L

β-Actin

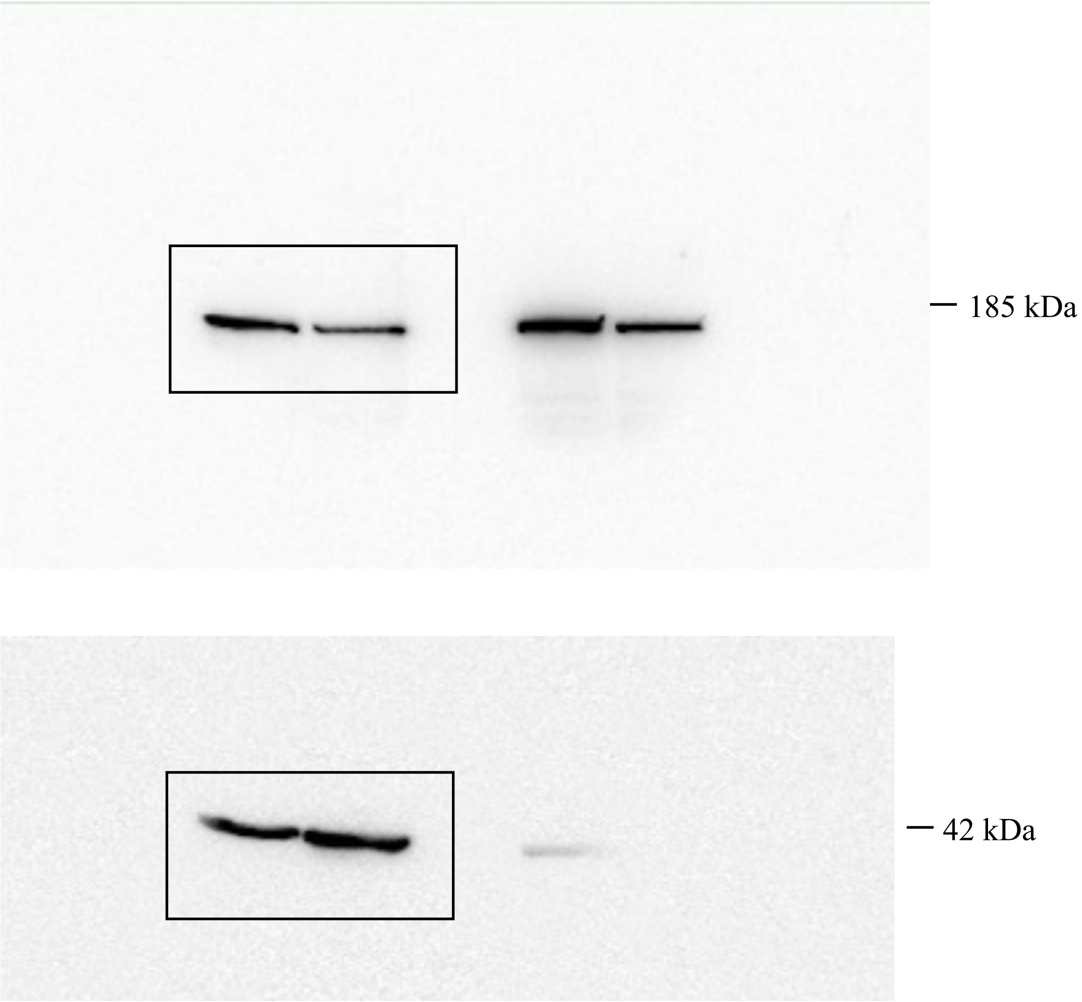

— 185 kDa

— 42 kDa

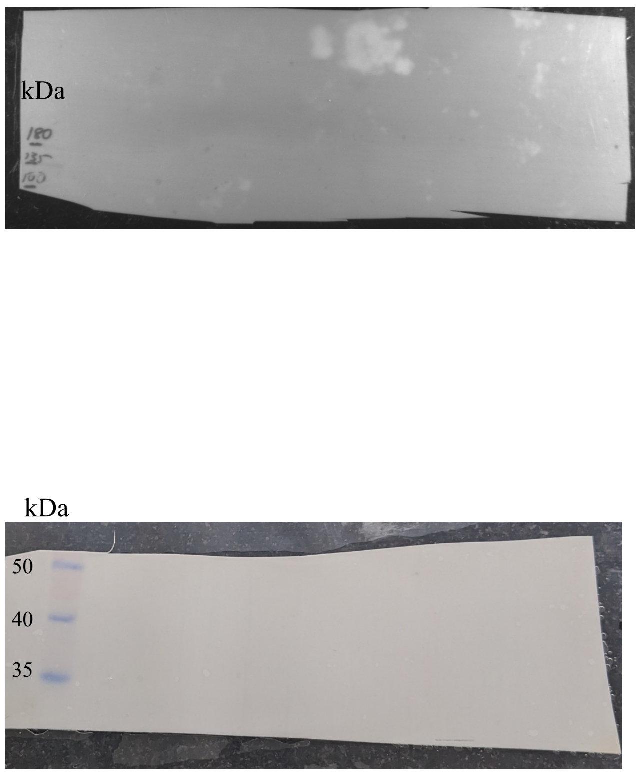

DOT1L

β-Actin

Blot was cut at 70 kDa. The top half of the blot was probed with DOT1Lantibody. The lower half was probed with β-Actin antibody.

Supplementary Fig. 14: Downstream events consequent to H3K79 methylation by Rv2067c

Supplementary Fig. 14 K: Gating strategy for FACS

ΔRv2067c:comp

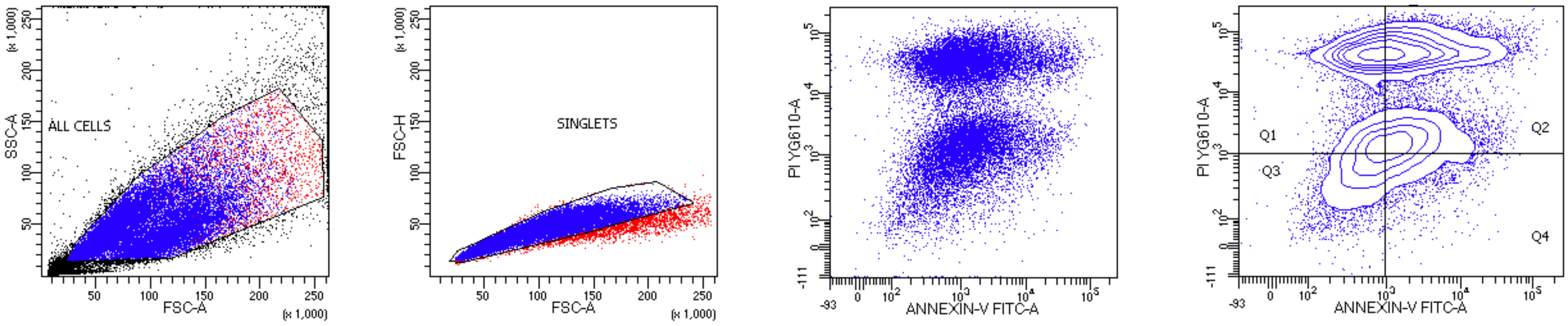

Wt*Mtb*

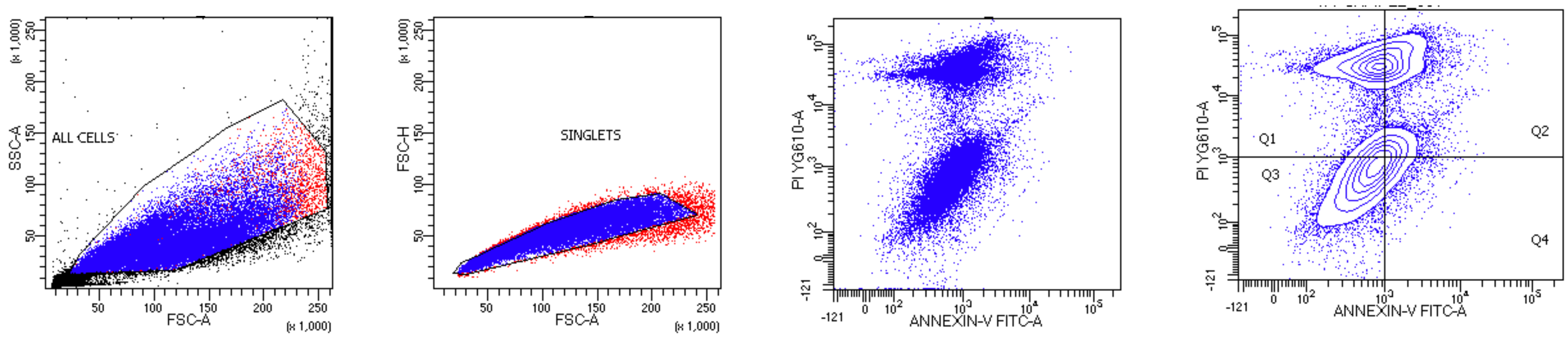

ΔRv2067c

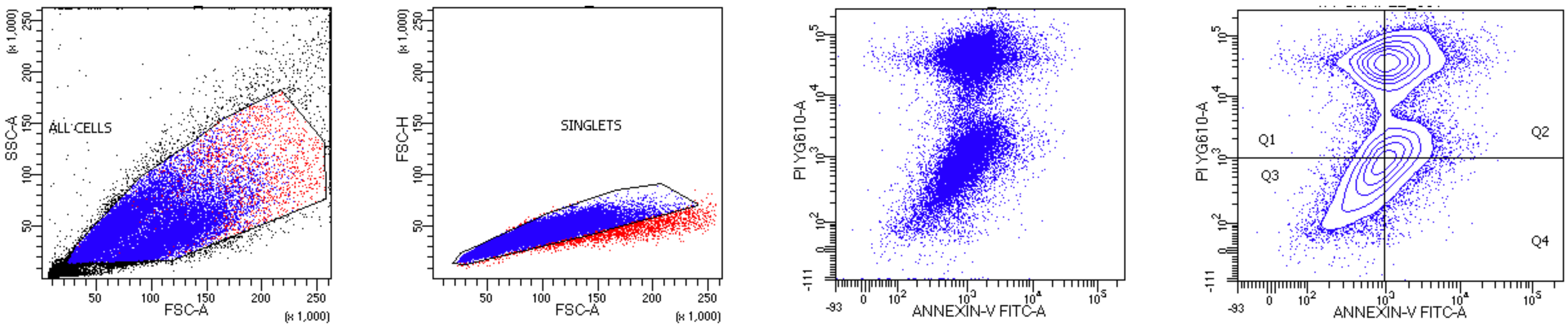

Supplementary Fig. 14: Downstream events consequent to H3K79 methylation by Rv2067c
